# Towards a comprehensive picture of alloacceptor tRNA remolding in metazoan mitochondrial genomes

**DOI:** 10.1093/nar/gkv746

**Published:** 2015-07-30

**Authors:** Abdullah H. Sahyoun, Martin Hölzer, Frank Jühling, Christian Höner zu Siederdissen, Marwa Al-Arab, Kifah Tout, Manja Marz, Martin Middendorf, Peter F. Stadler, Matthias Bernt

**Affiliations:** 1 Bioinformatics Group, Department of Computer Science, Leipzig University, Härtelstraße 16-18, D-04107 Leipzig, Germany; 2 Interdisciplinary Center for Bioinformatics, Leipzig University, Härtelstraße 16-18, D-04107 Leipzig, Germany; 3 Doctoral School of Science and Technology, AZM Center for Biotechnology Research, Lebanese University, Tripoli, Lebanon; 4 RNA Bioinformatics and High Throughput Analysis, Faculty of Mathematics and Computer Science, Friedrich Schiller University Jena, Leutragraben 1, 07743 Jena, Germany; 5 Bioinformatics Unit and Department of Chromatin Regulation, Max Planck Institute of Immunobiology and Epigenetics, Stübeweg 51, 79108 Freiburg, Germany; 6 Transcriptome Bioinformatics, LIFE Research Center for Civilization Diseases, Leipzig University, Härtelstraße 16-18, D-04107 Leipzig, Germany; 7 Department of Theoretical Chemistry, University of Vienna, Währingerstraße 17, A-1090 Wien, Austria; 8 FLI Leibniz Institute for Age Research, Beutenbergstraße 11, 07745 Jena, Germany; 9 Michael Stifel Center Jena, Ernst-Abbe-Platz 2, 07743 Jena, Germany; 10 Parallel Computing and Complex Systems Group, Department of Computer Science, Leipzig University, Augustusplatz 10, D-04109 Leipzig, Germany; 11 Max-Planck-Institute for Mathematics in the Sciences, Inselstraße 22, D-04103 Leipzig, Germany; 12 Fraunhofer Institut für Zelltherapie und Immunologie Perlickstraße 1, D-04103 Leipzig, Germany; 13 Center for non-coding RNA in Technology and Health, University of Copenhagen, Grønnegårdsvej 3, DK-1870 Frederiksberg, Denmark; 14 Santa Fe Institute, 1399 Hyde Park Rd., Santa Fe, NM 87501, USA

## Abstract

Remolding of tRNAs is a well-documented process in mitochondrial genomes that changes the identity of a tRNA. It involves a duplication of a tRNA gene, a mutation that changes the anticodon and the loss of the ancestral tRNA gene. The net effect is a functional tRNA that is more closely related to tRNAs of a different alloacceptor family than to tRNAs with the same anticodon in related species. Beyond being of interest for understanding mitochondrial tRNA function and evolution, tRNA remolding events can lead to artifacts in the annotation of mitogenomes and thus in studies of mitogenomic evolution. Therefore, it is important to identify and catalog these events. Here we describe novel methods to detect tRNA remolding in large-scale data sets and apply them to survey tRNA remolding throughout animal evolution. We identify several novel remolding events in addition to the ones previously mentioned in the literature. A detailed analysis of these remoldings showed that many of them are derived from ancestral events.

## INTRODUCTION

Mitochondria are organelles that are found in almost all eukaryotic cells. They produce most of the cell's adenosine triphosphate (ATP) supply and are involved in central processes such as the control of the cell cycle, growth (1) and death (2).

The mitochondrial genome (mitogenome) of almost all animal (Metazoa) species encodes for a conserved set of 13 protein coding genes, two ribosomal RNAs (rRNAs) and a set of 22 transfer RNAs (tRNAs). With the two exceptions of leucine and serine, only a single tRNA for each amino acid is present. Independent losses of tRNAs, which are compensated by tRNAs imported from the nucleus (3), are frequent in a few clades, e.g. Cnidaria (4), and Chaetognatha (5). Mitochondrial tRNAs of Metazoa frequently exhibit aberrant structures. For example, many tRNAs lack the otherwise highly conserved D-loops and/or T-loops (6), or complete arms (7,8). Even the loss of both D- and T-arm have been reported for Enoplea (9), see (10) for an overview.

Mitochondrial tRNAs are able to translate all proteins encoded in the mitogenome (11). The leucine and serine tRNAs fall into two classes each:

- *trnL1* and *trnL2* with anticodon UAG and UAA that recognize codons CUN and UUR, respectively, and
- *trnS1* and *trnS2* with anticodon GCU (UCU in approx. 37% of the Protostomia) and UGA that recognize codons AGY and UCN, respectively.

The anticodon is usually well preserved throughout Metazoa and corresponds to the codon with

- an A at the third position if possible (i.e. for fourfold degenerate codons and two-fold degenerate codon boxes ending with a purine) and
- a G at the third position otherwise (i.e. for two-fold degenerate codon boxes ending with a pyrimidine).

The only exception to this rule is *trnM (AUG)* with the anticodon CAU, which corresponds to the most frequently used start codon. Furthermore, for *trnK*, *trnS1* and *trnR* alternative anticodons are frequent in some taxa. For non-mitochondrial genetic systems, the ability of a reduced number of tRNAs to recognize all codons has been explained by the wobble hypothesis, which states that a non-standard base pairing (G-U) could occur at the third codon position, i.e. the first position of the anticodon. According to the wobble hypothesis, only 32 tRNAs are required to recognize all codons. However, given the set of 22 mitochondrial tRNAs, relaxed wobble rules (‘super wobble’) have been suggested as an explanation. The idea is that tRNA species with an unmodified U in the wobble position of the anticodon are able to read all four nucleotides in the third codon position. See (12) for a review.

The genetic code is determined by the assignment of

- tRNAs to codons, via the anticodon and
- tRNAs to amino acid, i.e. aminoacylation, via identity elements of the tRNAs.

The mitochondrial genetic code is known to differ from the standard genetic code, and multiple variations are known for eukaryotes. Codon reassignments have been explained by one or a mixture of several different hypothetical mechanisms (13):

- *Codon capture* by a tRNA coding for a different amino acid after the codon was completely lost in the mitogenome and later reappeared;
- Two *ambiguous intermediate* tRNAs that can translate multiple codons;
- A tRNA gains the ability to decode codons that became *unassigned* due to the deletion of a tRNA;
- *Compensatory change*, i.e. infrequent gain and loss in a population that spreads as soon as they coincide in the same individual.

Identity elements of mitochondrial tRNAs have been analyzed *in-vitro* for *trnR* (14), *trnD* (15), *trnY* (15,16), *trnL* (17), *trnS* (18,19) and *trnA* (20). For the remaining tRNAs, identity elements have been analyzed *in-silico* through sequence and structural conservation studies, and comparisons to known identity elements of *Escherichia coli* (21). In comparison to cytoplasmic tRNAs, mitochondrial tRNAs exhibit a reduced set of identity elements. For most tRNAs the anticodon or the discriminator base (at the 3’ end of the acceptor arm) are main identity elements. Only for *trnL*, *trnH*, *trnS* and *trnA* the anticodon is not an identity element. Most of the other known identity elements are found in the acceptor or the anticodon stem. A connection between alterations of tRNA identity by RNA editing and variation of the genetic code was discussed in (12,22). Hence a connection between genetic code changes and tRNA remolding is conceivable, but so far has remained unexplored.

**Figure 1.**
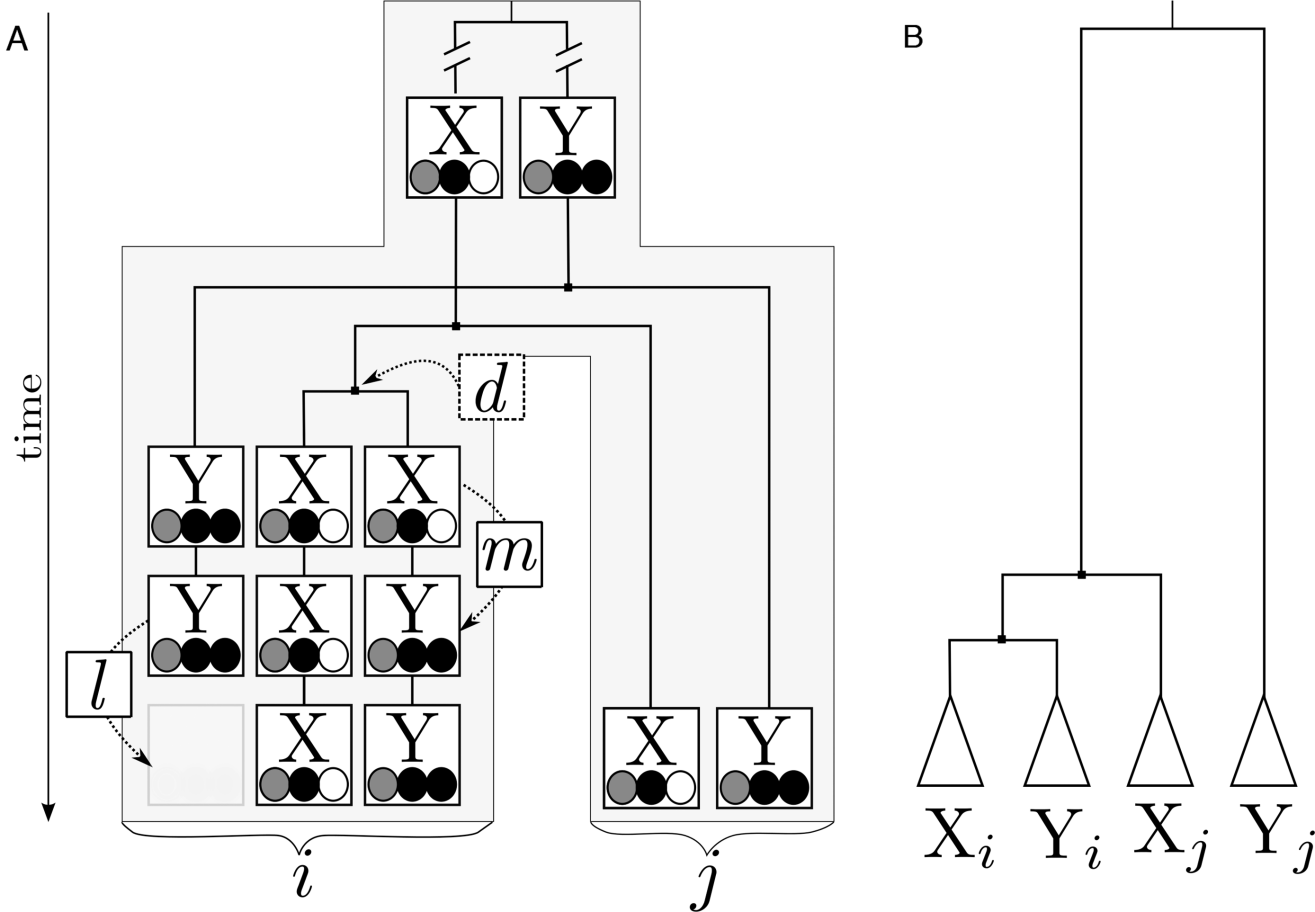
Schematic representation of an alloacceptor tRNA remolding event in a subtree *i* using the example of X↝Y. (**A**) The anticodon is represented by gray, black and white circles representing the nucleotides. Shown are the duplication (*d*), the point mutation in the third position of the anticodon which changes the identity of the tRNA (*m*), and the loss of the original *trnY* (*l*). The species tree for the affected subtree *i* and an unaffected sibling subtree *j* is shown in light gray. The gene tree for the tRNAs is represented by lines. (**B**) Phylogeny of the four tRNAs representing their evolutionary relationship.

A quite particular mode of evolution reserved to tRNAs is *remolding*, also known as *tRNA recruitment*, in which point mutation(s) in the anticodon change the identity of the tRNA (23). One speaks of alloacceptor or isoacceptor remolding, respectively, depending on whether the accepted amino acid is changed or not. In addition, we consider remoldings between the two isoacceptor tRNAs of leucine and serine as alloacceptor remoldings. Alloacceptor remolding is thought to be caused by a duplication-loss mechanism (Figure 1) that comprises three steps: First, one tRNA is duplicated, then the anticodon of one of the copies is mutated in such a way that it takes the identity of a second tRNA, and finally the original copy of the second tRNA is lost (23,24). Remoldings of tRNAs are frequently observed within animal mitochondrial genomes (e.g. 24; see also below). This is due to the peculiarities of mitochondrial genetics:

- Only a reduced set of identity elements for the aminoacylation of mitochondrial tRNAs exists, i.e. for most tRNAs the anticodon is the main identity element (15,21).
- The simplification of the translation machinery has left only a single tRNA for each amino acid (except for *trnL* and *trnS*). This is compensated by the possibility of wobble base pairing (12) and codon bias.
- Tandem duplication random loss is a major mechanism of genome rearrangement in metazoan mitogenomes (10,25), whence duplication and loss events are probably not at all infrequent.

The feasibility of the above mechanism for alloacceptor remolding has been confirmed experimentally in *E. coli* (26). For genetic systems that have tRNA identity elements beyond the anticodon it was shown that the identity of a tRNA can be switched by corresponding substitutions outside of the anticodon (27). In (12) an alternative remolding mechanism has been suggested whereby a defunct tRNA is replaced by a mutated copy of another tRNA.

Previous studies presented multiple cases of tRNA remoldings within various metazoan mitochondrial genomes, e.g. multiple remoldings of *trnL(CUN)* to *trnL(UUR)* during metazoan evolution (24,28). Remoldings of tRNAs in demosponge mitogenomes have been well documented (29–31). Furthermore anecdotal evidence of tRNA remoldings in mitogenomes is available for most major animal taxa: Mollusca (32,33), Arthropoda (34,35), Tunicates (36), Echinoderms (23) and Chordata (37). The remolding of tRNAs is not limited to metazoan mitogenomes but has been reported also for fungal mitogenomes (38); nuclear genomes of mouse (39), primates (31), cow (40), fruit fly (41), nematodes (42) and yeast (43); as well as for bacterial genomes (43). Other studies related tRNA anticodon mutations to diseases (44,45). Nevertheless, a comprehensive survey is still lacking.

Related mechanisms by which a single tRNA can functionally replace two tRNAs are RNA editing, e.g. in marsupial mitogenomes (46), and heteroplasmic variants, e.g. in isopod mitogenomes (47). In the former case post-transcriptional modifications lead effectively to a remolding of a fraction of the tRNA transcripts so that both anticodon variants are present simultaneously. In the latter case both variants are present as polymorphism in the population of mitochondrial DNA molecules.

The detection of tRNA remolding events is important for several reasons. The available methods for tRNA prediction, i.e. tRNAscan-SE (48), tRNAfinder (49), ARAGORN (50), ARWEN (51) and MiTFi (10), assign the tRNA identity solely based on the anticodon. This rule affects the available tools for automatic mitogenome annotation. Remolding events interfere with annotation since function no longer follows sequence homology in this case. This causes manifold problems with downstream analyses, e.g. false positive calls of rearrangement events in gene order analyses and incorrect phylogenetic reconstructions based on tRNAs. Furthermore, since tRNAs are a central component of mitochondrial gene translation, the knowledge about tRNA remolding events is important for a detailed understanding of the evolution of variant genetic codes as well as bias of the nucleotide or amino acid composition in mitogenomes.

Currently there are two main approaches for the detection of tRNA remolding events.

- The joint phylogenetic analysis of remolded and unremolded tRNAs can be used to reveal the true phylogenetic relationship, in particular the monophyly of the original and the remolded tRNAs in the descendants of a remolding event (see Figure 1B). The standard methods of phylogeny reconstruction have been used for this purpose, including Markov chain Monte Carlo (28), Maximum likelihood (24,38), Maximum Parsimony (24), Neighbour-joining (29,31,33) and Bayesian analyses (24,38). The small number of positions of a tRNA, however, severely limits the phylogenetic resolution. These methods thus have been applied either to phylogenetically relatively small data sets (e.g. 29) or to a small selection of a wide range of taxa (e.g. 28). Hence, these methods seem to be impracticable for a comprehensive large-scale search for remolding events. Furthermore, the large dissimilarity between different alloacceptor tRNAs might render these methods vulnerable to long branch attraction.
- The second approach uses pairwise similarity of the tRNAs directly (52,53). The basic idea is to search for tRNAs with a high similarity to an alloacceptor tRNA of the same species and low similarity to the isoacceptor tRNA in a closely related species. This has been applied to the mitogenomes of demosponges using the sequence identity in a pairwise sequence alignment as measure (29,31). While this approach avoids potential problems of phylogeny reconstruction, it has the disadvantage that it does not yield a phylogenetic interpretation for ancestral events.

In this paper we present a comprehensive analysis of alloacceptor tRNA remolding in metazoan mitogenomes that is based on extended and novel methods to detect and analyze tRNA remolding in large data sets:

- similarity-based remolding detection (SRD),
- maximum likelihood remolding detection (MLRD) and
- customized annotations of multiple sequence alignments.

## MATERIALS AND METHODS

### Similarity based selection of remolding candidates

This section describes a similarity based method for the selection of remolding candidates (SRD). Let *X*_*i*_ denote a *trnX* in the mitogenome of species *i* and let *S*(*P*|*X*_*i*_) be the bitscore of the structural alignment of the set of tRNAs *P* based on the covariance model of *trnX* in species *i*. For one element sets {*Y*} we simply write *S*(*Y*|*X*_*i*_) instead of *S*({*Y*}|*X*_*i*_). Structural alignments and bitscores are computed using the cmalign program included in the Infernal package version 1.1 (54). See Supplement 1.1 for an explanation of structural RNA models. The bitscore distributions obtained for equal and unequal tRNA pairs are well separated, see Supplement 1.2. Therefore bitscores can be used directly to distinguish orthologous and non-orthologous tRNAs.

A set 𝒫 of putative cases of tRNA remolding is determined as exceptionally similar pairs of alloacceptor tRNAs contained in the same mitogenome. For each species *i* and each tRNA *X*_*i*_ of the mitogenome of *i*, the pair of tRNAs {*X*_*i*_, *Y*_*i*_} with *X* ≠ *Y* is included in the list 𝒫 of candidate remoldings whenever *S*({*X*_*i*_, *Y*_*i*_}|*X*_*i*_) is a significant (*P* < 0.05) outlier in the distribution of the bitscores *S*({*X*_*i*_, *Z*_*i*_}|*X*_*i*_) for all tRNAs *Z*_*i*_ with *X* ≠ *Z*. For each tRNA *X*_*i*_ of a species *i* only the pair {*X*_*i*_, *Y*_*i*_} with the maximum score *S*({*X*_*i*_, *Y*_*i*_}|*X*_*i*_) is checked to be an outlier. This is done by using the Grubbs test as implemented in the outliers (version 0.14) package of R (55) (version 3.1.1). It tests if the difference of the maximum value and the mean is larger than the difference of the mean and the minimum value of the distribution, and computes a corresponding *P*-value. Note that at this point no information on the direction of the remolding has been derived.

In addition to the remolding candidate list 𝒫 we construct a set 𝒩 of outliers that are not necessarily significant. This is done analogous to the construction of 𝒫 but no *P*-value threshold is applied. In a slight abuse of notation we write *X*_*i*_ ∉ 𝒩 if no pair {*X*_*i*_, *Y*_*i*_} exists in 𝒩 for *Y* ≠ *X*, i.e. if *X*_*i*_ is not involved in any remolding.

In the second step each remolding candidate {*X*_*i*_, *Y*_*i*_} ∈ 𝒫 is examined with the following procedure for both *X*_*i*_↝*Y*_*i*_ and *Y*_*i*_↝*X*_*i*_. Here *X*_*i*_↝*Y*_*i*_, with *X* ≠ *Y*, denote that *Y*_*i*_ derives from the remolding of *trnX* into *trnY* in the lineage leading to species *i*. We call *trnX* and *trnY* the (remolding) *donor* and *acceptor*, respectively. Let *j* = *C*_*XY*_(*i*) denote a species with the smallest patristic distance to *i* for which *X*_*j*_, *Y*_*j*_ ∉ 𝒩 holds, i.e. neither *X*_*j*_ nor *Y*_*j*_ is likely to have been involved in any remolding event. For *X*_*i*_↝*Y*_*i*_ to hold the following criteria must be fulfilled:

- In order to establish that *Y*_*i*_ and *X*_*j*_, with *j* = *C*_*XY*_(*i*), are unlikely to be different alloacceptor tRNAs we require that *Y*_*i*_ is significantly more similar to *X*_*j*_ than one would expect for different tRNAs of the same species.
- In addition, it is assured that *Y*_*i*_ and *X*_*j*_ are homologs by demanding that they are at least as similar as pairs of unremolded *trnX* sequences in closely related species.
- In order to obtain the remolding direction we use the fact that in the case of a *X*_*i*_↝*Y*_*i*_ *Y*_*i*_ is phylogenetically closer related to *X*_*j*_ than to *Y*_*j*_. This should be reflected in a higher level of sequence similarity between *Y*_*i*_ and *X*_*j*_ than between *Y*_*i*_ and *Y*_*j*_ (see Figure 1B). In the case of the reverse remolding direction we expect that *X*_*i*_ and *Y*_*j*_ are more similar than *X*_*i*_ and *X*_*j*_.

More formally the criteria for *X*_*i*_↝*Y*_*i*_ are

- *S*(*Y*_*i*_|*X*_*j*_) ⊐ {*S*(*Z*_*k*_|*X*_*k*_): *Z* ≠ *X*, *Z*_*k*_, *X*_*k*_ ∉ 𝒩},
- *S*(*Y*_*i*_|*X*_*j*_) 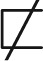 {*S*(*X*_*l*_|*X*_*k*_): *k* = *C*_*XY*_(*l*)} and
- *S*(*Y*_*i*_|*X*_*j*_) > *S*(*Y*_*i*_|*Y*_*j*_),

with *j* = *C*_*XY*_(*i*), where the comparison for the first two criteria is implemented as a non-parametric significance test, here the Wilcoxon signed-rank test (*P* ≤ 0.05), where the alternative hypothesis is indicated by the comparison operator (⊐ ‘more similar’ and 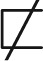 ‘not less similar’). We denote with ℛ the set of remolding candidates from 𝒫 that pass all three criteria. Note that in the second criterion, the test has been performed so that the similarity is smaller than for the same tRNAs from closely related species (*P* > 0.05).

### Maximum likelihood based test for remolding

We propose an efficient new method, called MLRD, to identify the position of a remolding event within a given phylogenetic tree. Consider a remolding event *X*_i_↝*Y_i_* on the edge leading to a subtree *i* and let *j* be the sister subtree of *i* (see Figure 1A). Because the *trnY*s in subtree *i* are actually homologous to *trnX* a phylogenetic reconstruction of the set of *trnX* and *trnY* of the two subtrees should produce a tree as depicted in Figure 1B. The *trnY* of subtree *i* are closer related to the *trnX* of subtree *i* than to the *trnX* of subtree *j* since the speciation of *i* and *j* happened prior to the remolding event. Clearly, the *trnY* of subtree *j* are basal to the other tRNAs and are connected via the longest branches in the tree since they only have in common that both are tRNAs (i.e. probably an extremely distant common ancestor). This is in contrast to the reconstruction of a remolding free data set where the *trnX* and the *trnY*, respectively, are expected to form monophyletic groups which are connected by long edges. A phylogenetic evaluation of these two alternatives will prefer the tree depicted in Figure 1B.

The idea stated above has been used previously for detecting tRNA remolding (24,28,31). However, if a reasonable approximation of the species tree is known *a priori*, it is not necessary to reconstruct the entire gene phylogeny from scratch. Instead, it suffices to compare and evaluate the tree topologies predicted for a remolding event at each particular edge of the species phylogeny.

**Figure 2.**
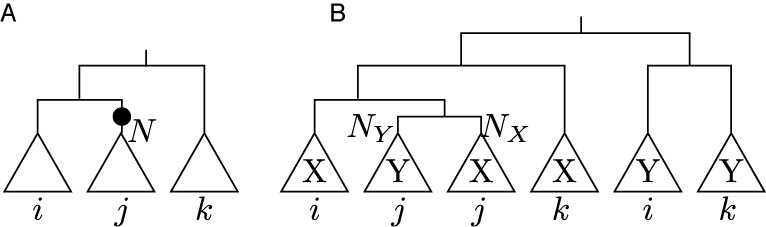
(**A**) Species tree including three subtrees *i*, *j* and *k* that is to be tested for an X↝Y on the edge leading to node N (marked with a circle); (**B**) The corresponding gene tree for *trnX* and *trnY* that is used for testing for the remolding event.

Therefore we create a phylogenetic tree consisting of two identical copies of the species tree below a common root node. Each of the two subtrees describes the gene phylogeny of one of the two tRNAs in question in the absence of remolding. For each node *N*_*Y*_ in the acceptor subtree, i.e. *trnY*, we create a modified phylogeny by moving the subtree below this node to the donor subtree, i.e. *trnX*, such that *N*_*Y*_ becomes the sister node of the corresponding node *N*_*X*_. In the resulting tree *N*_*X*_ and *N*_*Y*_ are the children of a new node which is the child of the parent of *N*_*X*_ in the original tree, see Figure 2. Each of these tree topologies corresponds to the topology expected for a X↝Y-remolding event taking place on the edge leading to *N*. These tree topologies can be evaluated in terms of their log-likelihood values given the tRNA sequences. We employed RAxML version 8.0.25 (56) with the -f N option for computing the log-likelihood values. The computation is based on the combined alignment of the tRNAs using the model of the donor as described above. High log-likelihood values, in particular values greater than those obtained for the unremolded original topology, indicate remolding events on the edges leading to the subtree that was moved.

### Data set

We used all available complete metazoan mitogenome sequences from RefSeq release 63 (57) and re-annotations obtained with an optimized version of MITOS (58) which yields structural annotations. A bifurcated phylogeny for the Metazoa contained in the RefSeq data has been obtained from the NCBI taxonomy database (59) using the state of 06-Mar-2014. A more detailed analysis has been conducted with MLRD and alignments for sets of remolding events with identical pairs of donor and acceptor anticodons. The data sets contain the two tRNAs that are involved in the remolding for all species in the subtree of interest and two automatically selected outgroup species. The only exception is the metazoan leucine data set for which only a subset of the metazoan species was selected. A detailed description of the methods is given in Supplement 1.3.

We conducted systematic literature research much beyond the well-known first examples (23,60) and the subsequent more systematic analyses of tRNA remolding (24,28,29,31). We retrieved the PubMed entries associated with RefSeq genbank record for all taxa included in the present study and scanned the publications for the keywords ‘remolding’, ‘recruitment’ and ‘tRNA’.

## RESULTS AND DISCUSSION

In the following we present a detailed analysis of the events in the remolding candidates set ℛ that has been determined with SRD as described in the Methods section. Since we have tuned our criteria for specificity we have to expect a sizeable number of false negatives. With the help of MLRD and sequence alignments we therefore checked whether the same type of remolding event was observed also in the phylogenetic vicinity of remolding candidates identified by SRD. This allows us to determine whether the remolding event occurred already in an earlier ancestor and was then preserved throughout a larger group of related taxa. In this case multiple remolding candidates actually point to a single remolding event. We start the presentation of our results by first providing an overview of the candidates that were detected.

### General results

#### Overview

**Figure 3.**
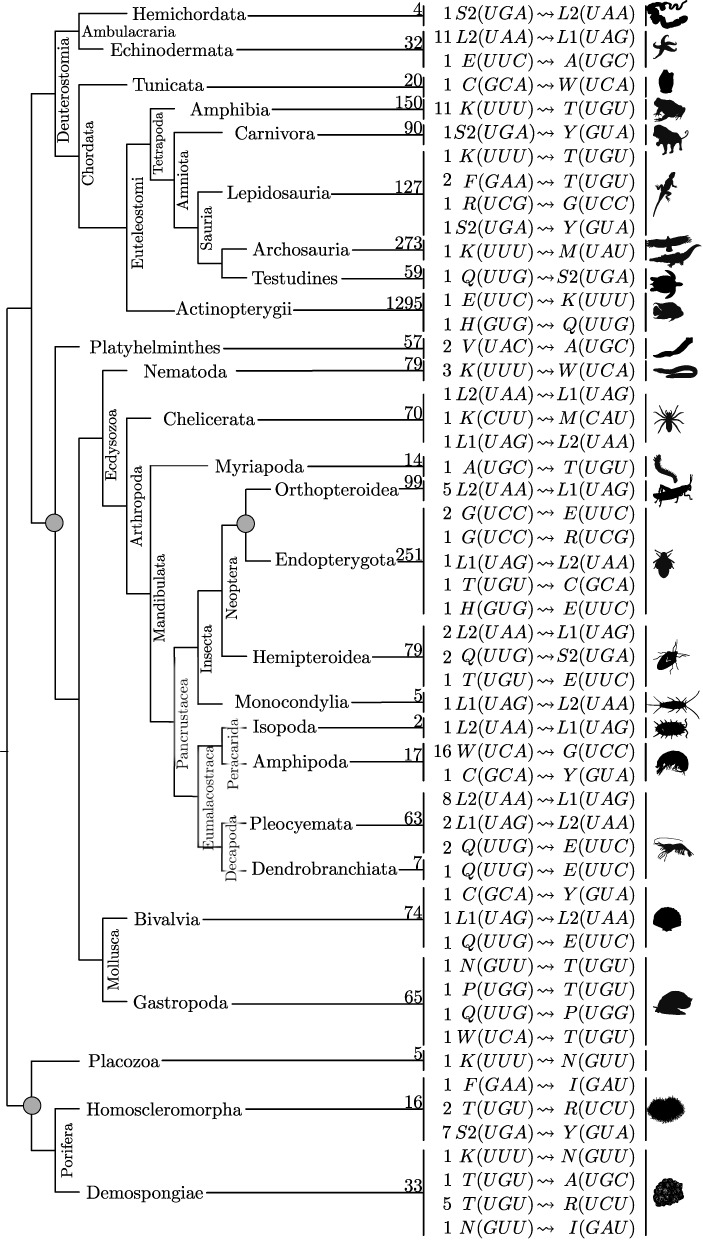
Detected tRNA remolding pairs. For each leaf the remoldings and their number is shown. Taxa without remoldings are omitted. Gray circles mark nodes that are not present in the NCBI taxonomy. Homoscleromorpha and Demospongiae have been separated for clarity.

We applied our method for detecting remolding candidates to 3817 metazoan mitogenomes. This resulted in a set 𝒫 consisting of 6601 pairs of alloacceptor tRNAs of the same species that are exceptionally similar, i.e. putative cases of tRNA remolding, see Supplement 2. After the application of the three remolding criteria this set was reduced to the set ℛ containing 118 putative remolding cases, see Supplement 3. The set ℛ contains remolding candidates in most phyla (Figure 3). Only *trnS1* and *trnD* were never included in remolding events. Note that *trnS1* commonly lost the D-domain in Metazoa.

Elements from 𝒫 are not included in ℛ because in most cases (70.6%) all three criteria were violated, often only two criteria were not fulfilled, i.e. i)+ii) and ii)+iii) in 3.4% and 21.0%, respectively. Virtually never only a single criterion was responsible for the removal.

The number of times the second and third position of the anticodon is affected by the unique remoldings in ℛ is nearly identical, i.e. 19 and 18, see Supplement 4. Since only alloacceptor remoldings are considered the 1st position is affected less often (8). In more than 10 cases each, the remolding switched tRNAs within different two-codon families (12), from a two- to a four-codon family (14), or the other way around (11). Considering the changed nucleotides A↔C appears least frequent (1 AC, 0 CA) and G↔U most frequent (8 in each direction). Interestingly, G↔U and U↔G happens only at the first position. Based on our data there is no obvious relation between remolding and nucleotide bias.

In many cases both, acceptor and the donor tRNA, are located near each other on the mitogenome, i.e. 24.6% <100*nt* and 39% <1000*nt*. About 32% are separated by a single gene or less, see Supplement 5. K↝T is a prominent counter example since *trnK* and *trnT* are separated by 15 genes. Furthermore in 72% of the cases donor and acceptor tRNA are on the same strand. The conservation of the strandedness and the small distance is in agreement with a local duplication mechanism as cause of tRNA remolding.

To decide if tRNA remolding is caused by a deletion-duplication or duplication-deletion process we checked for each tRNA remolding in ℛ if the taxa contained in the subtree rooted at the sister or the sister of the parent remained in the pre-duplication state, i.e. with a deletion of the acceptor, or in the pre-deletion state, i.e. with a duplicated donor. No example was detected where the complete sister subtree was in either state, see Supplement 6. Hence, none of the mechanisms is clearly supported.

#### Post remolding adaption

In the presence of tRNA identity elements other than the anticodon one can expect side effects due to occasional misaminoacylation of the remolded tRNAs which is caused by a mis-matching set of identity elements, i.e. an influence of the codon frequencies or the adaption of the additional identity elements.

In order to estimate the influence of tRNA remolding on the codon frequencies we calculated the normalized differences of the frequencies of the codon(s) affected by the tRNA and the corresponding codon(s) in a closely related species where the tRNA is not remolded. The analysis has been performed separately for the precise codon complementary to the anticodon of the tRNAs and for the entire codon box to account for the degeneracy of the third codon position, see Supplement 7. For both, codons and codon box frequencies, the differences between remolded and unremolded examples tend toward both extremes. For the tRNAs in ℛ with increased or decreased codon frequencies, 36% and 34%, respectively, are outside of the 5% or 95% quantiles of the corresponding distribution for unremolded tRNAs. The null hypothesis that the differences in codon usage for tRNA pairs in ℛ are not less than codon usage differences for tRNA pairs in 𝒩 has to be be rejected (*P* < 0.01) for

- the codon frequencies of the remolding acceptor and
- the codon box frequencies of the donor.

That is, after a remolding both the frequency of the codon corresponding to the acceptor anticodon and the codon box frequency corresponding to the donor amino acid decreases significantly more than for cases without remolding. This might be explained by insufficient adaption of minor identity elements which may result in misaminoacylation of the acceptor, i.e. the acceptor tRNA is still occasionally loaded with the donor amino acid and thus results in a reduced functionality of proteins using these amino acids. Alternatively, the reduced codon frequency might have facilitated the remolding. Interestingly the extreme values of the differences are reduced and the distributions do not differ significantly when all but one randomly chosen representative of tRNA remolding pairs with the same donor and acceptor combination are removed. Note that many of the removed pairs of remolded tRNAs stem from ancestral events. Therefore the remaining data contain a larger fraction of recent remolding events. That is, for the corresponding mitogenomes less time passed after the remolding which might explain a weaker adaption of the codon frequencies. Since remoldings influence the codon frequencies they might play a role in codon reassignment.

The remolded tRNAs might be affected by a deleterious period during the coexistence of the redundant tRNAs or post remolding adaptations due to selection. Note that in the few known cases where duplicate tRNAs are found an accumulation of mutations can be observed for one of the two tRNAs (10). Hence, the loss can be assumed to be instantaneous. The influence of tRNA remolding on the remolding acceptor itself was analyzed as follows, see Supplement 8. Assume a D↝A ancestral to a set of species *X* and let *Y* be a set of closely related species of the same size that was not subject to a remolding involving *trnD* or *trnA*. In *Y* the two tRNAs are in ‘ancestral’ state, or more precisely, they have not been subject to remolding related changes. Furthermore, let *trnU* be a tRNA that was not involved in a remolding in *X* and *Y*. Two alignments with tRNA from *X* and *Y* have been constructed:

- *trnA* from *X* and *trnD* from *Y* and
- *trnU* from *X* and *Y*.

**Figure 4.**
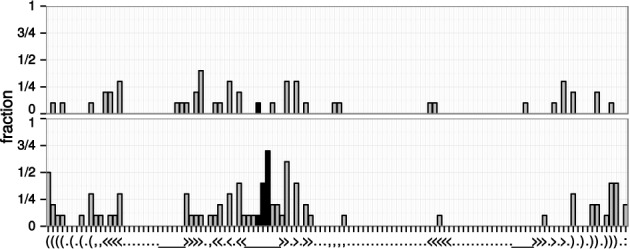
Fraction of the 10 analyzed data sets that show tRNA specific modification in an alignment column for the control (top) and remolded tRNAs (bottom). The consensus secondary structure is shown along the plot. The anticodon positions are highlighted by black bars.

For each alignment column it is determined whether the tRNAs from *X* have more than 0.5 Bit for one nucleotide and the tRNAs from *Y* have more than 0.5 Bit for another nucleotide. This has been conducted for 10 of the larger data sets that have been analyzed in detail in this study, see Figure 4.

Only three positions are modified in a tRNA specific way in at least 50% of the data sets. One of them is an anticodon position while the other two are the first positions in the acceptor stem, and another paired nucleotide in the anticodon stem. In general tRNA specific changes are found nearly exclusively in the acceptor, D- and anticodon stem. In comparison the unremolded control data set shows less group specific modifications, in particular no changes are found in more than 50% of the data sets. This indicates that the acceptor and the anticodon stem contain minor identity elements which are modified due to selection for a corresponding set of identity elements. These findings coincide with the known identity elements of tRNAs, i.e. in the acceptor stem and anticodon arm (21). Due to the small sample of analyzed tRNAs and the diverse set of tRNAs that are jointly analyzed, these findings need further verification.

In the following we discuss remolding candidates in Eumalacostraca and Porifera in detail. Furthermore, an overview of the findings on the leucine remoldings is discussed. We give a detailed overview of other taxa with prominent remolding candidates which is detailed in the supplement.

### Eumalacostraca

**Figure 5.**
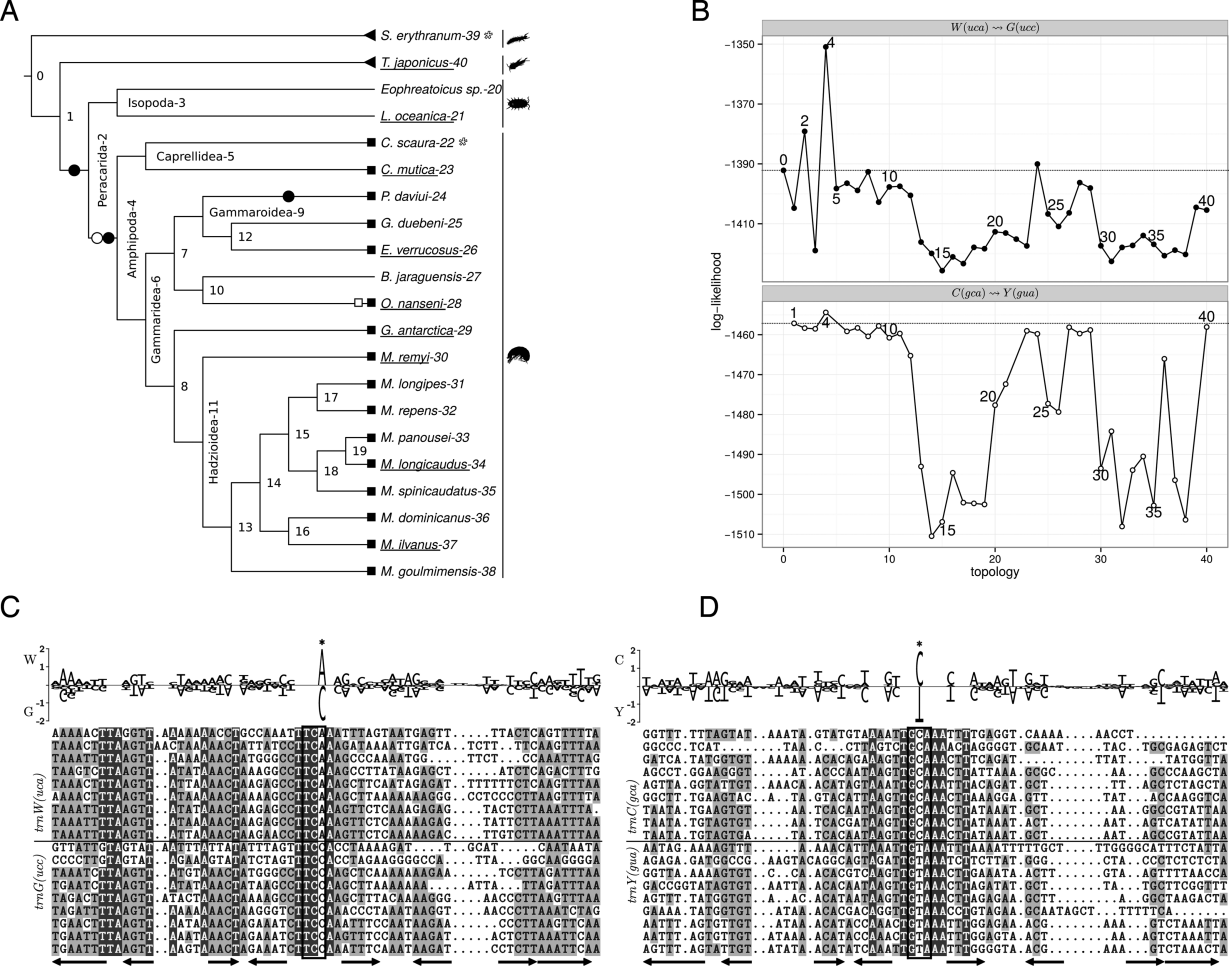
Remolding of W↝G and C↝Y in Peracarida: (**A**) guide tree, black and white symbols refer to the W↝G and C↝Y, respectively, sequences of underlined species are shown in the alignment, triangles mark outgroup species, squares mark candidates in ℛ, circles mark tree rearrangements which yield a higher log-likelihood score compared to the start tree, and stars mark species that are omitted due to missing annotations; (**B**) log-likelihood values calculated for each tree topology, numbers refer to the root node of the subtree that was moved; (**C**) and (**D**) alignment and subfamily logo for the *trnW*+*trnG* and *trnC*+*trnY*, respectively, only the species underlined in panel (A) are included in the alignment shown here. Arrows mark secondary structure elements: the stems of the acceptor arm, D arm, anticodon arm and T arm. The anticodon is indicated by a box.

The Eumalacostraca exhibit multiple clear examples for tRNA remolding, Figure 5. The candidate set ℛ contains three types of remolding events for the superorder Peracarida: 16 W(UCA)↝G(UCC) for all Amphipoda except of *Bahadzia jaraguensis* [#27] ( [#X] refers to the node number X within the corresponding tree), 1 C(GCA)↝Y(GUA) for *Onisimus nanseni* [#28], and 1 L2(UAA)↝L1(UAG) for *Eophreatoicus sp*. [#20]. Due to the coincidence of these remolding types in a rather narrow taxonomic group we have analyzed all of them in more detail.

#### Remolding of W↝G in Eumalacostraca

Our results for the W(UCA)↝G(UCC) remoldings (Figure 5) are in complete agreement with (35). Based on the high sequence similarity of the two tRNAs in *Caprella mutica* [#23] and the conserved gene adjacency of the two tRNAs in all amphipods these authors suggested a remolding event at the ancestor of the Amphipoda. The W(UCA)↝G(UCC) remolding in all amphipods (including *B. jaraguensis* [#27], which was not in the candidate set) is evident from the alignment and subfamily logo, Figure 5C and Supplement 9.1, which shows the modified anticodon position as the only significant difference between *trnW* and the *trnG*. Consistent with the remolding at the root of the Amphipoda the alignment also shows that the *trnG* of the Amphipoda are highly similar to the donor (*trnW*) of the Peracarida and even to the *trnW* of the hexapod that was used as outgroup, while the *trnG* of the Isopoda and the outgroup species are clearly a different tRNA. Only for the tree topologies supporting W(UCA)↝G(UCC) at the root of the Amphipoda and the root of the Peracarida significantly increased log-likelihood values are found (compared to the log-likelihood of the topology that assumes no remolding), see Supplement 9.1 and Figure 5B. A distinct maximum is found for the Amphipoda. The increased log-likelihood value for the Peracarida might be a consequence of the small sample size of the Isopoda so that the Amphipoda dominate the log-likelihood computation for the Peracarida. Further evidence for this remolding event comes from the gene orders. *trnG* is found in a well preserved large gene cluster between *cox3* and *nad3* in 86.8% of the arthropods including Decapoda and Isopoda. In Amphipoda *trnW* and *trnG* are adjacent within the tRNA cluster which is consistent with a (tandem) duplication plus anticodon mutation of the ancestral *trnW* and loss of the *trnG*, see also (61).

#### Remolding of C↝Y in Eumalacostraca

Although the candidate set contains only one C(GCA)↝Y(GUA) remolding in the Peracarida, our data support an additional ancestral remolding event at the root of Amphipoda. To the best of our knowledge this remolding event has not been described in the literature before. We observe (Figure 5A and B) a single slightly increased log-likelihood value for a remolding event at the ancestor of the Amphipoda. The alignment of the tRNA sequences shows multiple, well preserved columns in stem regions, in particular within the anticodon stem. The third position of the anticodon is the only notable specific difference between the two tRNAs, see Figure 5D. The alignment also shows that the *trnY* of the Amphipoda are highly similar to the donor (*trnC*) of the Peracarida (except Isopoda) and the two Decapoda species, see also Supplement 9.2. However, the *trnY* of the Decapoda and Isopoda are clearly different tRNAs. In order to assess the observed similarity of the *trnC* and the *trnY* we compared the alignment to an alignment of *trnC* with another randomly chosen tRNA, i.e. *trnF*, for the same species, and an alignment for *trnC* and the *trnY* of a related group, i.e. Hymenoptera, see Supplement 9.3 and 9.4. In contrast to the *trnC*-*trnY* alignment for the Peracarida both alignments showed pronounced tRNA specific differences and the corresponding results of MLRD showed no increased log-likelihood values. The *trnC*, *trnY* and *trnW* are in close proximity in many arthropod mitogenomes, i.e. the configuration *nad2, trnW, trnC, trnY, cox1* is found for 71.2% of the Arthropoda. In Peracarida the *nad2* is located between *trnW* *trnG* and *trnC* *trnY*. This might indicate a common origin of the C(GCA)↝Y(GUA) and W(UCA)↝G(UCC) remoldings in the wake of a single duplication event that affected at least *nad2*, *trnW* and *trnC*. Using TreeREx (62) it was not possible to obtain a reliable unambiguous reconstruction of the rearrangements for the root of the Amphipoda with the available species and phylogeny.

Since our outgroup selection method rejected the Decapoda as outgroup we have run a separate analysis including the Decapoda. The results clearly support the root of the Amphipoda as the most plausible position of both remolding events, see Supplement 9.5.

#### Other remoldings in Eumalacostraca

A Q(UUG)↝E(UUC) remolding was detected in the mitogenomes of *Macrobrachium nipponense*, *Nautilocaris saintlaurentae* (Caridea) and *Marsupenaeus japonicus* (Penaeoidea). A detailed discussion of this remolding event can be found in Supplement 9.6. The leucine remoldings in the Eumalacostraca are discussed separately in the last section.

### Porifera

The mitogenomes of Porifera are often rearranged, and duplicated tRNAs are present, i.e. *trnR(UCG)* and *trnR(UCU)*. Two to three copies of *trnM(CAU)* (where one is post-transcriptionally edited to a copy of a *trnI*) are found throughout Porifera, while Homoscleromorpha exhibit additional copies of *trnT(UGU)* and *trnV(UAC)*, see (63). Seemingly independent tRNA remolding events have been described for 7 out of 21 species in a rather systematic analysis (31) which probably describes the mentioned ‘several additional instances of tRNA gene recruitment in demosponge [mitochondrial DNA] (to be described elsewhere)’ mentioned in (64). By using our new methods we extend the number of tRNA remoldings known for Porifera considerably and show that many of them are not independent events.

The candidate set ℛ contains multiple instances of S2(UGA)↝Y(GUA) and T(UGU)↝R(UCU) which are discussed together with other remoldings that are known from the literature in the following.

#### Remolding of T↝R in Porifera

**Figure 6.**
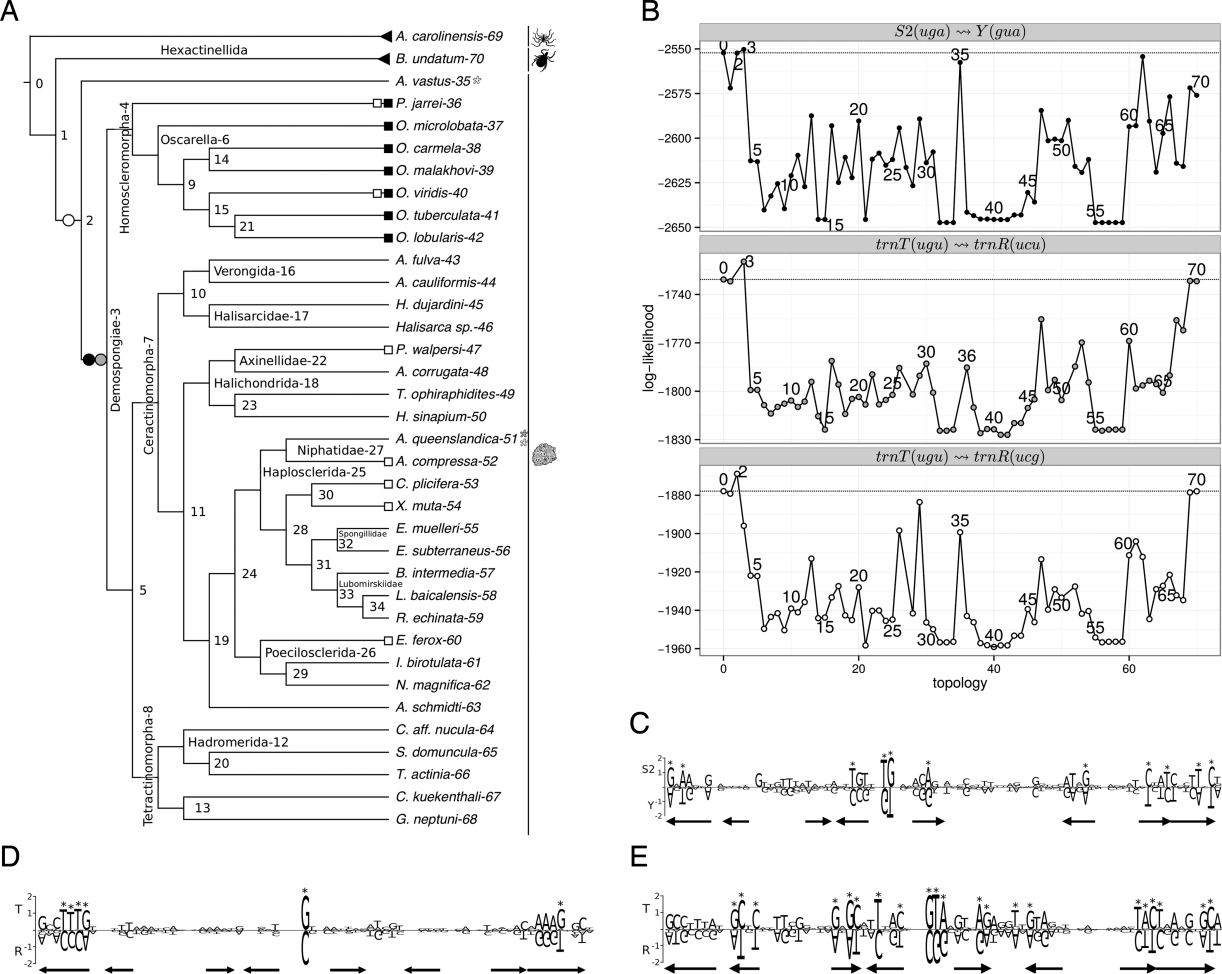
Results for the Porifera T(UGU)↝R(UCU), T(UGU)↝R(UCG) and S2(UGA)↝Y(GUA): (**A**) guide tree, black, gray and white symbols refer to S2(UGA)↝Y(GUA), T(UGU)↝R(UCU) and T(UGU)↝R(UCG), respectively, triangles mark outgroup species, squares mark candidates in ℛ, circles mark tree rearrangements which yield a higher log-likelihood score compared to the start tree, and stars mark species that are omitted due to missing annotations; (**B**) log-likelihood score calculated for each tree topology, numbers refer to the root node of the subtree that was moved; (**C**), (**D**) and (**E**) subfamily logo for the *trnS2(UGA)*+*trnY(GUA)*, *trnT(UGU)*+*trnR(UCU)* and *trnT(UGU)*+*trnR(UCG)*, respectively, genes of the Porifera and outgroup species. Arrows mark secondary structure elements: the stems of the acceptor arm, D arm, anticodon arm and T arm.

Despite that all seven candidates were T(UGU)↝R(UCU) remoldings, we have examined two separate data sets, i.e. one for each copy of *trnR*, see Figure 6; and Supplement 10.2 and 10.3. The MLRD analysis showed a single increased log-likelihood value corresponding to a T(UGU)↝R(UCU) at the root of the Demospongiae and a T(UGU)↝R(UCG) remolding at the root of Porifera. The alignment of *trnT(UGU)* and *trnR(UCU)* shows a nearly perfect conservation except for the modified anticodon position and the acceptor stem, which are tRNA specific. In contrast to the alignment of *trnT(UGU)* and *trnR(UCG)* which shows a large number of differences that are specific for each tRNA. Both genes are involved in well preserved adjacencies in gene orders of Demospongiae, i.e. in the 47 available mitogenomes (i) 29 *cob* *trnT(UGU)* *trnS1*, (ii) 31 *atp6* *trnR(UCU)* *cox3* and (iii) 31 *nad3* *trnR(UCG)* *nad4l* configurations are found, where in few cases only one of the two adjacencies is preserved.

The adjacencies are absent in the single available Hexactinellida and the tRNAs are not found in the multichromosomal mitogenome of the one available Calcarea. Altogether, this suggests a T(UGU)↝R(UCU) remolding that is ancestral to all Porifera. Whether there was an independent T(UGU)↝R(UCG) remolding ancestral to Porifera cannot be decided without a more detailed evaluation. In particular, fungi need to be included as an outgroup to pinpoint the time of the remolding.

The positions of identity elements, and the charging/the progress of arginylation of the mitochondrial *trnR* genes of many Metazoa, in particular Porifera, has been analyzed in (14). A very important factor, the fact that *trnR* are remolded *trnT*, remained unnoticed within this study. For *Iotrochota birotulata* it was shown that only *trnR(UCU)* is charged (by insect arginyl-tRNA synthetase). The *trnR(UCG)* is not arginylated as the last position of its anticodon is a U, which was shown to be a negative determinant for the enzyme. The basal *Aphrocallistes vastus* showed no arginylation activity for both tRNAs. The high number of tRNA specific columns in the alignment of *trnT(UGU)* and *trnR(UCG)* could indicate adaptations due to selection that favors arginylation. The *trnR(UCG)* of *A. vastus* appears to be less modified. Unfortunately, no statement about the aminoacylation activity for *trnR(UCU)* is given.

Notable exceptions to the preserved adjacencies of *trnT(UGU)* and *trnR(UCU)* are *Ptilocaulis walpersi* [#47] where *trnR(UCU)* is located between *cob* and *trnS1* while *trnT(UGU)* is located between two different genes, i.e. *cox1* and *trnS2*, and *Ectyoplasia ferox* [#60], where *trnT(UGU)* is at its usual position. Here, however, *trnR(UCU)* is located between *cox1* and *trnS2*. An additional T(UGU)↝R(UCU) remolding for these two species was likely generated by a single duplication, see also (31). That is, *trnT(UGU)* is duplicated and placed between *cox1* and *trnS2*. Subsequently, one of the copies is remolded into *trnR(UCU)*: while in *Ptilocaulis wilhelmi* (our study includes *P. walpersi*) the original is remolded, in *E. ferox* the copy is remolded. Finally the redundant copies of *trnR(UCU)* between *atp6* and *cox3* are lost. The interpretation of the MLRD results for these two species is complicated. Both, *trnT(UGU*) and *trnR(UCU)*, were already highly similar prior to the two putative recent remoldings due to an ancestral T(UGU)↝R(UCU) remolding (given the high similarity of all *trnT(UGU)* and *trnR(UCU)* there is no reason to assume that *P. walpersi* and *E. ferox* were not affected by the ancestral event). The increase of the log-likelihood for the nodes when compared to their respective neighbors might be interpreted as a possible sign for the recent T(UGU)↝R(UCU) re-remolding. A larger sample of closely related species will be necessary to verify this hypothesis. The phylogenetic interpretation of these remoldings with respect to the NCBI taxonomy would suggest parallel identical duplications (such that the copy is inserted at the same place) or multiple independent losses. This suggests major problems of the NCBI taxonomy for the Porifera. In a reconstruction presented in (64) the two species are sister taxa. Analogous interpretation of the MLRD results for other nodes with increased log-likelihood would suggest further recent T(UGU)↝R(UCU) remolding events. However, the conserved adjacencies of the tRNAs are a strong counter-argument.

#### Remolding of S2↝Y in Porifera

The predicted S2(UGA)↝Y(GUA) remolding is one of the cases that requires the mutation of two positions of the anticodon. The candidate set ℛ contains 7 cases for Homoscleromorpha. The maximum likelihood analysis showed a slightly increased log-likelihood value for the Porifera (see Figure 6B). However, a slightly smaller log-likelihood value (compared to the topology assuming no remolding) is found for the Homoscleromorpha and the node representing Demospongiae plus Hexactinellida (which are represented only by *A. vastus* [#35]). Given the poorly resolved taxonomy of Porifera and several only insufficiently sampled taxa the results of the maximum likelihood method cannot be interpreted unequivocally for one of the alternatives.

An S2(UGA)↝Y(GUA) remolding that is ancestral to the Porifera is supported by both, the alignment and the gene orders. In the alignment nearly half of the columns (38/88) are well preserved for both tRNAs. They are found in particular in the acceptor-, D- and C-stem. Eleven (including the two anticodon mutations) columns with mutations specific for each of the two tRNAs exist. The loop regions are highly variable, especially the variable loop is highly divergent, while its size seems to be conserved, see Supplement 10.1. Both tRNAs are involved in well preserved adjacencies. With a few exceptions, *trnY* is adjacent to at least one of *trnI(GAU)* and *trnM(CAU)* (31/47); and *trnS2(UGA)* is adjacent to *cox1* (28/47). Hence, assuming more recent remolding(s) requires that the remolded duplicate ends up at the exact same position as the original which seems to be unlikely.

#### Other remoldings in Porifera

An additional R(UCG)↝Y(AUA) remolding has been suggested within the mitogenome of *Negombata magnifica* [#62] (30,31). Our results show that these two tRNAs are included as candidates within 𝒫 but did not pass the third criterion (the same holds for Y(AUA)↝R(UCG)) and were therefore not included into the final results. Nevertheless, two arguments support this event:

- the atypical position of *trnY* (not adjacent to a *trnI*) and
- the atypical anticodon (it is the only *trnY* in Porifera that uses the anticodon AUA).

Two remoldings for *Axinella corrugata* [#48], K(UUU)↝N(GUU) and K(UUU)↝C(GCA), were predicted by (31). The pair *trnK*, *trnN* was included into our remolding candidates ℛ. The MLRD method for these two remoldings showed the maximum log-likelihood value for *A. corrugata*, see Supplement 10.4 and 10.5. Since the three tRNAs are adjacent in the mitogenome, a tandem triplication, or two duplications of *trnK* and alternative remolding of the copies can explain these remoldings. A further increased value is found for a K↝C remolding in *Agelas schmidti* [#63] where only *trnK* and *trnN* are adjacent. Again, these remolding events cannot be explained easily using the NCBI taxonomy but fit naturally if the two species are sisters, e.g. (64).

It has been suggested that ‘within the Oscarella-like genomes, two duplicated tRNA genes (*trnV* and *trnT*) have changed identities in some species’ without providing evidence (65). No *trnV*, *trnT* pair from Porifera was included in our set 𝒫. This could be an artifact caused by the restriction of our outlier tests to a single outlier, in this case the more similar *trnR*-*trnV* and *trnV*-*trnK* pairs. Hence, our analysis does not contradict the claim of (65). Indeed, a copy of *trnV* is found adjacent to one of the *trnT*, e.g. in *Oscarella carmela* [#38] which was analyzed before by (31), who found no remolding of this type. An MLRD analysis for V↝T and T↝V for the adjacent *trnT* and *trnV* genes is in agreement with (31) where no remolding could be detected in *O. carmela* since its likelihood is significantly decreased. However, the alignment (Supplement 10.6 and 10.7) shows a number of conserved columns (but also group specific columns). A slight increase in the likelihood could be observed at the root of the Porifera which might indicate a possible remolding.

For *A. schmidti* an N↝I remolding candidate was included in ℛ, whereas (31) suggested an I↝N remolding with a sequence similarity of 73.6%. Additionally, we detect an F↝I remolding in *Oscarella microlobata* [#37].

The leucine remoldings within the Porifera are discussed separately in the next section.

### Frequent remolding of leucine tRNAs

The remolding of the leucine tRNAs in Metazoa is certainly the best studied example. First reports noting the similarity of these tRNAs in a sea urchin (23) and mouse (60) already hypothesized the tRNA remolding mechanism. Later leucine remoldings were studied systematically in (24,28) for a small selections of taxa using rather weakly supported phylogenies. In the following the results for the leucine remoldings for Eumalacostraca, Ambulacraria, Mollusca and Metazoa are briefly discussed.

The analysis of the Eumalacostraca leucine remoldings highlighted this taxon as hotspot, see Supplement 11.1. The MLRD analysis revealed several nodes with clearly increased log-likelihood indicating parallel remolding events, i.e.

1. the Isopoda and *Eophreatoicus sp*. see (34),
2. Thalassinidea and many of its descendants see (66),
3. Paguroidea ((24) suggested a remolding for Anomura),
4. *Stenopus hispidus* see (67) and
5. *Euphausia pacifica*.

Parallel remolding events are also supported by gene orders. Furthermore, in the MLRD analysis for L1(UAG)↝L2(UAA) the highest peak is *G. dehaani* (68).

The L2(UAA)↝L1(UAG) remolding ancestral to the Ambulacraria as in (28) is supported by our data but independent events for Hemichordata and Xenoturbellida are possible (Supplement 11.2). The alignment shows that the leucine tRNAs of Ambulacraria are highly similar. However, SRD predicted remoldings only for Echinodermata and not for Hemichordata. In the MLRD analysis the log-likelihood is maximal for Ambulacraria, but increased values are also found for hemichordates and *Xenoturbella bocki*. The leucine genes are frequently adjacent in all Ambulacraria, but they are found between different genes in echinoderm, hemichordate and xenoturbellid mitogenomes.

Only a single leucine remolding candidate is predicted by SRD for Mollusca, but the set 𝒫 contains L1↝L2 pairs for 61.4% of the mollusc mitogenomes and an L2↝L1 for all of them. These cases might be false negatives of SRD since the alignment shows that leucine tRNAs are highly similar and an MLRD analysis for L2(UAA)↝L1(UAG) and L1(UAG)↝L2(UAA) remoldings in Mollusca showed multiple increased log-likelihood values (Supplement 11.3). In all major mollusc groups there are mitogenomes where the leucine tRNAs are adjacent and embedded between the same genes. Some of these cases agree with the results of the MLRD analysis. Hence, our data support an ancestral L1(UAG)↝L2(UAA) and suggest more recent leucine remoldings. This is in agreement with (24,28).

The MLRD analyses for L2(UAA)↝L1(UAG) and L1(UAG)↝L2(UAA) of the metazoan classes showed a nearly coinciding set of several nodes with increased log-likelihood values: Bilateria, Protostomia, Protostomia+Platyhelminthes, Porifera, Deuterostomia, Eleutherozoa, a molluscan group and several leaf nodes (Supplement 11.4 and 11.5). Gene orders support an L2(UAA)↝L1(UAG) remolding between the emergence of Bilateria and the Ecdysozoa since in Ecdysozoa *trnL1* took the place of *trnL2*. Also several of the independent remoldings in Ecdysozoa and Lophotrochozoa are supported by the gene orders of the two leucine tRNAs.

Taken together, the MLRD results and gene order data support multiple deep metazoan leucine remoldings, i.e. for the Protostomia, Ambulacraria and Porifera. This is consistent with previous reports (24,28,31). Multiple re-remoldings, in particular within Protostomia, e.g. within Mollusca and Eumalacostraca, are also well supported. Even earlier L1/L2 remolding events in the animal lineage are indicated by both, the results of MLRD and the conspicuously high similarity of all metazoan leucine tRNAs (see Supplement 11.4 and 9.7). A conclusive analysis of these issues would require a further refined analyses including fungal data, and exceeds the scope of the present study.

## CONCLUSION

We have presented a new method to identify tRNA remolding events in metazoan mitochondrial genomes. The comprehensive analysis presented here was based on the structural annotations of tRNAs that became available for all Metazoa generated by MITOS (58). Our method searches for statistically significant similarities between alloacceptor tRNAs and dissimilarities of isoacceptor tRNAs. It successfully identified several remoldings that are know from the literature and obtained a number of novel remolding events. Since the set of criteria we apply are quite strict, one would expect many false negatives. Thus further fine-tuning of the criteria and a more detailed analysis of the candidates might increase the recall.

Since the remolding candidate selection method relies on observing exceptional tRNA similarities in the given species, sampling events that are ancestral to a large fraction of the sample cannot be detected. This problem likely occurred in (31) where two pairs of *trnR* and *trnT* have been reported as exceptional similarities within Porifera although all *trnR* and *trnT* have exceptional similarities when considering all Metazoa. Likewise our method necessarily missed putative remolding events that are ancestral to Metazoa as indicated by multiple highly similar tRNA pairs.

Additionally, a novel maximum likelihood based method (MLRD) has been developed to test hypotheses on ancestral remolding events. With this method the phylogenetic vicinity of the detected remolding candidates has been explored. Thereby, many of the remolding candidates could be traced to ancestral remolding events. The resolution of the method for both, the exact position and direction of the remolding, is influenced by many factors such as

- the difficulty in selecting a proper outgroup,
- problems in the species tree,
- species sampling and
- the high similarity between the tRNAs that might be caused by more ancestral events.

Currently, the assumption of a single remolding event in the analyzed subtree is a limiting factor, which however can be alleviated to a certain extent by iteratively removing all remolded subtrees at the expense of a substantial reduction of the data that can be used to investigate the early branchings.

Remolding influences the frequencies of the codons corresponding to the acceptor and donor tRNA. Furthermore, adaption of the remolded tRNAs has been observed, in particular in the acceptor and anticodon arm where most of the known identity elements are located. Both effects, might be explained by misaminoacylation of the remolded tRNAs due to minor identity elements. The remolded tRNAs might prove useful for analyzing tRNA identity elements.

The detected remolding events stress that tRNA remolding is an important factor in the evolution of Metazoan mitogenomes that affects all lineages and needs to be considered. In summary, we demonstrated that sequence and structure information of tRNA genes can be used to detect novel remolding events. Together, the methods presented here can provide detailed predictions that may serve as a starting point for detailed investigations into the evolution of mitochondrial tRNAs.

## SUPPLEMENTARY DATA

Supplementary Data are available at NAR Online.

SUPPLEMENTARY DATA
