# Supplementary material for: Towards a comprehensive picture of alloacceptor tRNA remolding in metazoan mitochondrial genomes
Source: Nucleic Acids Res. 2015 Jul 30;43(16):8044–56. doi: 10.1093/nar/gkv746 (PMC4783518; doi:10.1093/nar/gkv746)
Supplement: SUPPLEMENTARY DATA [file supp_43_16_8044__index.html]

Towards a comprehensive picture of alloacceptor tRNA remolding in metazoan mitochondrial genomes — Towards a comprehensive picture of alloacceptor tRNA remolding in metazoan mitochondrial genomes — SUPPLEMENTARY DATA 

# Towards a comprehensive picture of alloacceptor tRNA remolding in metazoan mitochondrial genomes

## SUPPLEMENTARY DATA

- SUPPLEMENTARY DATA
