# Supplementary material for: Towards a comprehensive picture of alloacceptor tRNA remolding in metazoan mitochondrial genomes
Source: Nucleic Acids Res. 2015 Jul 30;43(16):8044–56. doi: 10.1093/nar/gkv746 (PMC4783518; doi:10.1093/nar/gkv746)
Supplement: SUPPLEMENTARY DATA [file supp_gkv746_nar-00403-r-2015-File002.pdf]

Supplementary Material: Towards a More Comprehensive Picture of Alloacceptor tRNA Remolding in Metazoan Mitochondrial Genomes

Contents

1 Methods **2**
1.1 `Infernal` Structural RNA Family Models . . . 2
1.2 Separation Between Distributions in a Mixture Setting . . . 2
1.3 Data Set Construction . . . 2
1.3.1 MITOS – Reannotation . . . 2
1.3.2 NCBI Taxonomy . . . 2
1.3.3 Multiple Sequence Alignments . . . 2
1.3.4 Automatic Outgroup Selection . . . 3
1.3.5 Leucine Data Set . . . 3

2 Remolding Candidates in $\mathcal{P}$ **3**

3 Remolding Candidates in $\mathcal{R}$ **4**

4 Affected Positions, Codon Boxes, and Nucleotides **7**

5 Distance between remolded tRNAs in $\mathcal{R}$ **8**

6 Duplicated or deleted acceptor or donor tRNAs **8**

7 Remolding and Codon Frequencies **9**

8 Post remolding effects **9**

9 Eumalacostraca **11**
9.1 Peracarida trnW(uca)⇝trnG(ucc) . . . 11
9.2 Peracarida trnC(gca)⇝trnY(gua) . . . 12
9.3 Peracarida trnC(gca)⇝trnF(gaa) . . . 13
9.4 Hymenoptera trnC(gca)⇝trnY(gua) . . . 14
9.5 Peracarida+Decapoda trnC(gca)⇝trnY(gua) . . . 15
9.6 Eumalacostraca trnQ(uug)⇝trnE(uuc) . . . 16
9.7 Mean Pairwise Similarities . . . 19

10 Porifera **20**
10.1 trnS2(uga)⇝trnY(gua) . . . 20
10.2 trnT(ugu)⇝trnR(ucu) . . . 21
10.3 trnT(ugu)⇝trnR(ucg) . . . 22
10.4 trnK(uuu)⇝trnN(ugu) . . . 23
10.5 trnK(uuu)⇝trnC(gca) . . . 24
10.6 trnT(ugu)⇝trnV(uac) . . . 25
10.7 trnV(uac)⇝trnT(ugu) . . . 26

11 Leucine remolding **27**
11.1 Eumalacostraca . . . 27
11.1.1 Eumalacostraca trnL2(uaa)⇝trnL1(uag) . . . 27
11.1.2 Eumalacostraca trnL1(uag)⇝trnL2(uaa) . . . 29
11.2 Ambulacraria trnL2(uaa)⇝trnL1(uag) . . . 30
11.3 Mollusca . . . 32
11.3.1 trnL2(uaa)⇝trnL1(uag) . . . 33
11.3.2 trnL1(uag)⇝trnL2(uaa) . . . 34
11.4 Metazoa . . . 35
11.4.1 trnL2(uaa)⇝trnL1(uag) . . . 35
11.4.2 trnL1(uag)⇝trnL2(uaa) . . . 37

11.5 Porifera trnL2(uaa)⇝trnL1(uag) . . . 38

12 Amphibia trnK(uuu)⇝trnT(ugu) **39**

13 Caption for all figures **41**

1 Methods

1.1 `Infernal` Structural RNA Family Models

`Infernal` [1] combines a multiple sequence alignment with secondary structure information and prior information on nucleotide and base pair probabilities. The inclusion of prior probabilities derived from a general structural RNA background prevents overspecialization of models with few known sequence members in a family, and therefore limited covariation information. Models with few sequences are more influenced by the prior than models with many member sequences. Sequence, structure, and prior information are combined into bitscore log-odds for each consensus nucleotide or base pair.

1.2 Separation Between Distributions in a Mixture Setting

When asking whether a bitscore provides significant evidence for remolding when compared to two bitscore distributions, we implicitly make the assumption that both distributions are well separated in their mixture.

Exact methods (e.g. the Kullback Leibler divergence to measure the number of bits to express one distribution in terms of the other or the Bhattacharyya distance, which measures the similarity of the two distributions) do exist. For efficiency, we rely here on simplifying assumptions and use the test statistic $D_{\mu_1,\mu_2,\sigma_1,\sigma_2} = \frac{|\mu_1-\mu_2|}{\sigma_1+\sigma_2}$. The parameters μ_k and σ_k, $k = 1, 2$, are the means and standard deviations of the bitscore distributions. For each *trnX*, we compared the distributions of the following scores: i) $S(Y_i|X_i)$ and ii) $S(X_i|X_j)$, with $j = C_{XY}(i)$, for all species i and *trnY*. For these distributions the smallest value of $D_{\mu_1,\mu_2,\sigma_1,\sigma_2}$ is 1.95. So, the bitscore distributions obtained for equal and unequal tRNA pairs are well separated. Therefore bitscores can be used directly to distinguish remolded and unremolded tRNAs. Given suitably well-behaved unimodal distributions, we can assume that a significance level of 0.95 does indeed point toward high likelihood of remolding.

1.3 Data Set Construction

1.3.1 MITOS – Reannotation

Structural annotations of the tRNAs have been obtained with an optimized version of MITOS using the 21 covariance models from `MiTFi` [2]. The optimized version uses `Infernal` version 1.1 [1] for ncRNA prediction and incorporates a new two-stage RNA prediction pipeline. The fast 1st stage uses `cmsearch` with default parameters and the 2nd stage uses the slow maximum sensitivity mode of `cmsearch`, but is run only for ncRNAs that could not be found in the 1st stage.

1.3.2 NCBI Taxonomy

All but one member of sets of RefSeq entries that map to the same taxid have been removed, e.g., multiple strains of one species. Therefore, 24 entries have been removed. Multifurcations contained in the NCBI taxonomy were replaced with the topology obtained with neighbor joining for *nad5* of one member of each subtree rooted below the corresponding multifurcation. The *nad5* gene is known to be a reasonable choice for phylogeny reconstruction [3]. The neighbour joining tree was computed with `quick tree` [4] version 1.1 from an alignment that has been created with `ClustalW` [5] version 2.0.12 (both tools using default parameters). Due to the removal of one leaf that lacked the *nad5* gene (NC_015998) the final tree contained 3817 taxa. Modifications of the phylogeny have been realized with the ETE2 python module [6].

1.3.3 Multiple Sequence Alignments

Multiple sequence alignments containing donor and acceptor sequences of the ingroup and outgroup tRNA sequences are created based on the model of the donor tRNA using `cmalign` [1]. Structural alignments and subfamily logos [7] were created with TEXshade [8]. If the alignment is beyond the capabilities of TEXshade, i.e., around 100 sequences, only a random sample of it is presented.

1.3.4 Automatic Outgroup Selection

Outgroup species for a subtree T have been selected automatically by choosing a species o from the subtrees rooted at the sibling of the root of the subtree T such that for the donor X_o (all tRNAs in the remolded subtree came from this donor) holds: the average pairwise alignment score of X_o with all ingroup species X_i, with $i \in T$, is significantly lower (Wilcoxon signed-rank sum test with $p \leq 0.05$) than the distribution of pairwise alignment scores between ingroup species, i.e., X_i vs. X_j, with $i, j \in T$. Two outgroup species are determined by applying this method to the lowest common ancestor of the species in the data set, its parent, and if necessary (i.e., if no species fulfills the outgroup criterion) iteratively further nodes toward the root of the Metazoa. Pairwise alignment scores are obtained using `ClustalW` [5] version 2.1 with default parameters.

1.3.5 Leucine Data Set

The Leucine data set was constructed as follows. In order to get a balanced sample one species was selected at random for each class and additional Porifera have been included. Species for which no class was specified in the NCBI taxonomy were ignored. A placozoan mitogenome was used as outgroup. Since `MITOS` missed at least one of the leucine genes for placozoan mitogenomes the annotation of the *trnL1* in the chosen outgroup was taken from RefSeq.

2 Remolding Candidates in $\mathcal{P}$

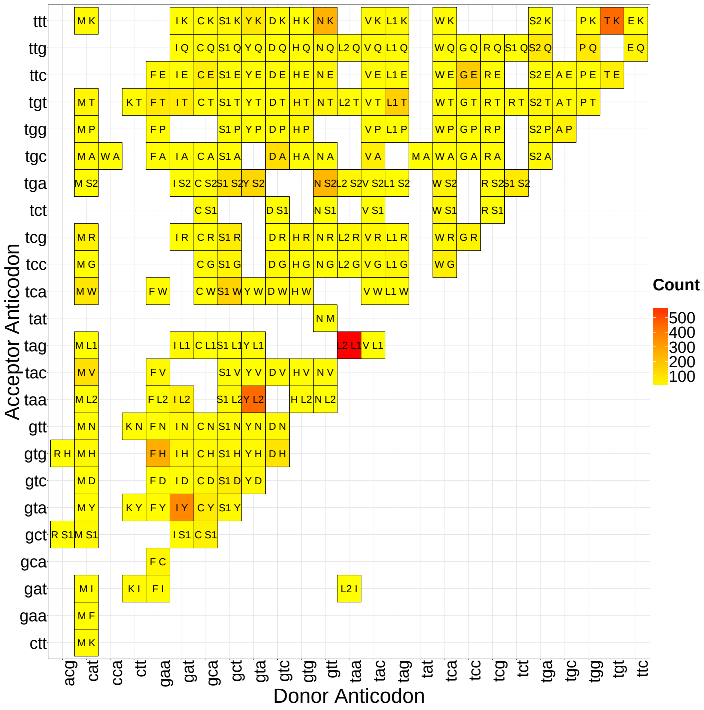

Figure 1: Remolding candidates in $\mathcal{P}$ (both directions are joined). The anticodon of a *trnQ(TTG)* and a *trnL2(TAA)* has been determined manually since `MITOS` was not able to detect the anticodon.

3 Remolding Candidates in $\mathcal{R}$

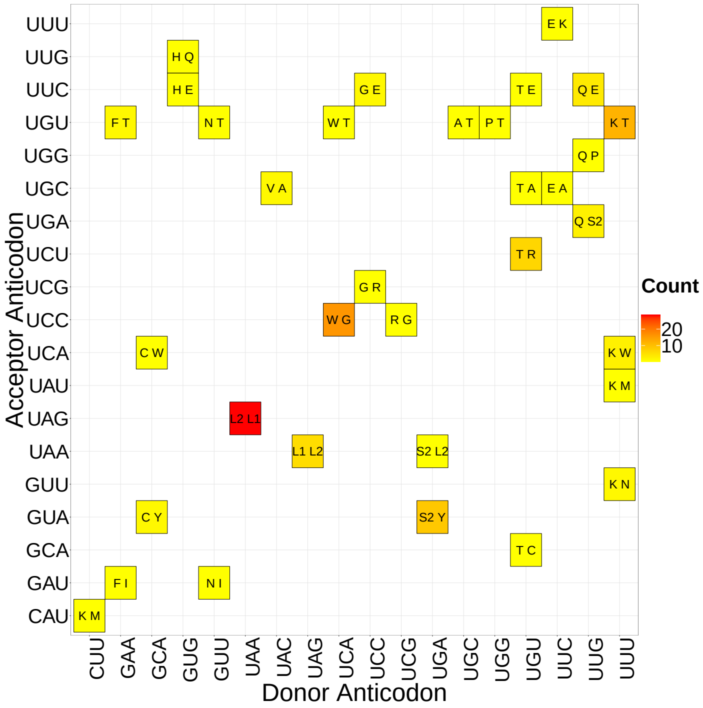

(a)

Figure 2: Remolding candidates in $\mathcal{R}$. The anticodon of a single *trnQ(TTG)* has been determined manually since `MITOS` was not able to detect the anticodon.

Accession	Name	Donor	Start	Stop	Acceptor	Start	Stop
NC_013881	*Echinocardium cordatum*	*E(uuc)*	4608	4677	*A(ugc)*	5185	5254
NC_015649	*Rhabdopleura compacta*	*S2(uga)*	3321	3392	*L2(uaa)*	7401	7472
NC_007627	*Gekko gecko*	*R(ucg)*	9840	9906	*G(ucc)*	9430	9499
NC_018135	*Dissostichus eleginoides*	*E(uuc)*	14665	14734	*K(uuu)*	13764	13830
NC_008448	*Galaxiella nigrostriata*	*H(gug)*	2303	2372	*Q(caa)*	13764	13830
NC_013752	*Microcosmus sulcatus*	*C(gca)*	1862	1927	*W(uca)*	10218	10282
NC_014588	*Thylacodes squamigerus*	*W(uca)*	6601	6669	*T(ugu)*	12559	12623
NC_013247	*Cymatium parthenopeum*	*N(guu)*	13627	13695	*T(ugu)*	8967	9033
NC_012435	*Pyramidella dolabrata*	*P(ugg)*	2767	2829	*T(ugu)*	11982	12045
NC_016185	*Salinator rhamphidia*	*Q(uug)*	8366	8427	*P(ugg)*	2755	2819
NC_011572	*Symphylella sp.*	*A(ugc)*	4361	4423	*T(ugu)*	11797	11859
NC_008323	*Vanhornia eucnemidarum*	*T(ugu)*	11827	11895	*C(gca)*	1211	1275
NC_011923	*Bombus hypocrita*	*H(gug)*	8707	8764	*E(uuc)*	6808	6863
NC_004529	*Melipona bicolor*	*G(ucc)*	5599	5665	*R(ucg)*	6021	6085
NC_012617	*Geisha distinctissima*	*T(ugu)*	9471	9532	*E(uuc)*	6016	6078
NC_014850	*Oscarella microlobata*	*F(gaa)*	14126	14199	*I(gau)*	3631	3704
NC_006894	*Axinella corrugata*	*T(ugu)*	9699	9772	*A(ugc)*	9371	9444
NC_010213	*Agelas schmidti*	*N(guu)*	8005	8076	*I(gau)*	18794	18865
NC_001453	*Strongylocentrotus purpuratus*	*L2(uaa)*	2086	2159	*L1(uag)*	1425	1497
NC_009940	*Strongylocentrotus droebachiensis*	*L2(uaa)*	2137	2210	*L1(uag)*	1463	1535
NC_009941	*Strongylocentrotus pallidus*	*L2(uaa)*	2130	2203	*L1(uag)*	1462	1534
NC_001572	*Paracentrotus lividus*	*L2(uaa)*	2111	2184	*L1(uag)*	1451	1523
NC_014452	*Stichopus sp.*	*L2(uaa)*	12450	12522	*L1(uag)*	11720	11792
NC_014454	*Stichopus horrens*	*L2(uaa)*	12450	12521	*L1(uag)*	11718	11790
NC_012616	*Apostichopus japonicus*	*L2(uaa)*	12375	12442	*L1(uag)*	11470	11542
NC_013432	*Parastichopus nigripunctatus*	*L2(uaa)*	12386	12453	*L1(uag)*	11472	11544
NC_005929	*Cucumaria miniata*	*L2(uaa)*	14046	14117	*L1(uag)*	3407	3478
NC_001878	*Florometra serratissima*	*L2(uaa)*	3371	3443	*L1(uag)*	1386	1457
NC_007690	*Phanogenia gracilis*	*L2(uaa)*	3166	3237	*L1(uag)*	1385	1456
NC_020775	*Lithidiopsis carinatus*	*L2(uaa)*	2956	3021	*L1(uag)*	12607	12673

NC_021610	*Shirakiacris shirakii*	*L2(uaa)*	2964	3030	*L1(uag)*	12606	12672
NC_013826	*Traulia szetschuanensis*	*L2(uaa)*	2961	3027	*L1(uag)*	12581	12647
NC_006678	*Gryllotalpa orientalis*	*L2(uaa)*	2960	3025	*L1(uag)*	12501	12566
NC_011302	*Gryllotalpa pluvialis*	*L2(uaa)*	2960	3025	*L1(uag)*	12505	12570
NC_022922	*Corythucha ciliata*	*L2(uaa)*	2889	2953	*L1(uag)*	12282	12347
NC_012429	*Orius niger*	*L2(uaa)*	2888	2954	*L1(uag)*	12317	12383
NC_013976	*Eophreatoicus sp.*	*L2(uaa)*	62	123	*L1(uag)*	125	190
NC_019609	*Neaxius glyptocercus*	*L2(uaa)*	1603	1668	*L1(uag)*	1538	1601
NC_020023	*Upogebia pusilla*	*L2(uaa)*	1534	1602	*L1(uag)*	1603	1668
NC_019607	*Upogebia major*	*L2(uaa)*	1538	1606	*L1(uag)*	1607	1672
NC_019606	*Austinogebia edulis*	*L2(uaa)*	1542	1608	*L1(uag)*	1611	1678
NC_020351	*Nihonotrypaea japonica*	*L2(uaa)*	1599	1663	*L1(uag)*	1534	1599
NC_019610	*Nihonotrypaea thermophila*	*L2(uaa)*	1602	1666	*L1(uag)*	1538	1602
NC_020029	*Paralithodes camtschaticus*	*L2(uaa)*	1619	1687	*L1(uag)*	1551	1618
NC_021458	*Paralithodes brevipes*	*L2(uaa)*	5123	5191	*L1(uag)*	5055	5122
NC_017757	*Bothriocroton undatum*	*L2(uaa)*	6767	6828	*L1(uag)*	14342	14402
NC_008426	*Erignathus barbatus*	*S2(uga)*	6888	6957	*Y(gua)*	5277	5345
NC_014176	*Trioceros melleri*	*S2(uga)*	6823	6896	*Y(gua)*	5213	5284
NC_014853	*Pseudocorticium jarrei*	*S2(uga)*	17825	17910	*Y(gua)*	3746	3829
NC_014850	*Oscarella microlobata*	*S2(uga)*	17721	17806	*Y(gua)*	3706	3789
NC_009090	*Oscarella carmela*	*S2(uga)*	17681	17766	*Y(gua)*	3723	3806
NC_014886	*Oscarella malakhovi*	*S2(uga)*	17686	17771	*Y(gua)*	3723	3806
NC_014856	*Oscarella viridis*	*S2(uga)*	17793	17878	*Y(gua)*	3739	3822
NC_014888	*Oscarella tuberculata*	*S2(uga)*	17615	17700	*Y(gua)*	3680	3763
NC_014863	*Oscarella lobularis*	*S2(uga)*	17614	17699	*Y(gua)*	3681	3764
NC_006288	*Bipes canaliculatus*	*K(uuu)*	7646	7713	*T(ugu)*	15293	15362
NC_020142	*Oscaecilia ochrocephala*	*K(uuu)*	7698	7771	*T(ugu)*	15248	15317
NC_006335	*Plethodon elongatus*	*K(uuu)*	7612	7684	*T(ugu)*	17723	17792
NC_006344	*Phaeognathus hubrichti*	*K(uuu)*	7667	7739	*T(ugu)*	15241	15310
NC_008077	*Batrachuperus londongensis*	*K(uuu)*	7724	7795	*T(ugu)*	15322	15390
NC_008083	*Batrachuperus pinchonii*	*K(uuu)*	7730	7801	*T(ugu)*	15332	15400
NC_009335	*Hynobius arisanensis*	*K(uuu)*	7735	7804	*T(ugu)*	15332	15401
NC_008088	*Hynobius chinensis*	*K(uuu)*	7746	7817	*T(ugu)*	15350	15420
NC_013762	*Hynobius guabangshanensis*	*K(uuu)*	7746	7817	*T(ugu)*	15350	15420
NC_010224	*Hynobius quelpaertensis*	*K(uuu)*	7736	7807	*T(ugu)*	15337	15407
NC_009258	*Bombina variegata*	*K(uuu)*	7721	7793	*T(ugu)*	15313	15382
NC_006689	*Bombina orientalis*	*K(uuu)*	7721	7793	*T(ugu)*	15313	15382
NC_008777	*Furcifer oustaleti*	*F(gaa)*	0	69	*T(ugu)*	15107	15170
NC_011394	*Ophiophagus hannah*	*F(gaa)*	0	62	*T(ugu)*	16036	16103
NC_020570	*Balearica pavonina*	*K(uuu)*	7754	7824	*M(uau)*	14790	14859
NC_003057	*Limulus polyphemus*	*K(cuu)*	2224	2294	*M(cau)*	13706	13776
NC_007700	*Malacochersus tornieri*	*Q(uug)*	5848	5919	*S2(uga)*	8948	9019
NC_011755	*Nezara viridula*	*Q(uug)*	66	134	*S2(uga)*	11446	11515
NC_012464	*Yemmalysus parallelus*	*Q(uug)*	61	130	*S2(uga)*	11369	11439
NC_011121	*Echinococcus canadensis*	*V(uac)*	7278	7341	*A(ugc)*	7346	7412
NC_011122	*Echinococcus ortleppi*	*V(uac)*	7271	7334	*A(ugc)*	7339	7405
NC_022846	*Laternula elliptica*	*C(gca)*	5523	5594	*Y(gua)*	11431	11493
NC_013819	*Onisimus nanseni*	*C(gca)*	12074	12134	*Y(gua)*	13488	13546
NC_015993	*Mytilus californianus*	*Q(uug)*	15346	15413	*E(uuc)*	15118	15183
NC_007010	*Marsupenaeus japonicus*	*Q(uug)*	77	147	*E(uuc)*	6208	6277
NC_015073	*Macrobrachium nipponense*	*Q(uug)*	14472	14540	*E(uuc)*	4725	4794
NC_021971	*Nautilocaris saintlaurentae*	*Q(uug)*	14578	14646	*E(uuc)*	4741	4810
NC_022194	*Fulvia mutica*	*L1(uag)*	11214	11278	*L2(uaa)*	11150	11216
NC_006895	*Nesomachilis australica*	*L1(uag)*	12609	12670	*L2(uaa)*	2997	3064
NC_023093	*Leptomyrmex pallens*	*L1(uag)*	11244	11312	*L2(uaa)*	1528	1595
NC_020025	*Corallianassa coutierei*	*L1(uag)*	1534	1600	*L2(uaa)*	1600	1665
NC_007379	*Geothelphusa dehaani*	*L1(uag)*	1213	1278	*L2(uaa)*	17467	17531
NC_014671	*Ammothea carolinensis*	*L1(uag)*	12366	12430	*L2(uaa)*	12299	12363
NC_016866	*Corcyra cephalonica*	*G(ucc)*	5507	5572	*E(uuc)*	6203	6270
NC_015480	*Calinaga davidis*	*G(ucc)*	5507	5573	*E(uuc)*	6233	6301
NC_014687	*Caprella scaura*	*W(uca)*	14954	15014	*G(ucc)*	15016	15079
NC_014492	*Caprella mutica*	*W(uca)*	1229	1289	*G(ucc)*	1291	1353
NC_019662	*Pseudoniphargus daviui*	*W(uca)*	1220	1283	*G(ucc)*	1285	1349
NC_017760	*Gammarus duebeni*	*W(uca)*	15529	15592	*G(ucc)*	15590	15651

NC_023104	*Eulimnogammarus verrucosus*	*W(uca)*	1510	1572	*G(ucc)*	1570	1630
NC_013819	*Onisimus nanseni*	*W(uca)*	14599	14665	*G(ucc)*	14667	14732
NC_016192	*Gondogeneia antarctica*	*W(uca)*	7078	7144	*G(ucc)*	8976	9040
NC_019660	*Metacrangonyx remyi*	*W(uca)*	3176	3239	*G(ucc)*	3239	3301
NC_013032	*Metacrangonyx longipes*	*W(uca)*	2546	2610	*G(ucc)*	2612	2674
NC_019653	*Metacrangonyx repens*	*W(uca)*	2554	2618	*G(ucc)*	2618	2680
NC_019659	*Metacrangonyx panousei*	*W(uca)*	2799	2863	*G(ucc)*	2864	2928
NC_019658	*Metacrangonyx longicaudus*	*W(uca)*	3030	3094	*G(ucc)*	3096	3159
NC_019657	*Metacrangonyx spinicaudatus*	*W(uca)*	2548	2612	*G(ucc)*	2613	2676
NC_019654	*Metacrangonyx dominicanus*	*W(uca)*	2990	3054	*G(ucc)*	3055	3116
NC_019656	*Metacrangonyx ilvanus*	*W(uca)*	3033	3097	*G(ucc)*	3099	3162
NC_019655	*Metacrangonyx goulmimensis*	*W(uca)*	2755	2820	*G(ucc)*	2821	2884
NC_018602	*Angiostrongylus vasorum*	*K(uuu)*	8965	9026	*W(uca)*	6510	6568
NC_013827	*Teladorsagia circumcincta*	*K(uuu)*	9489	9551	*W(uca)*	6899	6956
NC_007934	*Anisakis simplex*	*K(uuu)*	1492	1554	*W(uca)*	12399	12457
NC_008833	*Placozoan sp.*	*K(uuu)*	4623	4696	*N(guu)*	3543	3616
NC_006894	*Axinella corrugata*	*K(uuu)*	5034	5106	*N(guu)*	4829	4901
NC_014853	*Pseudocorticium jarrei*	*T(ugu)*	12741	12814	*R(ucu)*	15188	15261
NC_014856	*Oscarella viridis*	*T(ugu)*	16402	16475	*R(ucu)*	15203	15276
NC_010209	*Ptilocaulis walpersi*	*T(ugu)*	11871	11946	*R(ucu)*	7016	7091
NC_010201	*Amphimedon compressa*	*T(ugu)*	13827	13901	*R(ucu)*	11391	11465
NC_010206	*Callyspongia plicifera*	*T(ugu)*	14167	14240	*R(ucu)*	11813	11885
NC_010211	*Xestospongia muta*	*T(ugu)*	14116	14189	*R(ucu)*	11708	11781
NC_010210	*Ectyoplasia ferox*	*T(ugu)*	6925	6999	*R(ucu)*	11726	11800

Table 1: Remolding candidates predicted in $\mathcal{R}$.

4 Affected Positions, Codon Boxes, and Nucleotides

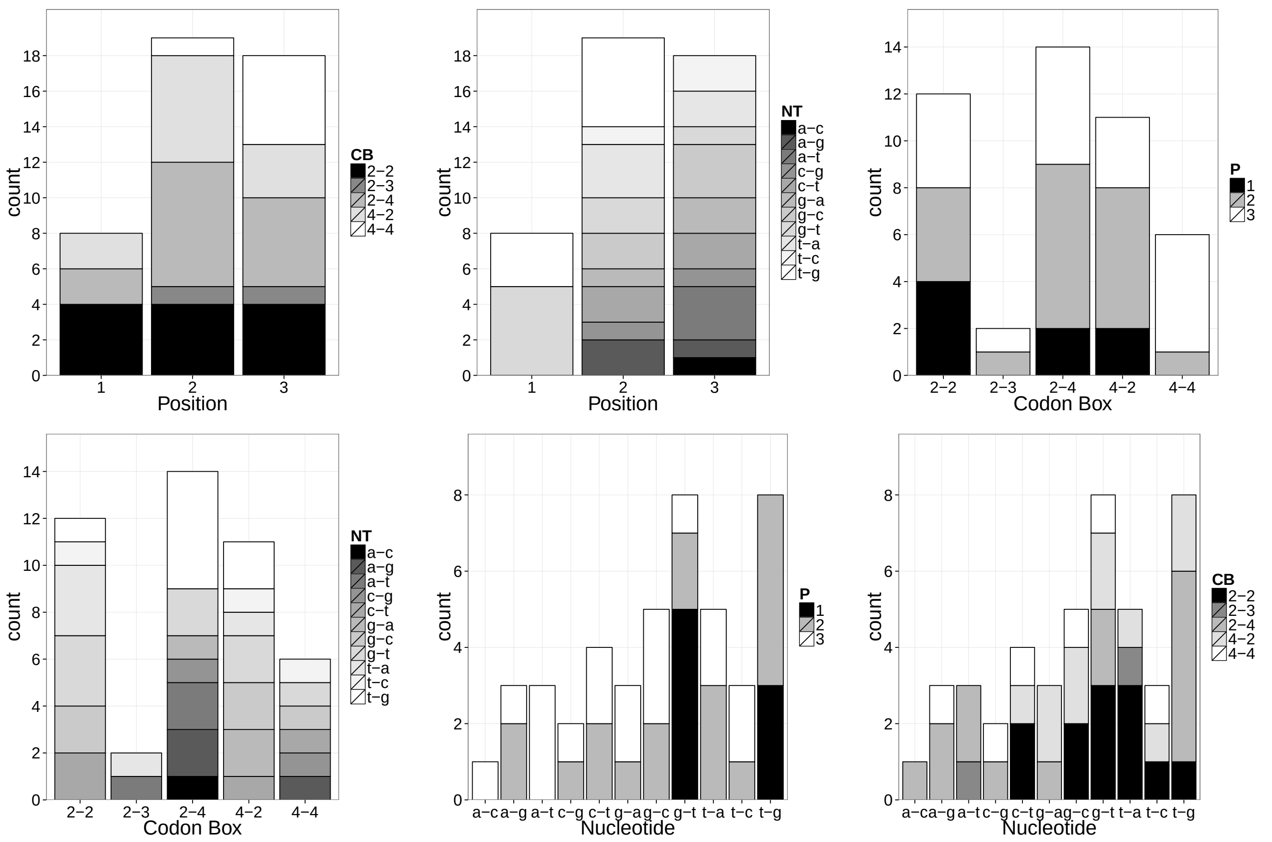

Figure 3: Number of unique remolding events wrt. change of the codon box (CB), i.e., (2/3/4 fold degenerated), Anticodon Position (P), and changed nucleotides (NT); remoldings that affect multiple positions are counted multiple times. If a remolding changes multiple positions it is depicted multiple times.

5 Distance between remolded tRNAs in $\mathcal{R}$

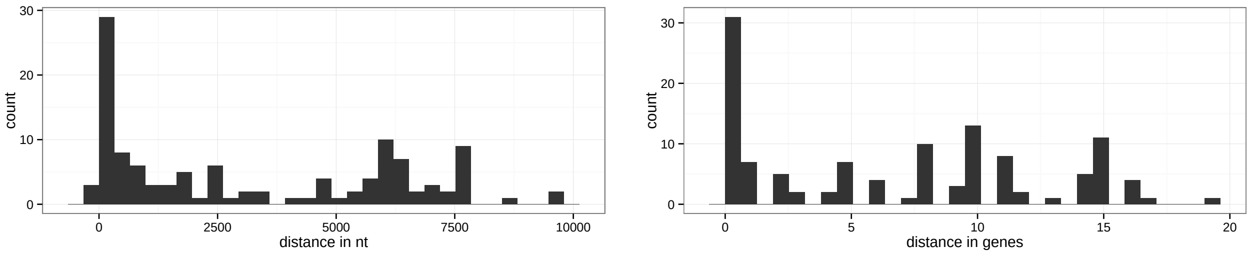

Figure 4: Distribution of the distance of tRNA pairs from $\mathcal{R}$ in nt (left) and number of genes (right).

6 Duplicated or deleted acceptor or donor tRNAs

We scanned all necessary mitogenomes for duplicated or deleted donor and acceptor tRNAs, to address the question of initiated but unfinished remolding events. We constructed subtrees of the topology: (..(..R..,..L..)..,..O..), where R is one of the 118 detected remolding cases, L marks the sister subtree of R, and O is a subtree of basal unremolded cases. Based on the annotations provided by MITOS, we counted in L the number of species with an duplicated or deleted donor or acceptor tRNA, respectively. All species in O were checked to stay in an ancestral unremolded state.

Remolding Type	2xD	0xD	2xA	0xA	2xD 0xA	Comment
NC_014853:trnT⇝trnR	6/6	0/6	6/6	0/6	0/6	
NC_014856:trnT⇝trnR	2/2	0/2	2/2	0/2	0/2	
NC_010201:trnT⇝trnR	0/1	1/1	1/1	0/1	0/1	Multiple lost tRNAs (PMID 17053047)
NC_010210:trnT⇝trnR	0/2	0/2	2/2	0/2	0/2	
NC_010206:trnT⇝trnR	0/1	0/1	1/1	0/1	0/1	
NC_010211:trnT⇝trnR	0/1	0/1	1/1	0/1	0/1	
NC_010209:trnT⇝trnR	0/1	0/1	1/1	0/1	0/1	
NC_015993:trnQ⇝trnE	1/3	0/3	0/3	0/3	0/3	
NC_008323:trnT⇝trnC	0/16	2/16	0/16	2/16	0/16	All losses are false negatives of MITOS
NC_010213:trnN⇝trnI	0/12	1/12	0/12	2/12	0/12	N and 1xI:FN, the other I lost (see PMID 17053047)
NC_006335:trnK⇝trnT	0/2	0/2	1/2	0/2	0/2	there is also a duplicate in a sister but not necessarily the same
NC_011572:trnA⇝trnT	0/1	0/1	0/1	1/1	0/1	
NC_011923:trnH⇝trnE	0/1	0/1	0/1	1/1	0/1	FN
NC_006894:trnT⇝trnA	0/1	0/1	0/1	1/1	0/1	FN

Table 2: Closely related species where the acceptor (A) or donor (D) is duplicated (2x) or deleted (0x).

We detected only a few cases (2) where duplications of the donor and/or the acceptor happened (e.g. NC_014853:trnT⇝trnR). Most of the deletions of donor or acceptor tRNAs could be confirmed as false negatives of the MITOS annotation. However, no significant results were found that would confirm/deny any case of an initiated but unfinished remolding event (2xD, 0xA).

7 Remolding and Codon Frequencies

Let $f_{X,C}$ be the frequency of the codon corresponding to the anticodon of $trnX$ in the mitogenome of species C and $F_{X,C}$ be the frequency of the codons of the codon box that includes the codon corresponding to the anticodon of $trnX$ in the mitogenome of species C. The normalized difference of the codon frequencies f (respectively F) have been calculated for each tRNA with respect to an unremolded closely related species, i.e., a species where $trnX$ is not in $\mathcal{U}$, where $\mathcal{U} = \{trnX : trnX \notin N \cup P \cup R\}$. That is, $\delta_{X,C} = \frac{f_{X,C} - f_{X,D}}{\max(f_{X,C}, f_{X,D})}$ (respectively $\Delta_{X,C} = \frac{F_{X,C} - F_{X,D}}{\max(F_{X,C}, F_{X,D})}$) have been calculated for all $trnX$ of species C and a closely related species D. The distribution of these values is shown in Figure 5 for remolded and unremolded tRNAs.

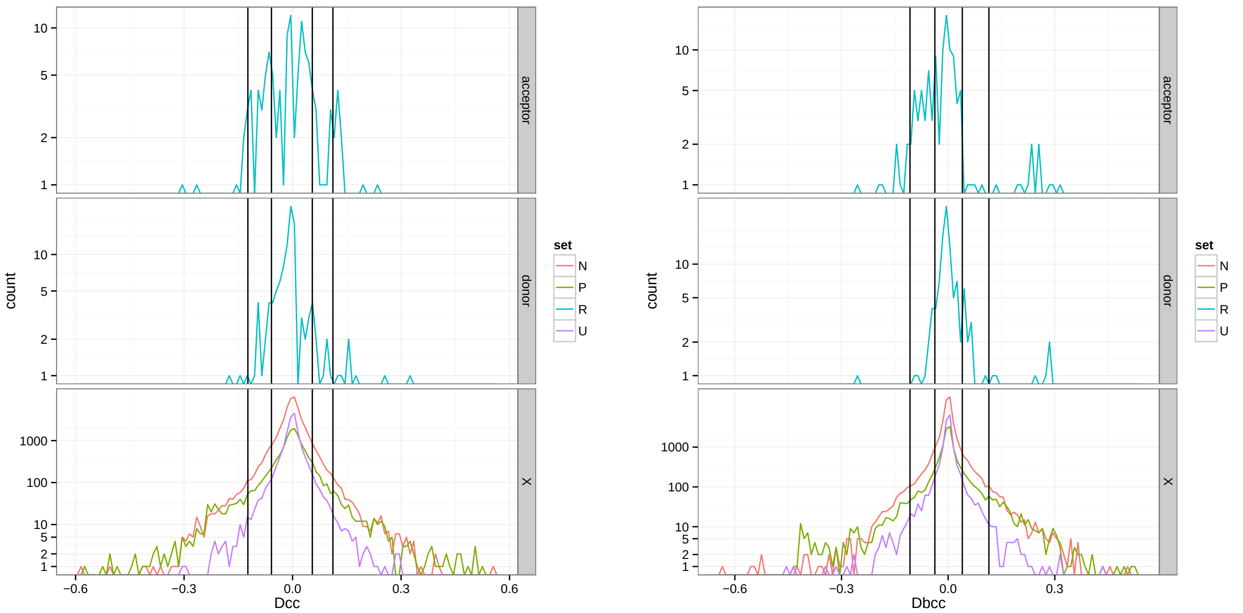

Figure 5: Distribution of the normalized differences of the codon frequencies f (left) and F (right) for the acceptor and donor of the remolding candidates in $\mathcal{R}$ and other tRNAs in the sets $\mathcal{N}$, $\mathcal{P}$, and $\mathcal{U}$

8 Post remolding effects

data set	remolding	comparison
Amphibia	K⇝T	P
Echinoderms+Hemichordates	L2⇝L1	V
Eumalacostraca	L1⇝L2	F
Eumalacostraca	Q⇝E	P
Mollusca	L1⇝L2	F
Peracarida	C⇝Y	F
Peracarida	W⇝G	K
Porifera	L2⇝L2	P
Porifera	S2⇝Y	K
Porifera	T⇝R	H

Table 3: Data sets used for analyzing post remolding effects. Given are the data set, the analyzed remolding, and the used unremolded tRNA.

The combined alignments have been constructed using `cmalign` in glocal model without the use of the truncated alignment algorithm employing a combined tRNA covariance model. This model was built from an alignment combining the seed alignments of the different mitochondrial tRNA families. The seed alignments were merged manually, followed by realignments of all loop regions.

The information content of column i of an alignment is computed as $R_i = \log_2(4) + \sum f_{a,i} \log_2(f_{a,i})$, where $f_{a,i}$ is the frequency of character $a \in \{A, U, C, G\}$ in column i. The bits of character a are then given by $f_{a,i} R_i$.

9 Eumalacostraca

9.1 Peracarida trnW(uca)⇝trnG(ucc)

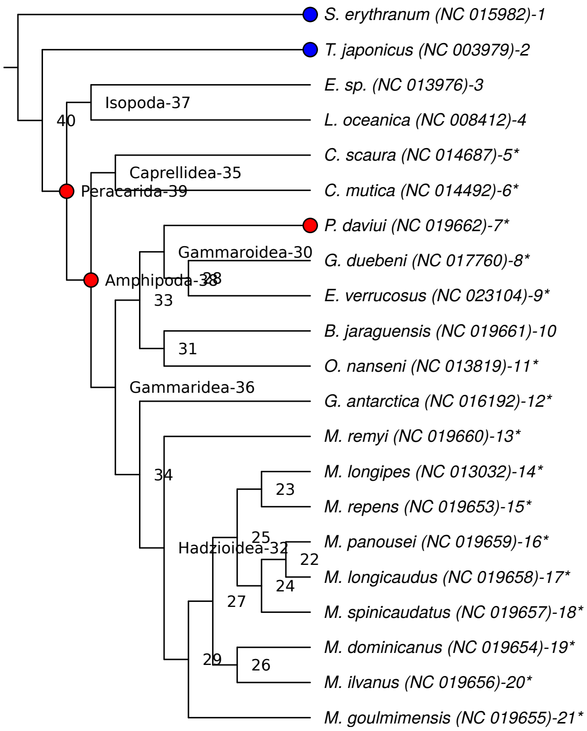

(a)

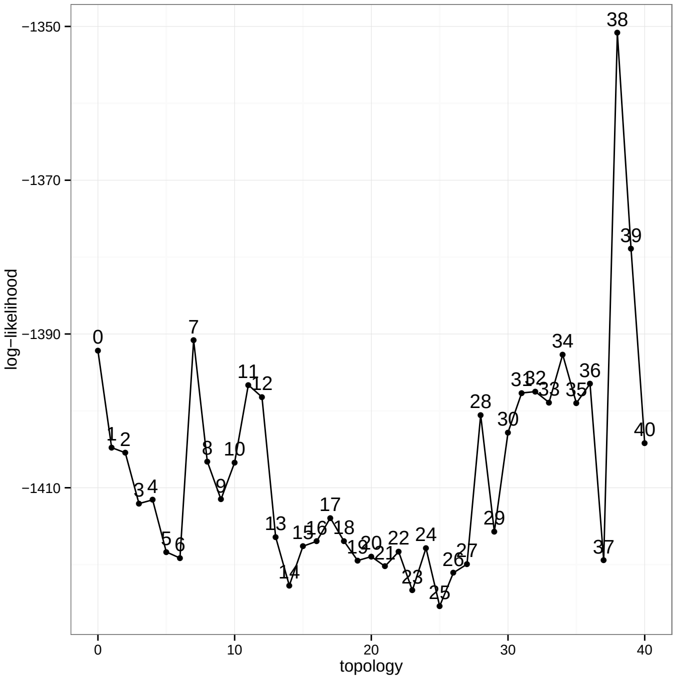

(b)

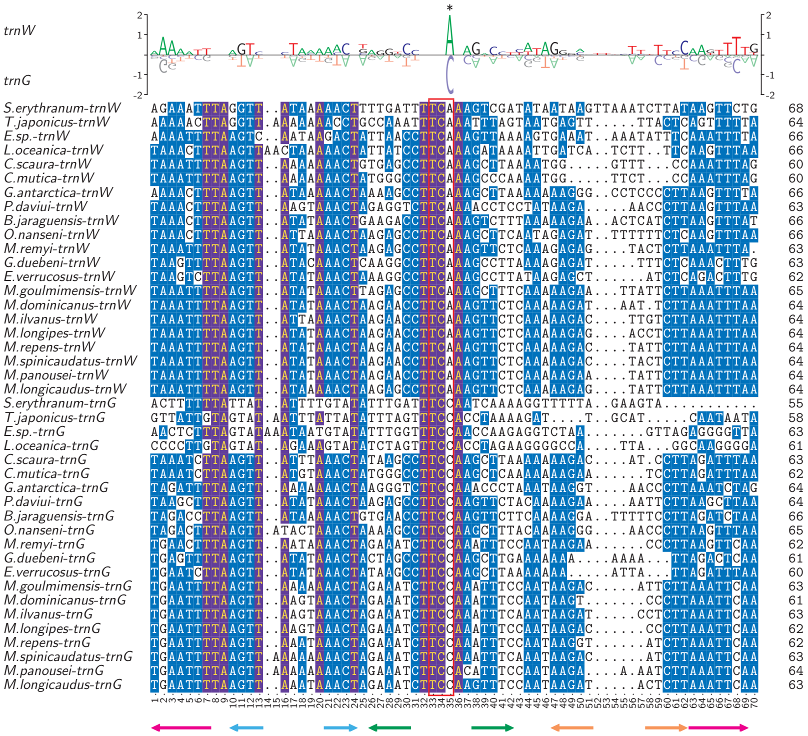

(c)

Figure 6: See Section 13

9.2 Peracarida trnC(gca)⇝trnY(gua)

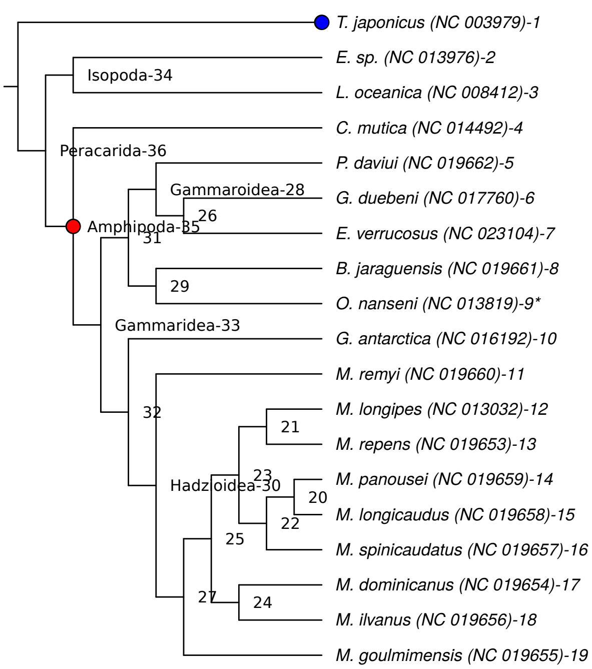

(a)

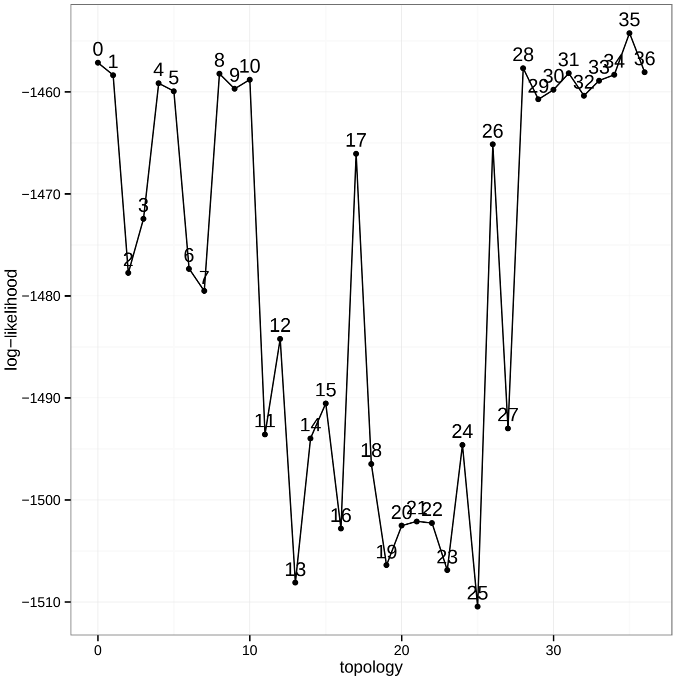

(b)

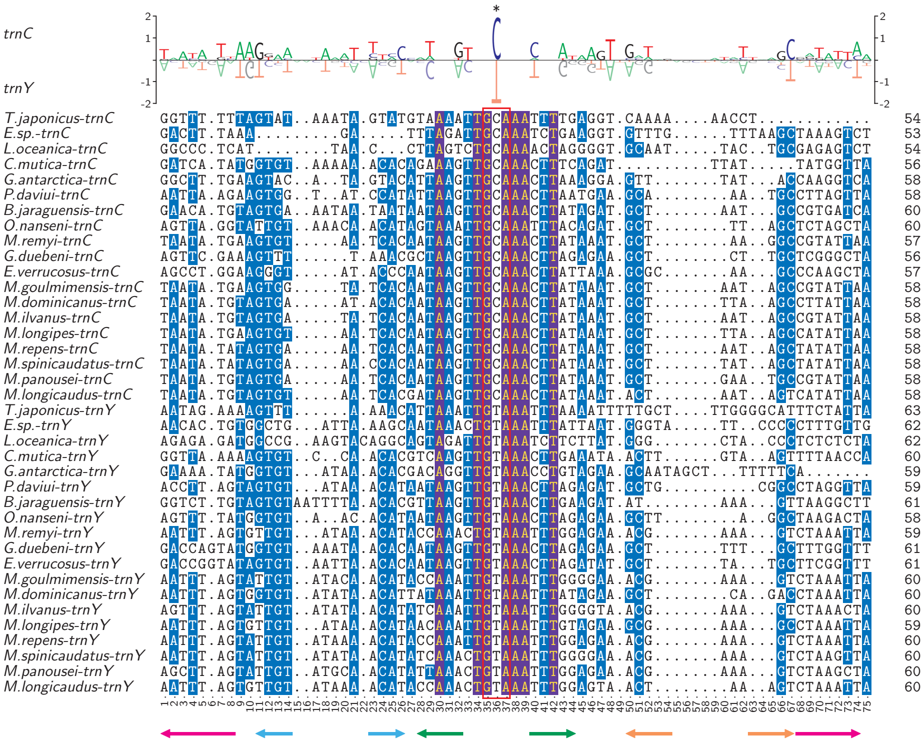

(c)

Figure 7: See Section 13

9.3 Peracarida trnC(gca)⇝trnF(gaa)

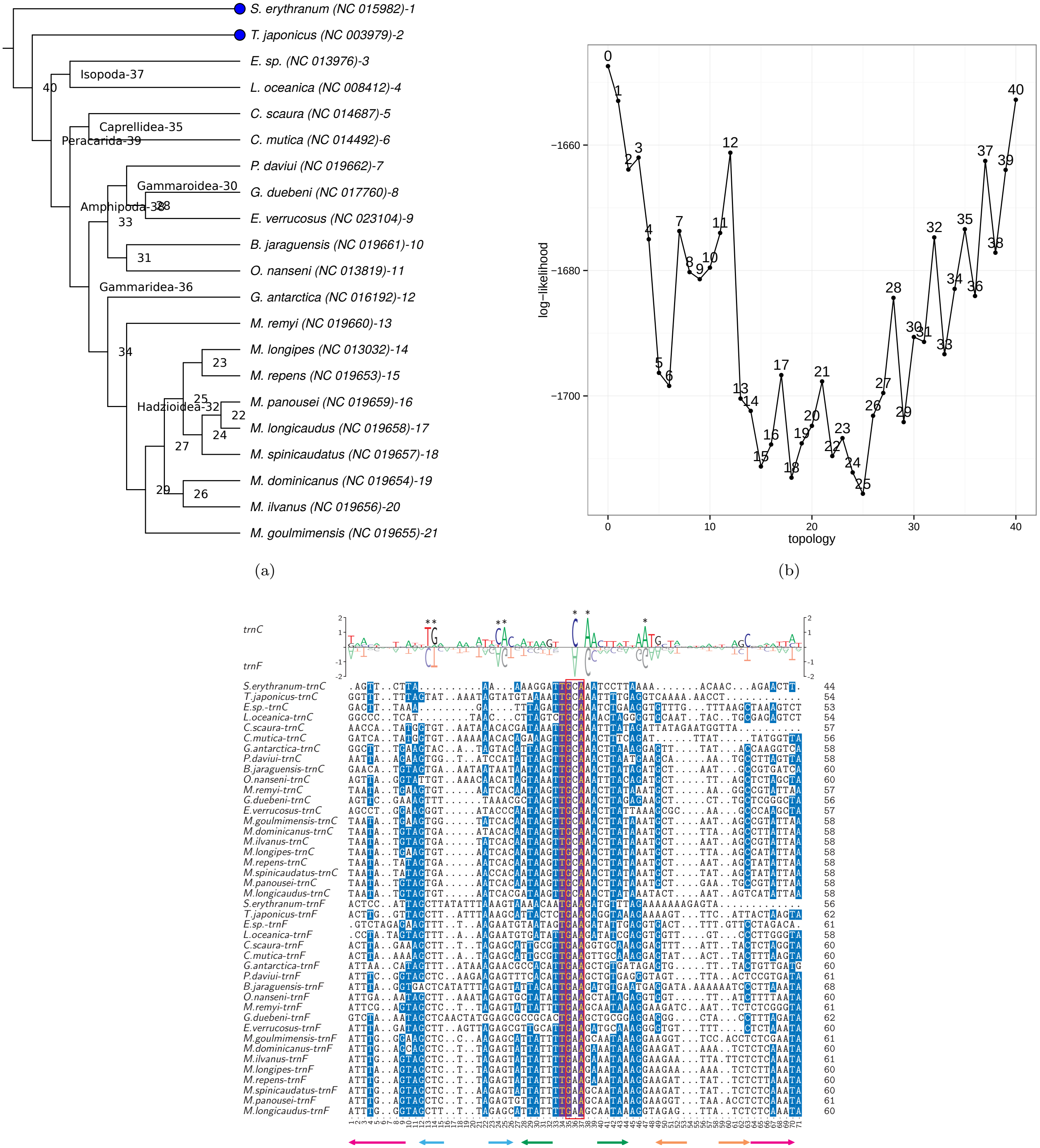

(a) (b) (c)

Figure 8: See Section 13

9.4 Hymenoptera trnC(gca)⇝trnY(gua)

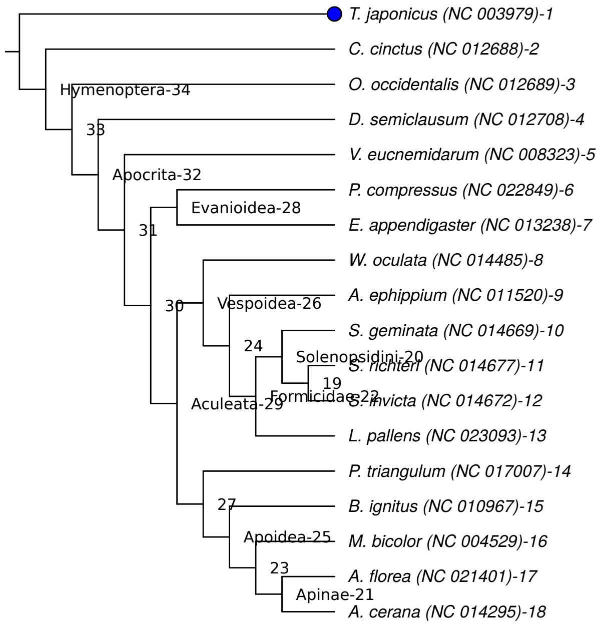

(a)

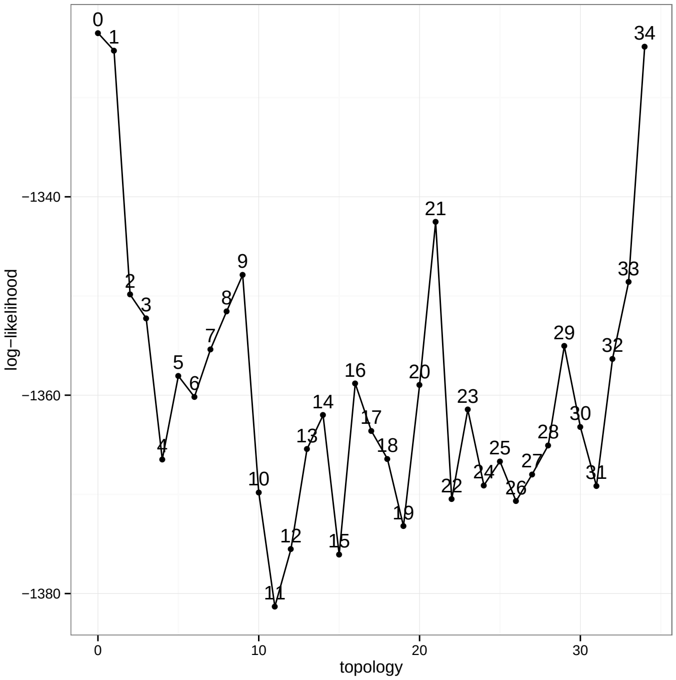

(b)

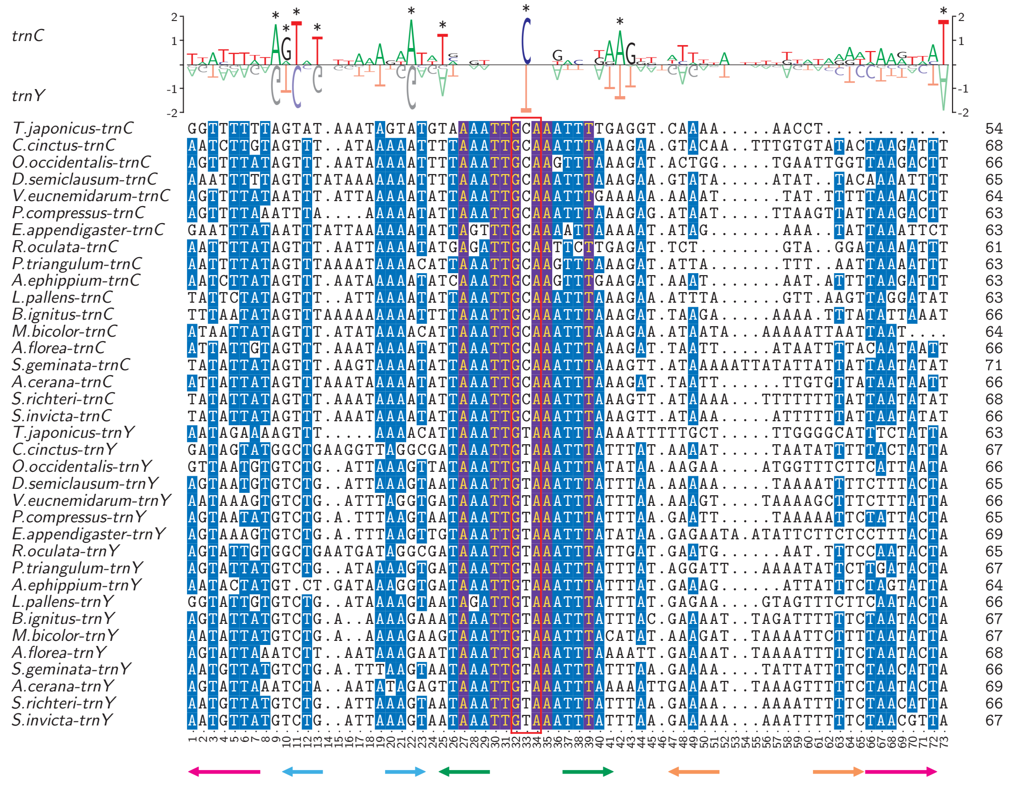

(c)

Figure 9: See Section 13

9.5 Peracarida+Decapoda trnC(gca)⇝trnY(gua)

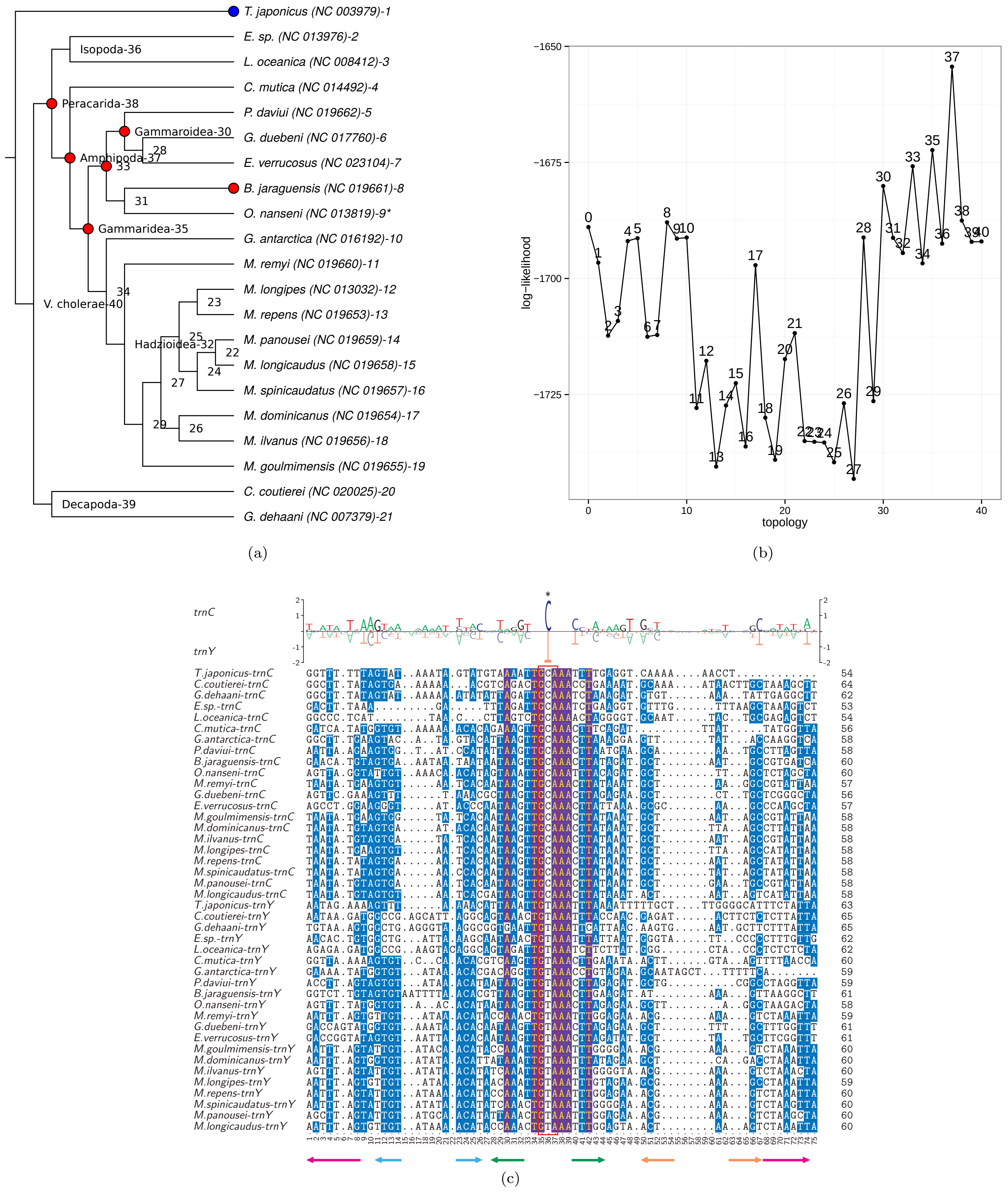

Figure 10: See Section 13

9.6 Eumalacostraca trnQ(uug)⇝trnE(uuc)

A Q(UUG)⇝E(UUC) remolding was detected in the mitogenomes of *Macrobrachium nipponense*, *Nautilocaris saintlaurentae* (Caridea) and *Marsupenaeus japonicus* (Penaeoidea). Separate `MLRD` analysis of the Eumalacostraca and Caridea showed no increased log-likelihood values, see Figure 11 and 12. The alignment of the Caridea showed 39 conserved columns, but also 20 columns that are tRNA specific. Only few conserved columns are present in the alignment of the Eumalacostraca. The two tRNAs are contained in two distinct tRNA clusters that are well preserved in Arthropoda, i.e., *trnA*, *trnR*, *trnN*, *trnS1*, *trnE*, *trnF* and *trnI*, *trnQ*, *trnM*, respectively. For all three species, the p-values for the 1st criterion are between 0.04 and 0.042. For the 2nd criterion they are between 0.054 and 0.058 (i.e., the p-value that the similarity is smaller than for the equal tRNAs from closely related species). These p-values are similar to those observed for the very convincing W⇝G remoldings in Amphipoda (0.04 and 0.06, respectively). Taken together, however, the observed tRNA specific sequence characteristics, the conserved position within the tRNA clusters, and the absence of increased log-likelihood values do not support tRNA remolding as cause for the observed exceptional similarity of *trnQ* and *trnE* of some Eumalacostraca. There are at least two possibilities to explain the tRNA specific nucleotide in the alignment: i) we observe indeed unremolded tRNAs or ii) mutations accumulated on the duplicated tRNAs before the speciation events took place. With larger numbers of mutations accumulating between the duplication and the speciation the two cases are more difficult to distinguish. This might be the source of the problems in the case of the Q⇝E remolding.

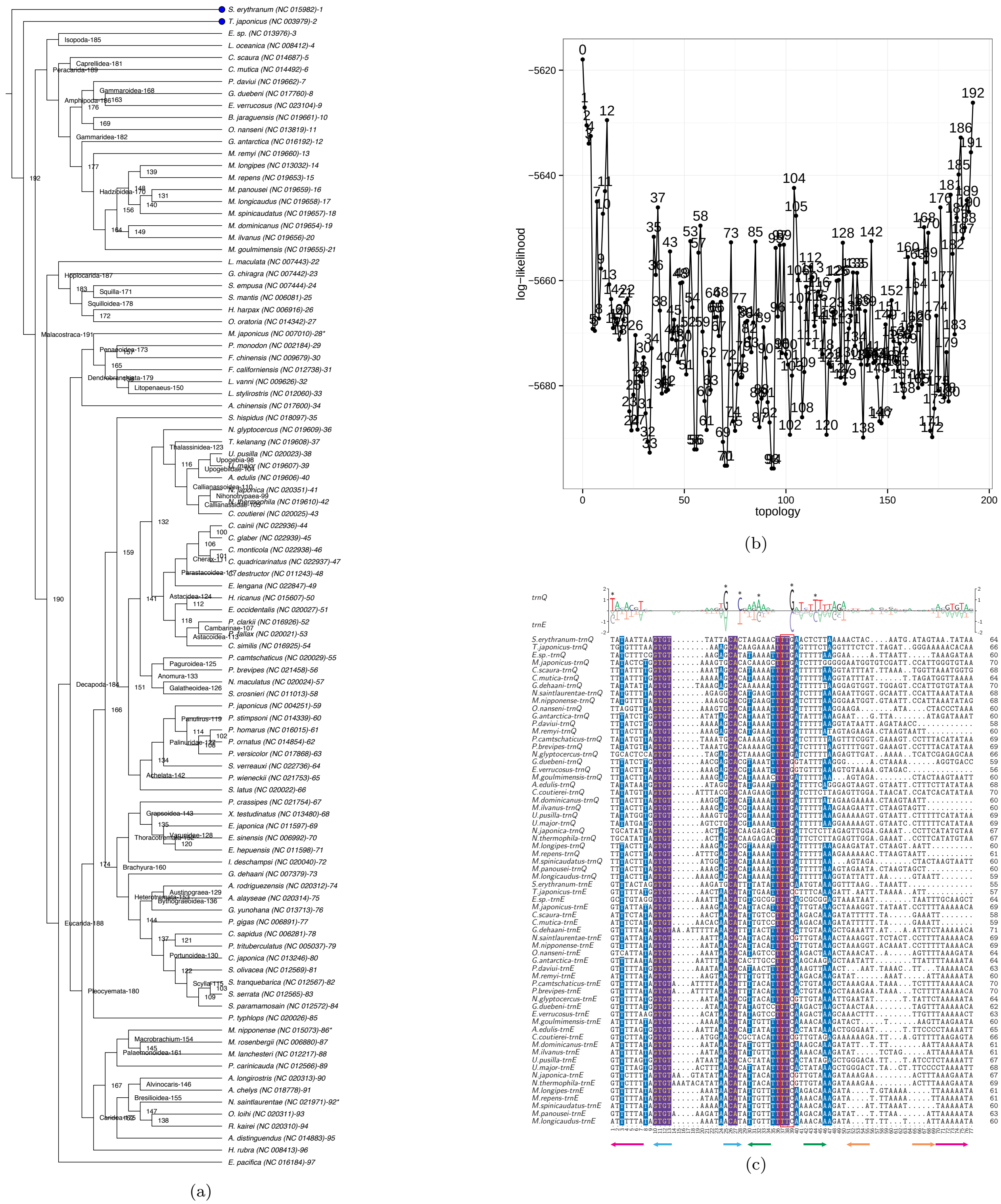

Figure 11: See Section 13

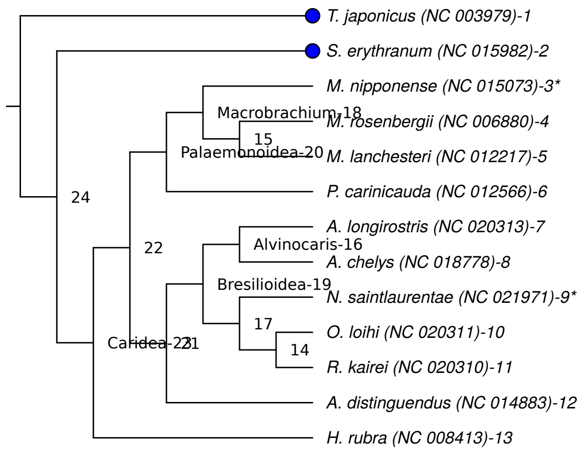

(a)

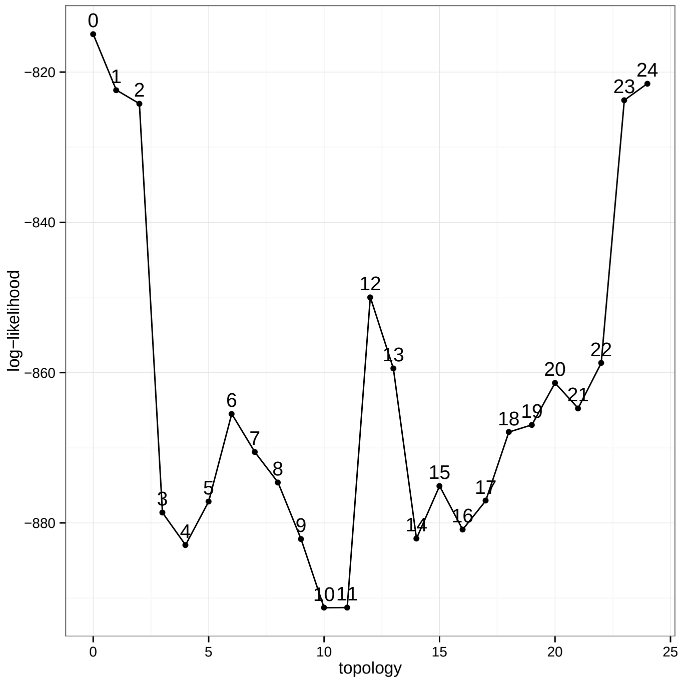

(b)

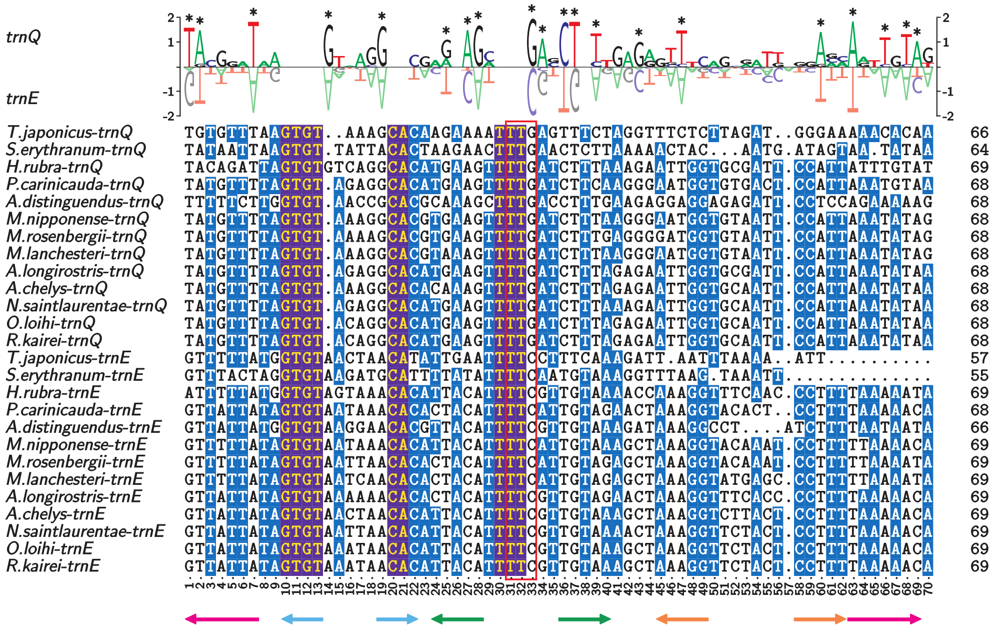

(c)

Figure 12: See Section 13

9.7 Mean Pairwise Similarities

	trnA	trnC	trnD	trnE	trnF	trnG	trnH	trnI	trnK	trnL1	trnL2	trnM	trnN	trnP	trnQ	trnR	trnS1	trnS2	trnT	trnV	trnW	trnY
trnA		6.27	**17.32**	**18.39**	4.66	**15.18**	**14.40**	4.53	0.30	-0.31	-2.99	**11.13**	4.09	**18.21**	5.98	**15.55**	-8.81	9.99	12.33	**14.86**	9.86	3.79
trnC	6.27		7.71	6.82	5.01	5.66	7.57	1.43	-1.01	-1.71	-2.77	-0.56	-1.86	-0.70	0.44	2.11	-6.75	0.42	5.75	-3.67	1.83	7.34
trnD	**17.32**	7.71		**14.85**	4.09	**14.46**	**16.39**	8.47	3.54	3.44	1.04	6.39	6.73	**11.65**	9.31	9.39	-4.19	4.24	**10.21**	6.11	**13.89**	8.28
trnE	**18.39**	6.82	**14.85**		2.74	**22.30**	**14.37**	3.03	3.10	**10.78**	3.82	6.28	-1.68	11.24	8.47	10.51	-8.98	0.63	**14.45**	3.15	6.48	7.02
trnF	4.66	5.01	4.09	2.74		2.17	**14.29**	2.62	2.00	1.88	3.37	3.86	-4.81	3.42	-5.49	1.39	-9.37	-4.45	9.87	6.65	3.65	6.76
trnG	**15.18**	5.66	**14.46**	**22.30**	2.17		7.95	-4.59	0.52	**12.57**	2.71	3.58	-3.20	2.76	3.59	**15.73**	-6.30	1.39	**11.45**	5.71	**15.15**	-0.42
trnH	**14.40**	7.57	**16.39**	**14.37**	**14.29**	7.95		**10.60**	2.73	5.80	6.05	**10.69**	-1.27	**16.13**	6.47	**15.39**	-5.55	1.32	**14.68**	5.63	**11.85**	**15.54**
trnI	4.53	1.43	8.47	3.03	2.62	-4.59	**10.60**		2.80	2.17	8.67	3.96	2.61	3.41	-2.79	1.42	-5.74	4.78	6.92	1.50	1.46	**14.46**
trnK	0.30	-1.01	3.54	3.10	2.00	0.52	2.73	2.80		6.17	3.10	2.46	**10.09**	5.22	-1.38	-0.22	-7.80	2.00	**14.16**	3.71	2.26	5.57
trnL1	-0.31	-1.71	3.44	**10.78**	1.88	12.57	5.80	2.17	6.17		**15.19**	4.94	-0.77	1.84	2.75	6.24	-8.56	1.07	**12.85**	3.00	5.66	4.55
trnL2	-2.99	-2.77	1.04	3.82	3.37	2.71	6.05	8.67	3.10	**15.19**		6.53	-2.65	-0.00	6.69	2.12	-5.41	**11.10**	5.52	1.28	4.35	**20.16**
trnM	**11.13**	-0.56	6.39	6.28	3.86	3.58	**10.69**	3.96	2.46	4.94	6.53		-0.72	7.37	0.99	**11.05**	-5.47	5.30	**10.20**	6.80	9.51	8.26
trnN	4.09	-1.86	6.73	-1.68	-4.81	-3.20	-1.27	2.61	**10.09**	-0.77	-2.65	-0.72		-1.50	5.23	-4.30	-6.40	8.47	4.20	-0.11	-3.99	3.88
trnP	**18.21**	-0.70	**11.65**	**11.24**	3.42	2.76	**16.13**	3.41	5.22	1.84	-0.00	7.37	-1.50		13.86	**12.40**	-7.20	7.63	**12.69**	8.04	**13.45**	6.61
trnQ	5.98	0.44	9.31	8.47	-5.49	3.59	6.47	-2.79	-1.38	2.75	6.69	0.99	5.23	**13.86**		6.95	-6.01	13.94	0.94	2.18	7.35	7.35
trnR	**15.55**	2.11	9.39	**10.51**	1.39	**15.73**	**15.39**	1.42	-0.22	6.24	2.12	**11.05**	-4.30	12.40	6.95		-4.63	3.78	5.65	9.87	**11.40**	6.71
trnS1	-8.81	-6.75	-4.19	-8.98	-9.37	-6.30	-5.55	-5.74	-7.80	-8.56	-5.41	-5.47	-6.40	-7.20	-6.01	-4.63		-3.52	-7.01	-7.87	-3.25	-5.80
trnS2	9.99	0.42	4.24	0.63	-4.45	1.39	1.32	4.78	2.00	1.07	**11.10**	5.30	8.47	7.63	**13.94**	3.78	-3.52		8.20	3.08	8.14	**14.15**
trnT	**12.33**	5.75	**10.21**	**14.45**	9.87	**11.45**	**14.68**	6.92	**14.16**	**12.85**	5.52	**10.20**	4.20	**12.69**	0.94	5.65	-7.01	8.20		4.06	**10.32**	6.72
trnV	**14.86**	-3.67	6.11	3.15	6.65	5.71	5.63	1.50	3.71	3.00	1.28	6.80	-0.11	8.04	2.18	9.87	-7.87	3.08	4.06		6.31	2.23
trnW	9.86	1.83	**13.89**	6.48	3.65	**15.15**	**11.85**	1.46	2.26	5.66	4.35	9.51	-3.99	**13.45**	7.35	**11.40**	-3.25	8.14	**10.32**	6.31		5.20
trnY	3.79	7.34	8.28	7.02	6.76	-0.42	**15.54**	**14.46**	5.57	4.55	**20.16**	8.26	3.88	6.61	7.35	6.71	-5.80	**14.15**	6.72	2.23	5.20	

Average bitscores between all pairs of tRNAs from Metazoa, i.e., $S(Y_i|X_j) : X \neq Y$; bold values indicate a mean value $>$ 10.

10 Porifera

10.1 trnS2(uga)⇝trnY(gua)

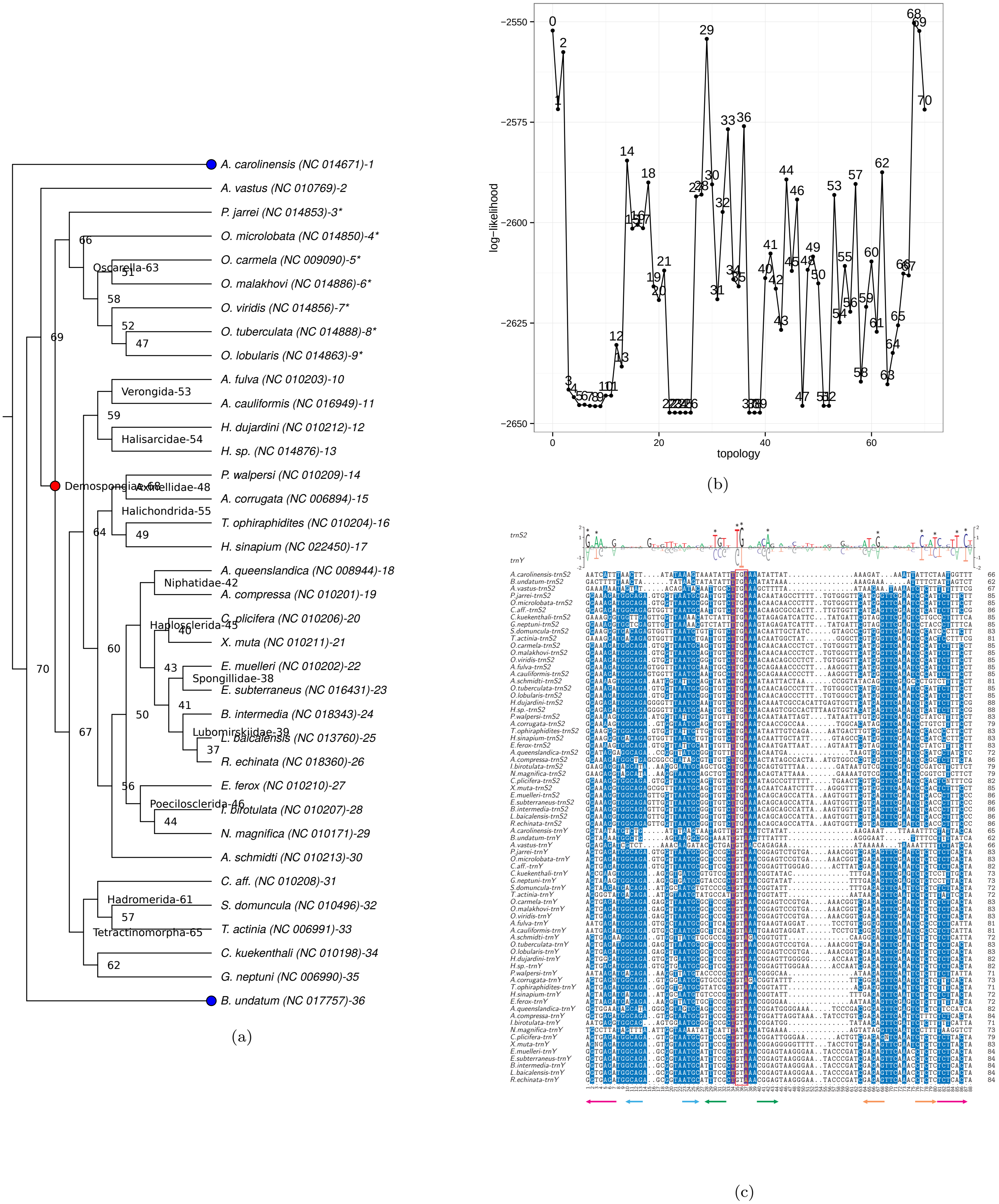

Figure 13: See Section 13

10.2 trnT(ugu)⇝trnR(ucu)

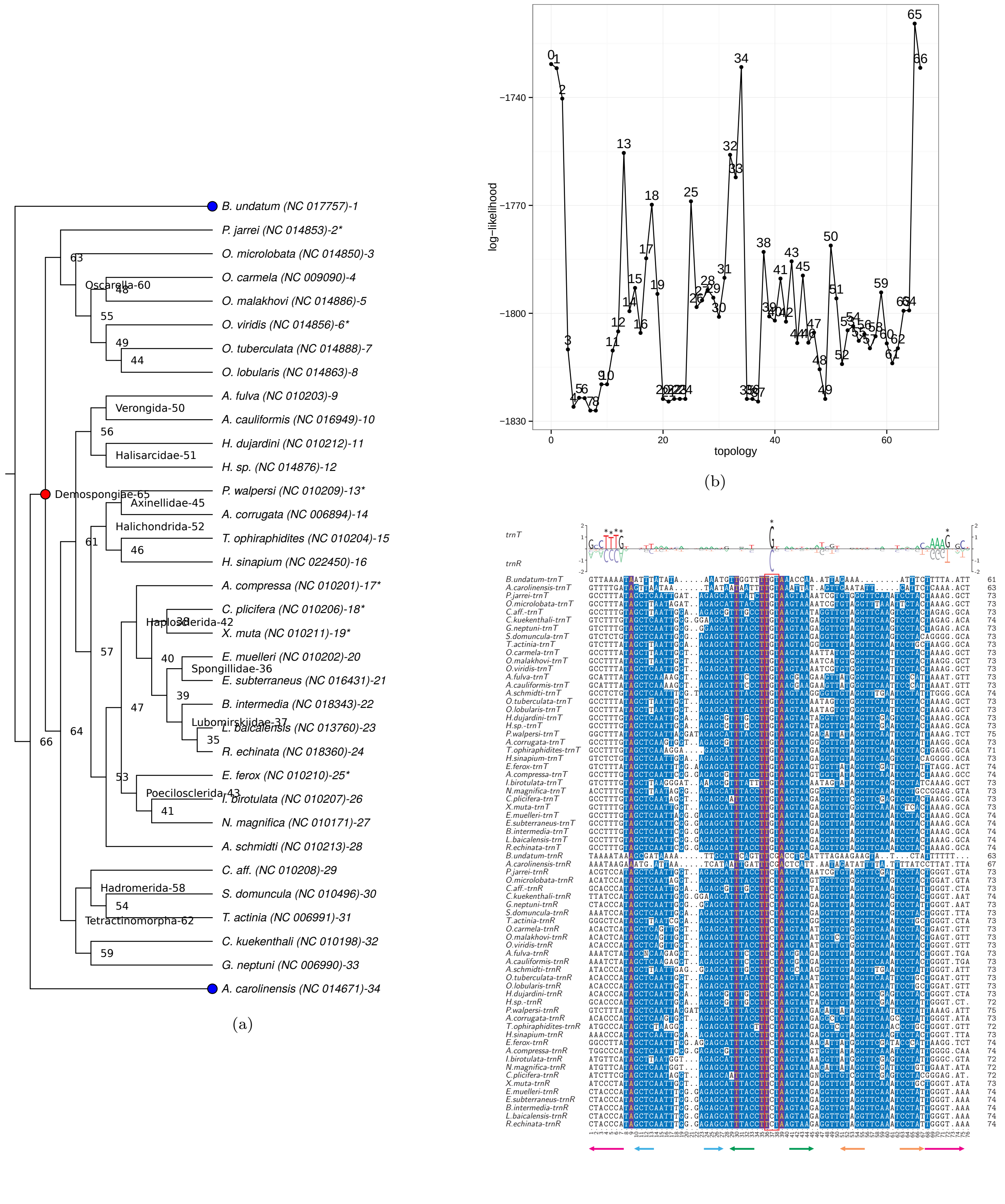

(a)

(b)

(c)

Figure 14: See Section 13

10.3 trnT(ugu)⇝trnR(ucg)

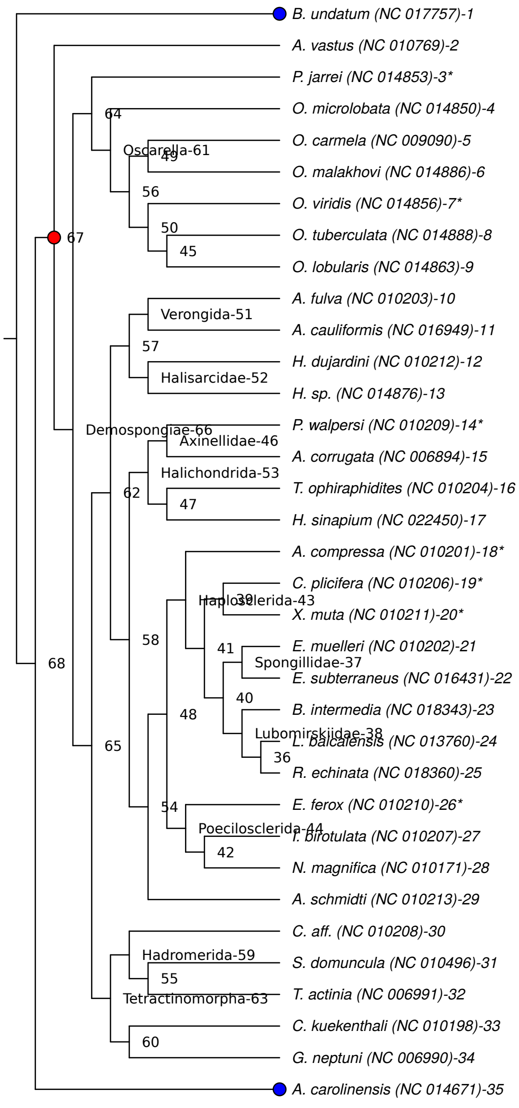

(a)

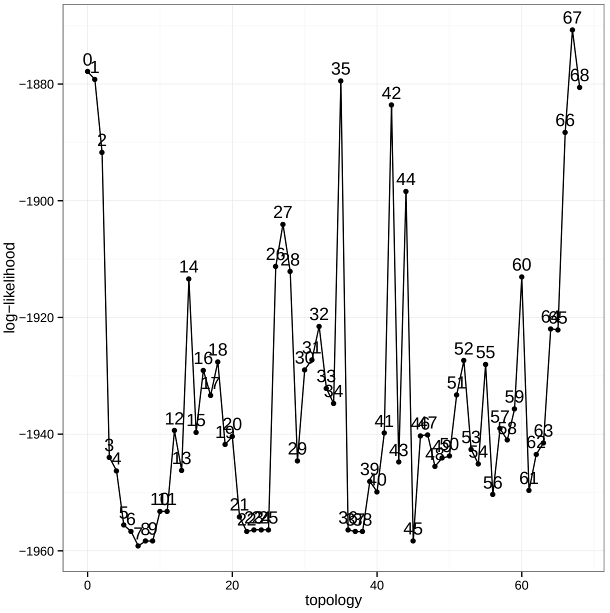

(b)

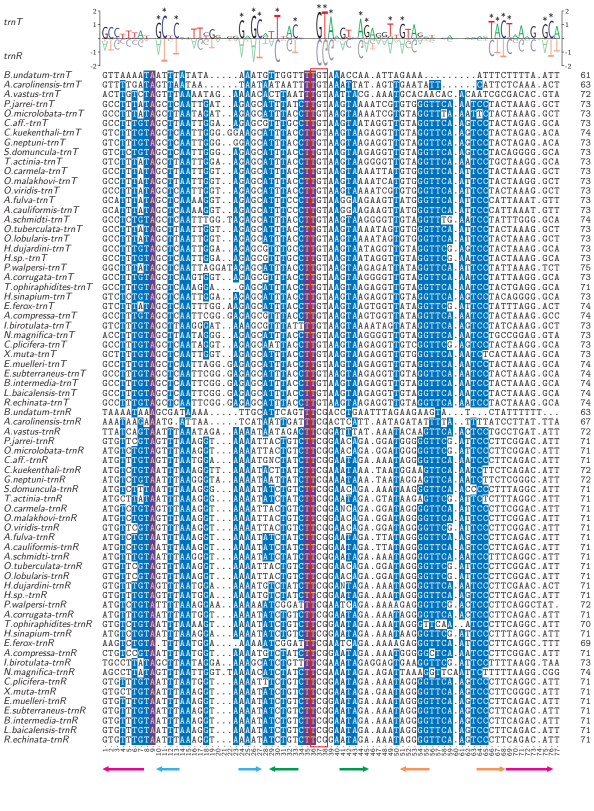

(c)

Figure 15: See Section 13

10.4 trnK(uuu)⇝trnN(ugu)

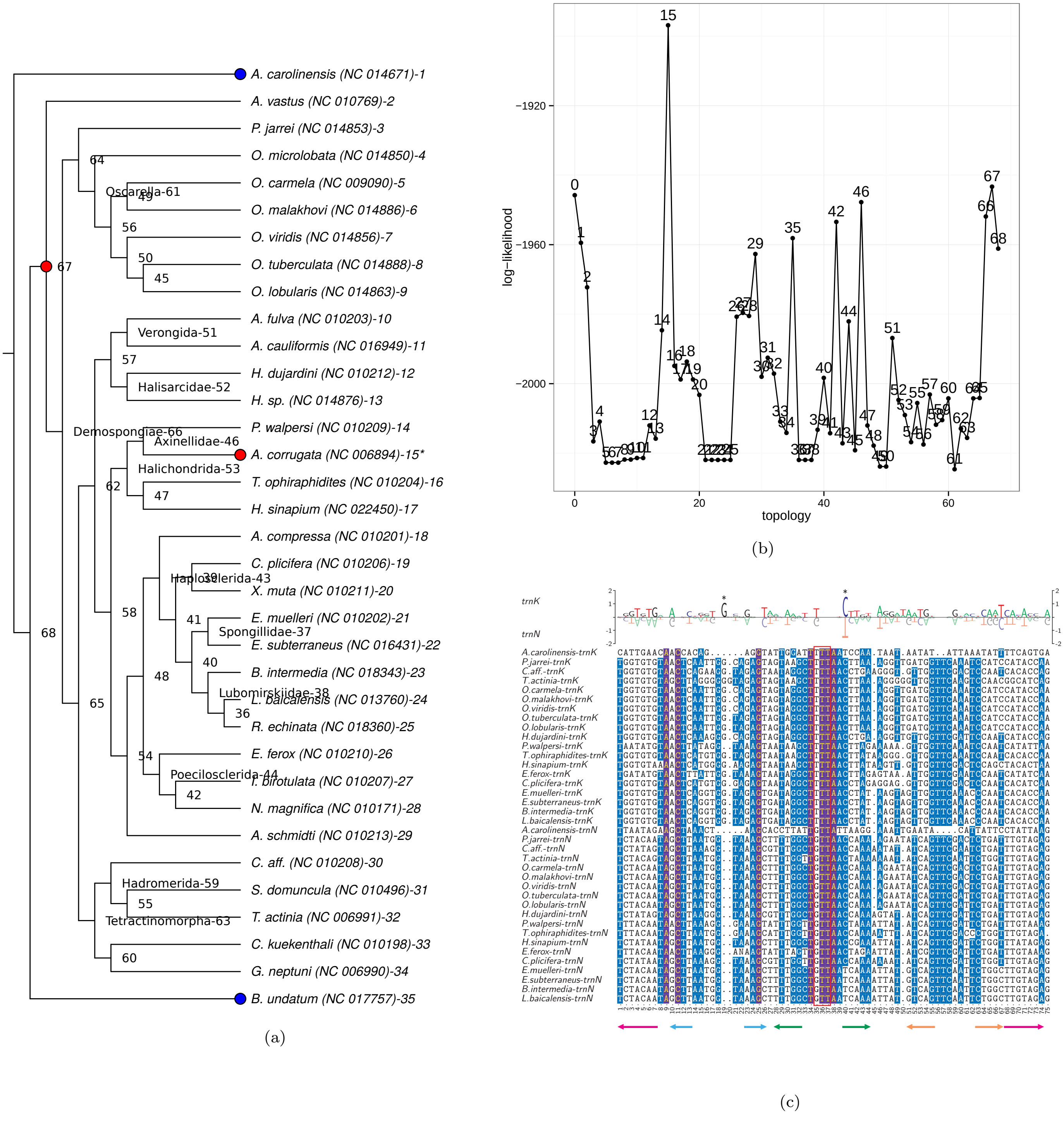

Figure 16: See Section 13

10.5 trnK(uuu)⇝trnC(gca)

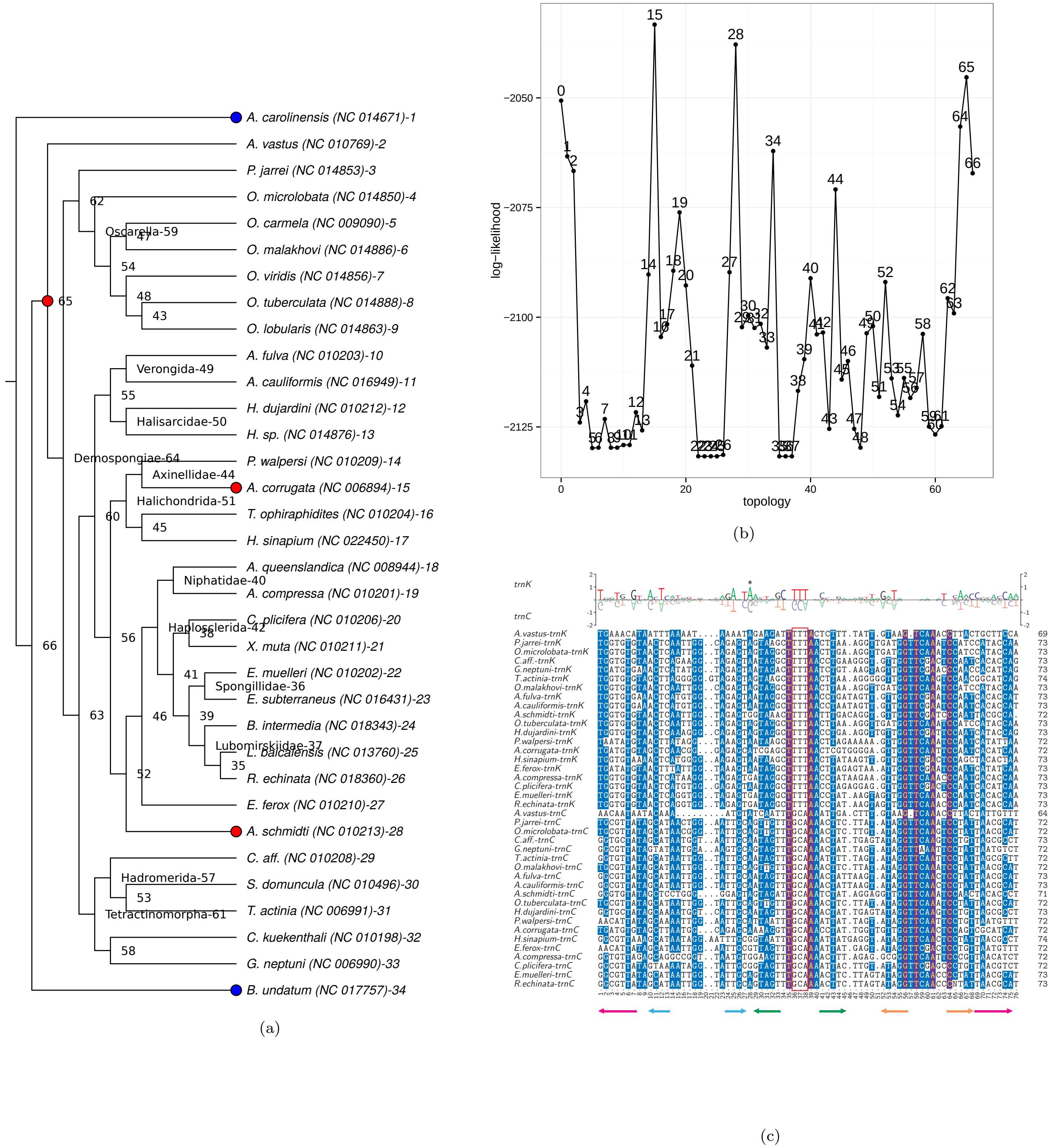

Figure 17: See Section 13

10.6 trnT(ugu)⇝trnV(uac)

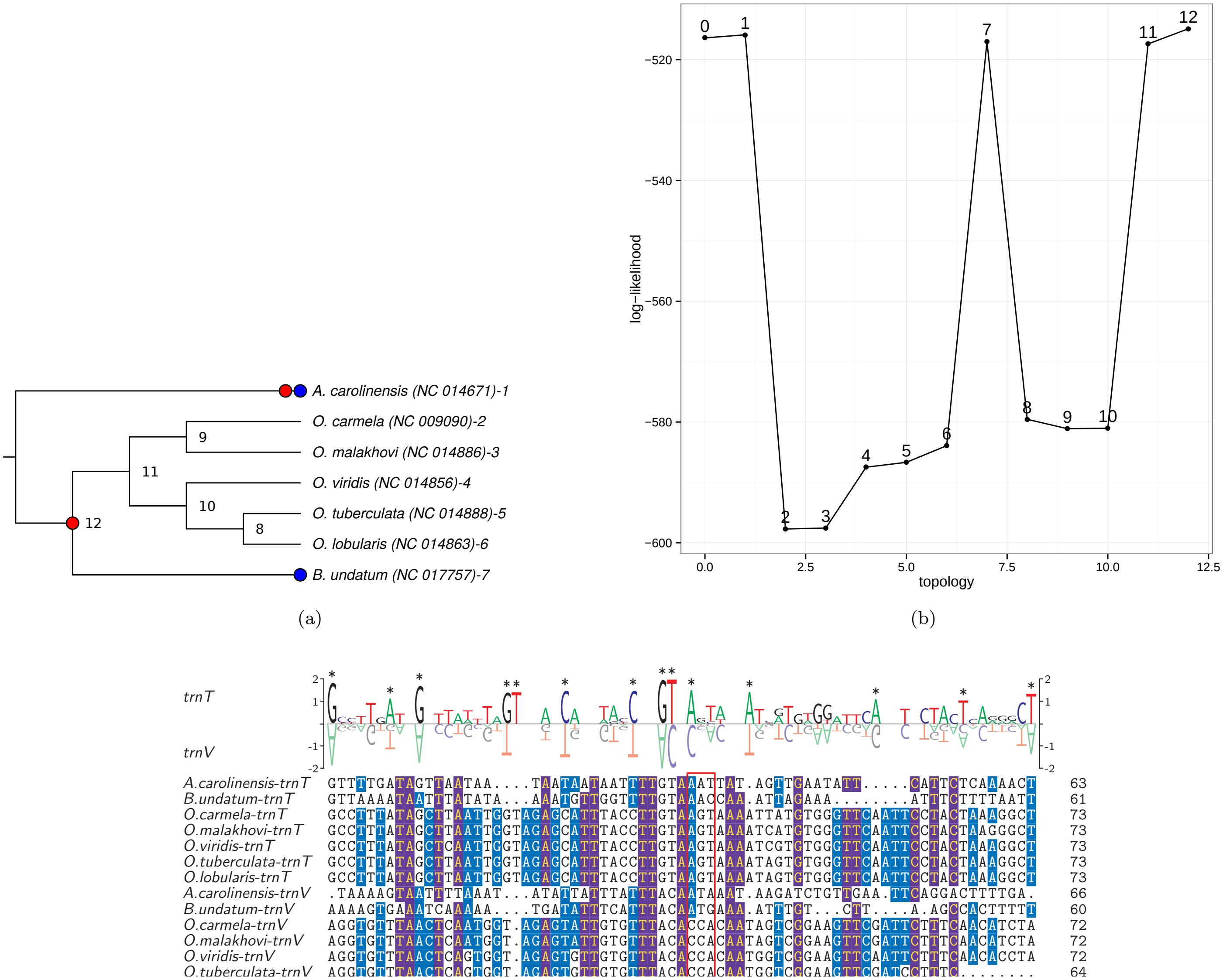

(a) (b)

(c)

Figure 18: See Section 13

10.7 trnV(uac)⇝trnT(ugu)

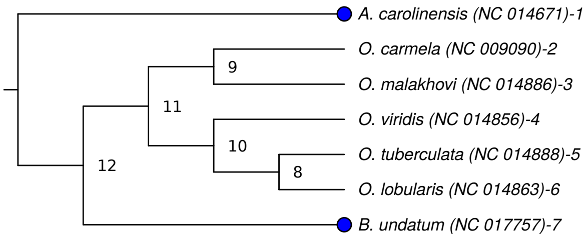

(a)

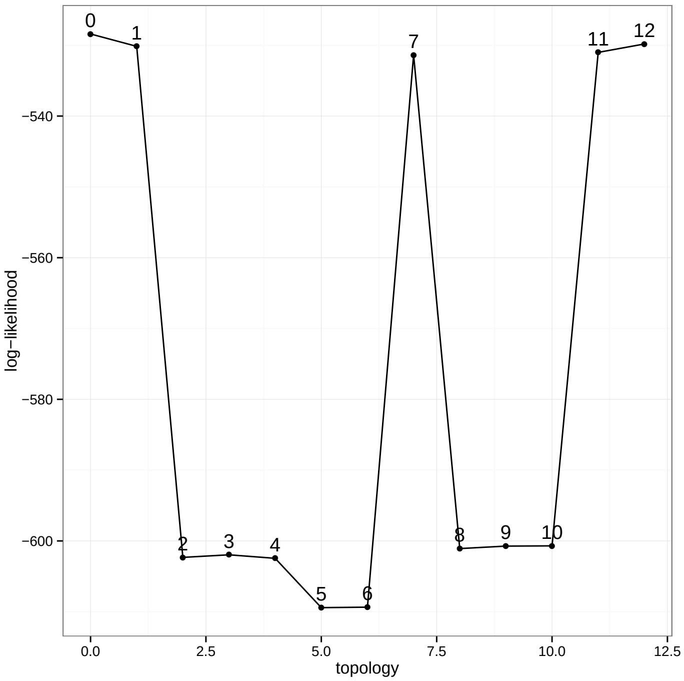

(b)

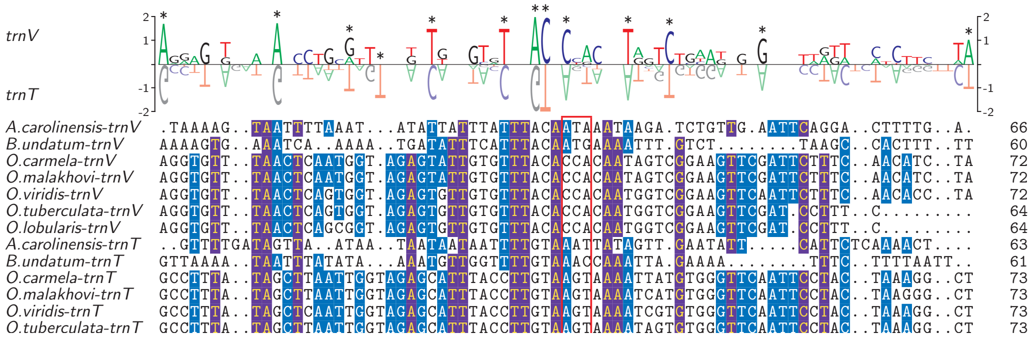

(c)

Figure 19: See Section 13

11 Leucine remolding

11.1 Eumalacostraca

The L2(UAA)⇝L1(UAG) have been analyzed for all Eumalacostraca, since the Isopoda and Pleocyemata contained candidates: the isopod *Eophreatoicus sp.*, both Paguroidea, and six of the eight Thalassinidea. Additionally, two L1(UAG)⇝L2(UAA) remolding candidates are found in Pleocyemata: *Corallianassa coutierei* (Thalassinidea) and *Geothelphusa dehaani* (Brachyura), see Figure 20 and 21. The `MLRD` analysis revealed several nodes with clearly increased log-likelihood indicating parallel remolding events, i.e., i) the Isopoda and *Eophreatoicus sp.* see [9], ii) Thalassinidea and many of its descendants see [10], iii) Paguroidea, iv) *Stenopus hispidus* see [11], and v) *Euphausia pacifica.*

Of particular interest are the cases detected in the Thalassinidea. The only mitogenome within the Thalassinidea that neither has an L2(UAA)⇝L1(UAG) nor an L1(UAG)⇝L2(UAA) candidate is *Thalassina kelanang.* It exhibits the ancestral pancrustacean gene order [10], i.e., *trnL2* between *cox1* and *cox2* and *trnL1* next to *nad1* which is well conserved in Eumalacostraca, i.e., in 78.1 %. This supports that no (recent) remolding of the leucine genes happened in *T. kelanang.* Based on the increased log-likelihood values for several nodes within the Thalassinidea and the nearly equal sequences of *trnL1* and *trnL2* for large parts of the alignment, e.g., the anticodon loop and the acceptor stem, our data suggests one L2(UAA)⇝L1(UAG) remolding ancestral to Callianassoidea and *Neaxius glyptocercus* (Axioidea). The multiplicity of nodes with increased log-likelihood might also be interpreted as additional independent L2(UAA)⇝L1(UAG) or L1(UAG)⇝L2(UAA) remolding events ancestral to Upogebiidae, Callianassidae, or Callianassoidea. This is supported by several columns in the alignments that are identical for *trnL1* and *trnL2* and specific for Upogebiidae and Callianassidae, respectively. Two separate remolding events have been suggested in [10].

A scenario involving multiple remolding events is also supported by gene orders. While the unremolded *T. kelanang* exhibits the ancestral position of *trnL1* (adjacent to *nad1*) the remaining Thalassinidea have both leucine tRNAs adjacent between *cox1* and *cox2*: in Upogebiidae in the configuration *cox1 trnL2 trnL1 cox2* and in the remaining in the configuration *cox1 trnL1 trnL2 cox2.* Since the difference in gene order can be explained also by a transposition, the question if recurring events happened needs to be analyzed with an improved species sampling and a more reliable phylogeny. Interestingly, the *cox1 trnL2 trnL1 cox2* configuration is also found in the closely related *Stenopus hispidus* that is also a candidate for L2(UAA)⇝L1(UAG) remolding. Also in *Eophreatoicus sp.* the leucines ended up next to *cox1* (*trnQ trnL2 trnL1 cox1*).

An L2(UAA)⇝L1(UAG) remolding for the Anomura (which include the Paguroidea) has been suggested [12] based on a maximum likelihood phylogenetic reconstruction with weak bootstrap support. Unfortunately, due to the use of RefSeq we use a disparate set of species here (we lack Hippoidea and include additional different Paguroidea). Due to our species sampling which included the Galatheoidea which do not possess remolded *trnL* according to our results the remolding likely happened after the split of the Anomura and before the Paguroidea.

Furthermore, a slightly increased log-likelihood is observed for the Isopoda and the node separating the crustacean outgroup and the Eumalacostraca. Both cases might be an artifact caused by: i) not adequately sampled unremolded Isopoda (*Ligia oceanica*, respectively outgroup) which can not reduce the log-likelihood sufficiently when `MLRD` groups them together with the remolded *Eophreatoicus sp.* which has a very high log-likelihood on its own and ii) the high similarity of the *trnL* due to ancestral events [13, 12] complicate the detection of recurring events. A slightly increased likelihood is also observed for *G. dehaani* probably caused by the L1(UAG)⇝L2(UAA) remolding [14], which is correctly reflected in the candidate set. Furthermore, in the `MLRD` analysis for L1(UAG)⇝L2(UAA) the highest peak is *G. dehaani.* We remark that the log-likelihood values differ for the two data sets since the alignments have been constructed using the donor tRNA models that are different for the two cases. Note that the L1(UAG)⇝L2(UAA) remolding events presented in [14] and here are an update to [12] who mentioned that there are no known cases of L1(UAG)⇝L2(UAA) remoldings except for one case in human lung carcinoma.

11.1.1 Eumalacostraca trnL2(uaa)⇝trnL1(uag)

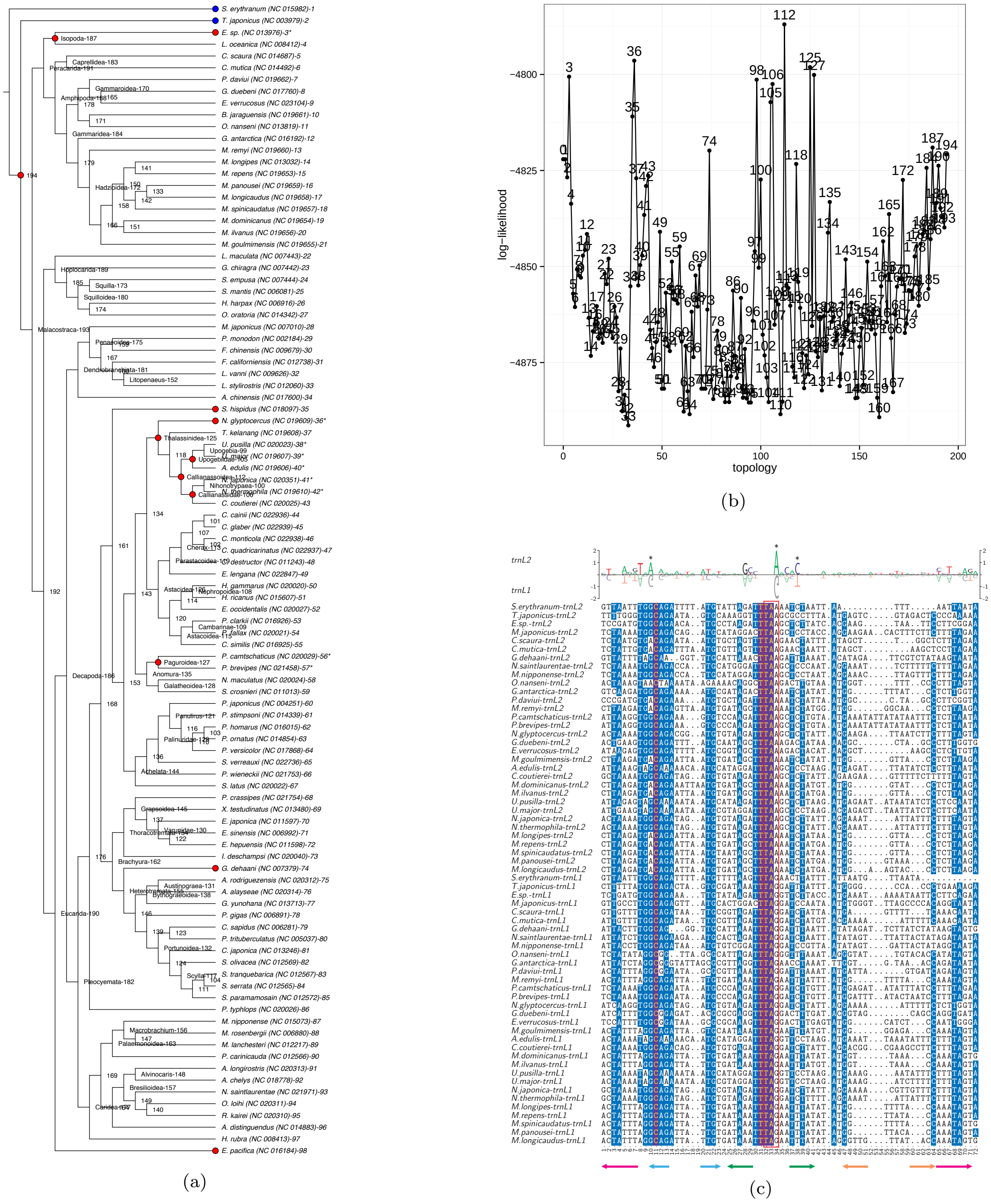

Figure 20: See Section 13

11.1.2 Eumalacostraca trnL1(uag)$\rightsquigarrow$trnL2(uaa)

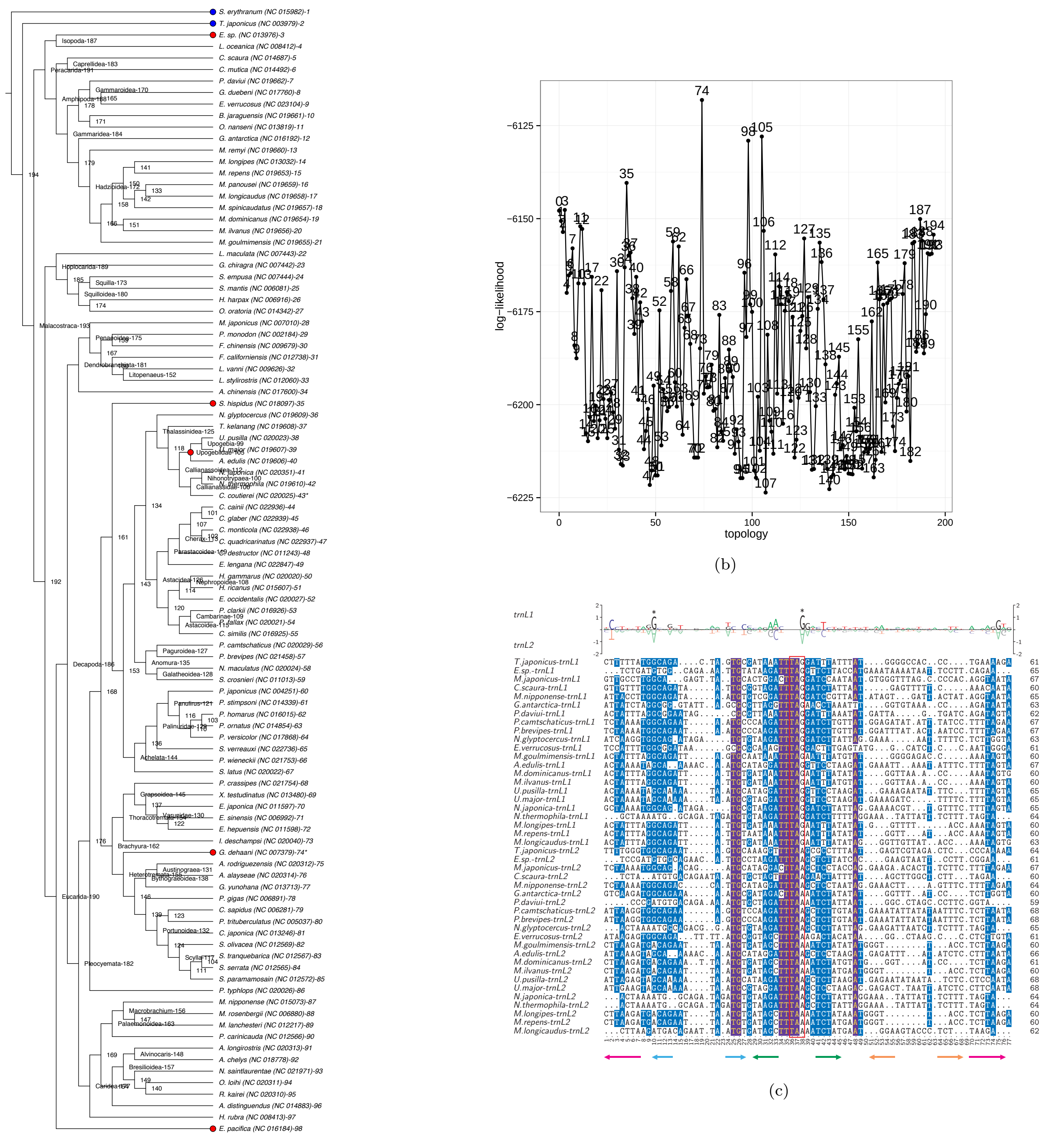

(a) (b) (c)

Figure 21: See Section 13

11.2 Ambulacraria trnL2(uaa)⇝trnL1(uag)

An L2(UAA)⇝L1(UAG) remolding at the common ancestor of echinoderms and hemichordates has been suggested based on phylogenetic reconstruction of tRNA genes from selected species of these groups [13]. Further support comes from the high similarity of the leucine tRNAs [12] and a putative 6 bp duplication remnant at the 5' end of *nad5*, which is adjacent to *nad5* in most deuterostome mitogenomes, i.e., for 97.3 % of the chordates, see [15].

The candidate set $\mathcal{R}$ contains three types of remolding events in Ambulacraria: i) 11 L2(UAA)⇝L1(UAG) remolding within Echinoderms, ii) 1 E(UUC)⇝A(UGC) remolding for *Echinocardium cordatum*, and iii) 1 S2(UGA)⇝L2(UAA) remolding for *Rhabdopleura compacta* (Hemichordata).

The found L2(UAA)⇝L1(UAG) remoldings are consistent with the assumption of a remolding ancestral to the Ambulacraria as in [13], but no L2(UAA)⇝L1(UAG) remolding for hemichordates has been found in our candidate set. The high similarity observed in the alignment supports that the leucine tRNAs underwent a remolding, i.e., 41 columns are preserved in >50 % of the sequences and there are only three tRNA specific columns which included the mutation of the anticodon. Additionally, `MLRD` showed increased log-likelihood values where the maximum is found for the Ambulacraria. However, the alignment shows that the *trnL1* of the outgroups *A. aspersa* and *X. bocki* are also quite similar to the other sequences and a slightly increased log-likelihood value has been detected for *X. bocki*. There are two possibilities to explain this: i) there was a separate individual $L2(UAA) \rightsquigarrow L1(UAG)$ remolding for *X. bocki*, or ii) the $L2(UAA) \rightsquigarrow L1(UAG)$ is a more ancestral event. A remolding that is ancestral to Ambulacraria is also supported by the gene order. Within echinoderms, both tRNAs are involved in well preserved adjacencies. With few exceptions *trnL1* is adjacent to *trnA* or *trnN* (25/36); and *trnL2* is adjacent to *trnG* or *nad1* (32/36). However, within hemichordates this adjacency seems to be lost where *trnL2* and *trnL1* are adjacent in both *Balanoglossus* species and in different positions in both other hemichordate species present in RefSeq. Additionally, a separate remolding for *X. bocki* is supported by the gene order of this species where *trnL2* and *trnL1* are adjacent.

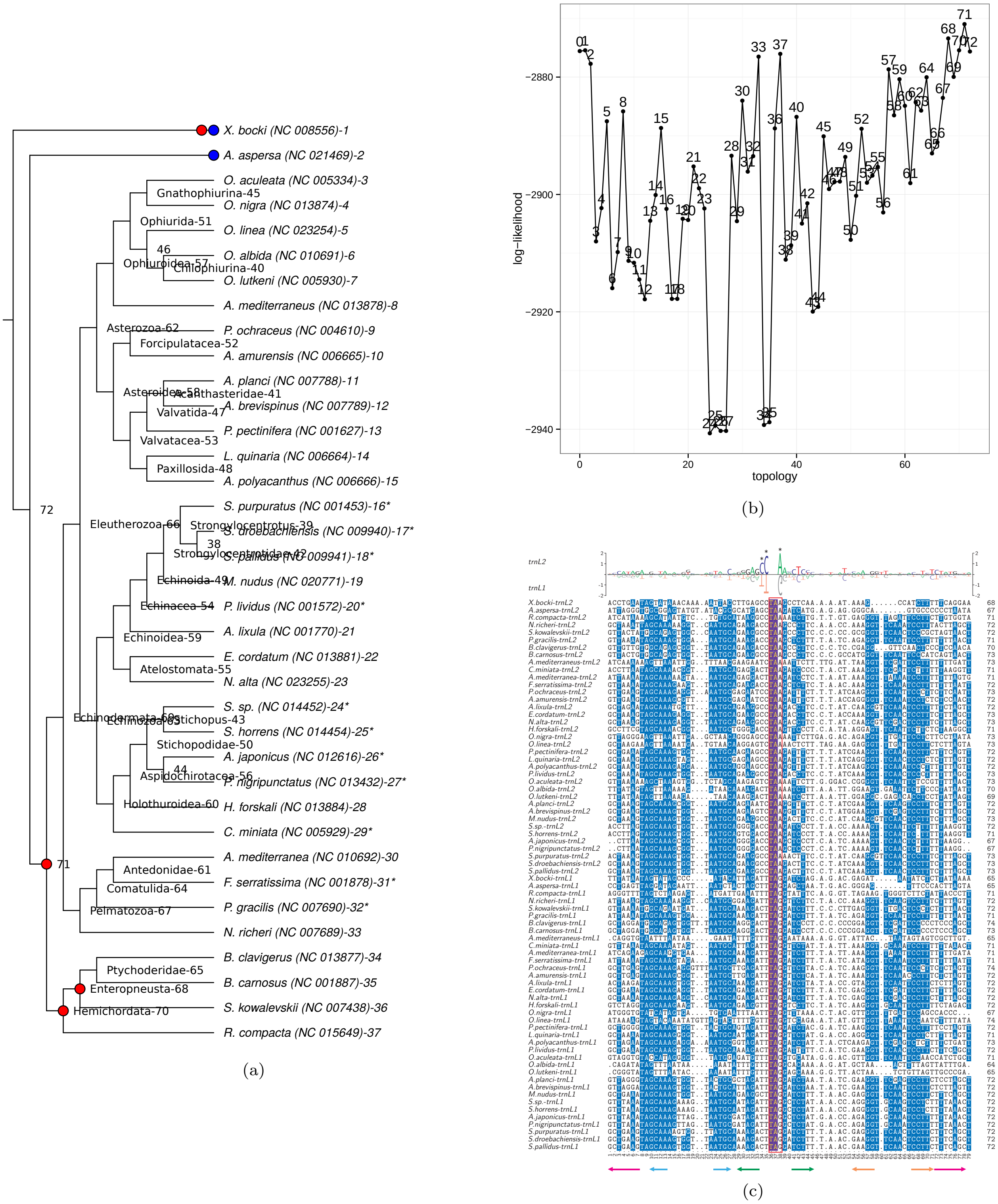

Figure 22: See Section 13

11.3 Mollusca

Two independent remolding events in Mollusca, i.e., one L2(UAA)$\rightsquigarrow$L1(UAG) remolding within the mitogenome of *Littorina saxatilis* and another L1(UAG)$\rightsquigarrow$L2(UAA) remolding at the common ancestor of Bivalves has been suggested [13]. The findings of [13] were based on rather weakly supported nodes in a phylogenetic reconstruction using the available small species sample. Independent leucine remoldings in caenogastropod lineages have been suggested in [12].

The candidate set $\mathcal{R}$ contains only one L1(UAG)$\rightsquigarrow$L2(UAA) remolding candidate within Bivalvia, i.e., *Fulvia mutica*. In order to check why no L2$\rightsquigarrow$L1 remolding candidates are found for Mollusca, we determined for one of the Littorinimorpha (*Dendropoma maximum*) which of the criteria are violated: for the test L1$\rightsquigarrow$L2 already the 1st test fails, i.e., the tRNAs are not exceptionally similar, and for the L2$\rightsquigarrow$L1 the 3rd test fails, i.e., the test for the direction. The set $\mathcal{P}$ comprised 105 *trnL1 trnL2* pairs for the 171 Mollusca in RefSeq. The alignment shows that the leucine tRNAs of Mollusca are highly similar. These cases might be false negatives of `SRD`. This is also supported by the `MLRD` and gene order analysis.

An `MLRD` analysis for L2(UAA)$\rightsquigarrow$L1(UAG) and L1(UAG)$\rightsquigarrow$L2(UAA) remoldings in Mollusca showed multiple increased log-likelihood values. In both analyses, among the highest log-likelihood values are the ones for the Vermetoidea family and node 267 that are included in the order Littorinimorpha. In the L2(UAA)$\rightsquigarrow$L1(UAG) analysis the log-likelihood values for Vermetoidea is on the 1st rank. Whereas it is on 3rd for the L1(UAG)$\rightsquigarrow$L2(UAA) analysis. The 2nd highest log-likelihood value in the L1(UAG)$\rightsquigarrow$L2(UAA) analysis is found for the root of the Mollusca. On the one hand, increased log-likelihood values are found for some Bivalvia groups in both analyses (i.e., Mytilus, Lucinoidea, Cardioidea, Vermetoidea, Mollusca, node 267, and many other leaf nodes). On the other hand, increased log-likelihood values are found separately for each of the separate analyses: i) Ostreoida, Heteroconchia, Veneroidea, Littorinimorpha, Truncatelloidea, nodes 328, 232, 218, and few other single species for L2(UAA)$\rightsquigarrow$L1(UAG), and ii) few single species for L1(UAG)$\rightsquigarrow$L2(UAA).

In mollusc gene arrangements the two leucine tRNAs are often adjacent (in 61 of the 171 genomes). Examples are found in all major groups with available mitogenomes, i.e., Gastropoda, Cephalopoda, Bivalvia, Polyplacophora, and Scaphopoda, except for the Aplacophora where only one mitogenome is in RefSeq. In most of these cases the adjacent leucine tRNAs are surrounded by *rrnL* and *nad1*. Some of these cases agree with the results of the `MLRD` analysis, e.g., *Hiatella arctica*, all four Mytilus, and 7 of 11 Littorinimorpha have adjacent leucine tRNAs. But many other examples with this adjacency, e.g., Cephalopoda and Neogastropoda, were not recovered in the `MLRD`. Note that the same configuration, i.e., *nad1 trnL2 trnL1 rrnL*, is also found in 35 non-mollusc gene orders, i.e., 21 Arthropoda (Myriapoda, Chelicerata, and Hexapoda), one brachiopod (Phoronopsis), 4 Nemertea, and even one Hemichordate (*Balanoglossus carnosus*). Hence, this adjacency was either formed multiple times independently or once early in the evolution of Bilateria. Assuming that the adjacency was created early (e.g., by a remolding event), more recent remoldings due to tandem duplications of a leucine tRNA have no consequence on the gene order in 50 % of the cases. Indeed, in Littorinimorpha and in one chelicerate (*Eremobates cf. palpisetulosus*) the leucines are found in opposite order (i.e., *rrnL trnL2 trnL1 nad1* instead of *rrnL trnL1 trnL2 nad1*).

Concluding, our data supports an ancestral L1(UAG)$\rightsquigarrow$L2(UAA). But whether there was one remolding at the root of the Mollusca and another one ancestral to Protostomia can not be decided based on this data, because Ecdysozoa were used as outgroup (see below). Our data suggest more recent remoldings but they are difficult to verify due to the high similarity of the already remolded leucine tRNAs.

A detailed analysis for L2(UAA)$\rightsquigarrow$L1(UAG) remoldings has been conducted for Mollusca. The `MLRD` analysis revealed several nodes with increased log-likelihood that indicate possible remolding events, i.e., Vermetoidea and Truncatelloidea which are subgroups of Littorinimorpha where [13] reported a similar remolding within the mitogenome of *L. saxatilis*. Additionally, the alignment support the conclusion and shows a high similarity between then *trnL1* and *trnL2* genes. However, one could except high similarity between *trnL1* and *trnL2* given the suggested remolding at the common ancestor of all protostomes [13]

Additionally, the L1(UAG)$\rightsquigarrow$L2(UAA) for all Mollusca have been analyzed. Surprisingly, several nodes with significantly increased log-likelihood values have been observed that indicate possible remolding events, i.e., Vermetoidea and many of its descendants, Lucinoidea, and the root of the Mollusca. This suggest a possible L1(UAG)$\rightsquigarrow$L2(UAA) remolding at the root of the Mollusca. No significant log-likelihood values have been detected for the two species reported in the study of [13] (i.e., *Mytilus edulis* and *Crassostrea gigas*). Consistent with the conclusion of an ancestral L1(UAG)$\rightsquigarrow$L2(UAA) remolding at the root of the Mollusca, the alignment shows that the *trnL1* of Mollusca are highly similar. However, one should not exclude the possibility that this similarity might be caused by a more ancestral L1(UAG)$\rightsquigarrow$L2(UAA) or L2(UAA)$\rightsquigarrow$L1(UAG) event.

The log-likelihood plots for both methods point out to an ancestral L1(UAG)$\rightsquigarrow$L2(UAA) remolding at the root of the Mollusca and another L2(UAA)$\rightsquigarrow$L1(UAG) at more basal groups (i.e., Vermetoidea and Truncatelloidea). This is because for the L1(UAG)$\rightsquigarrow$L2(UAA) reconstruction, the log-likelihood at the root of the molluscs shows significantly high value compared to the value yielded by the original topology. However, for L2(UAA)$\rightsquigarrow$L1(UAG) reconstruction, no significant likelihood value can be observed at the root of the molluscs.

11.3.1 trnL2(uaa)⇝trnL1(uag)

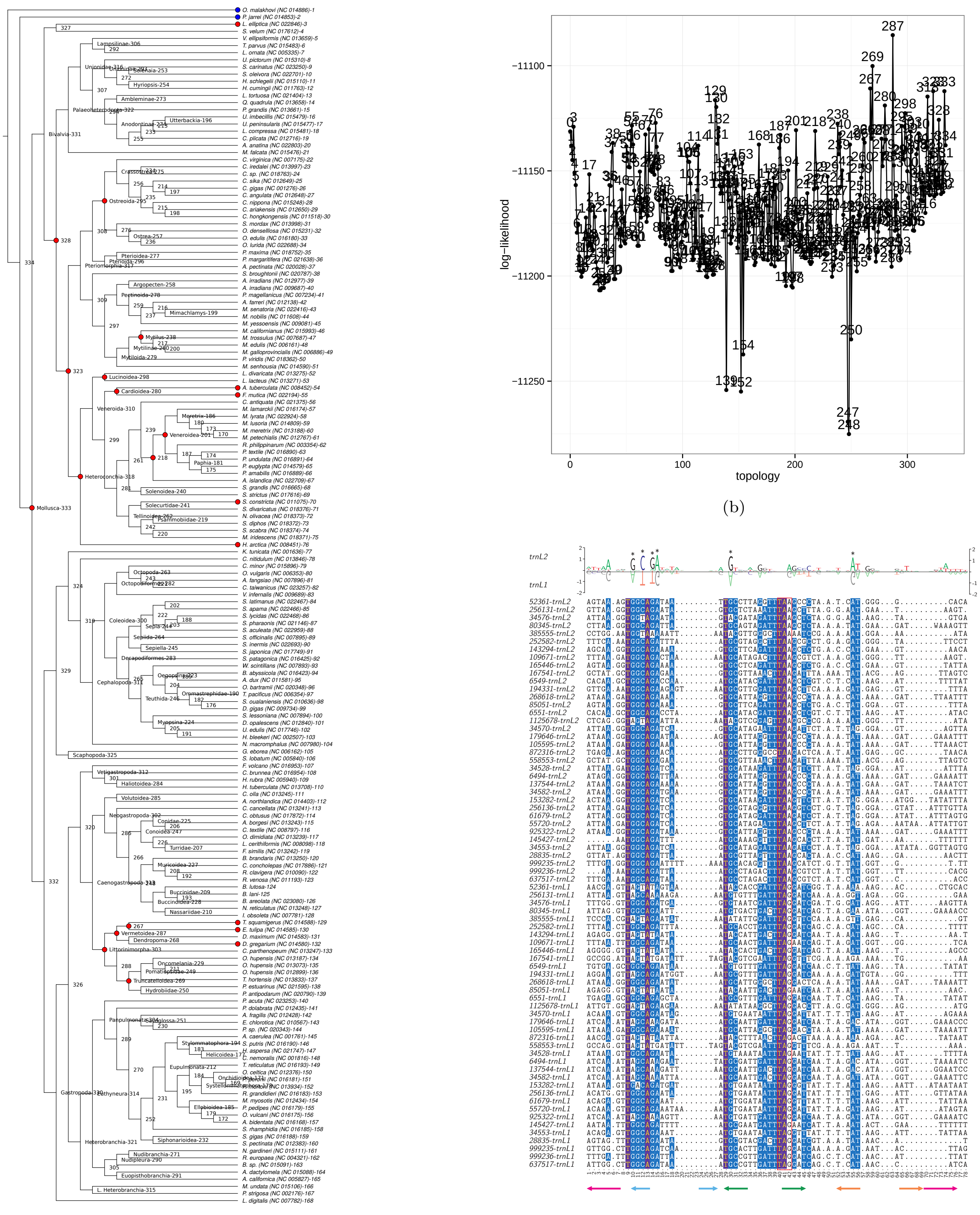

(a) (b) (c)

Figure 23: See Section 13. For this data set, the right part of the alignment contains five (blue) conserved columns but also 90% gaps and therefore have been omitted.

11.3.2 trnL1(uag)⇝trnL2(uaa)

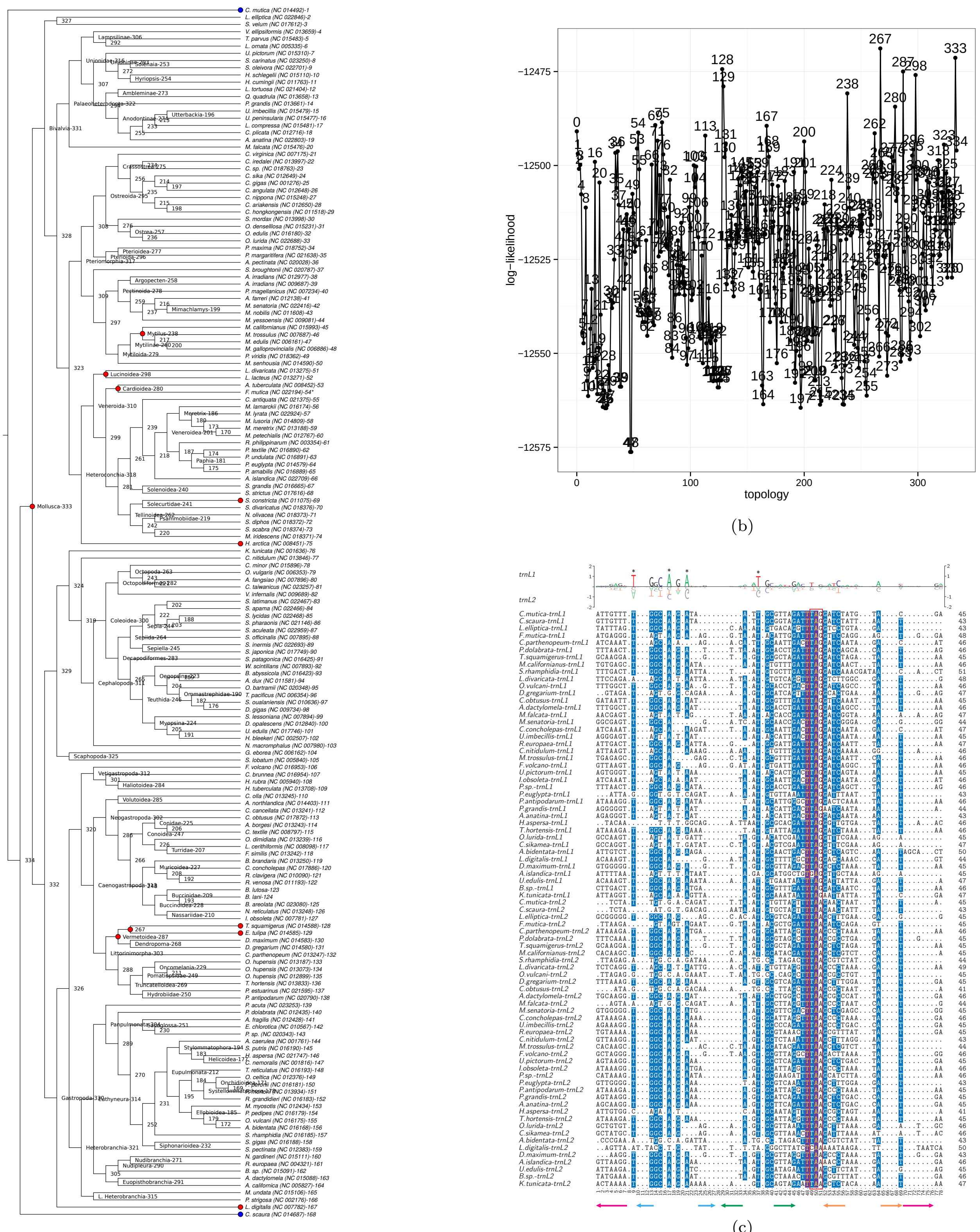

(a) (b) (c)

Figure 24: See Section 13. For this data set, the right part of the alignment contains two (blue) conserved columns but also 90% gaps and therefore have been omitted.

11.4 Metazoa

To test for the possibility of an ancestral Leucine remolding, we constructed a data set containing a set of species with balanced species sampling as described in Materials and Methods.

The `MLRD` analyses for L2(UAA)⇝L1(UAG) and L1(UAG)⇝L2(UAA) showed a nearly coinciding set of several nodes with increased log-likelihood values. The largest values are found for the Bilateria followed by the Protostomia. Further increased values are found for Protostomia+Platyhelminthes, Porifera, Deuterostomia, Eleutherozoa, a molluscan group, and several leaf nodes. Since `MLRD` is based on the assumption of a single remolding event an unequivocal interpretation of these nodes as positions of ancestral remoldings is not possible. This is in particular for neighboring nodes on the same path from a leaf to the root. But the consequence of the violation of the assumption might be less severe for independent events which might be the case for the Porifera. Note that Leucine remoldings in Porifera have been speculated in [16]. Furthermore, our data weakly points to a remolding at the root of the Porifera with a high similarity between both leucine tRNAs (see Supplement 11.5). The absence of a proper outgroup, i.e., unremolded *trnL* genes, is a further complication of this (and all earlier studies). Note that also for fungi it can not be assumed *a priori* that the leucine tRNAs have not been remolded since (other) remoldings have been also reported within fungal mitogenomes [17].

The adjacencies that are found nearly always (in 2332 of 2621) in Chordata are *nad5 trnL1 trnS1* and *nad1 trnL2 rrnL*. In Ecdysozoa *trnL1* took the place of *trnL2*, i.e., *nad1 trnL1 rrnL* is found in 471 of 755 cases, and *trnL2* is found at a different position, i.e., mostly between *cox1* and *cox2*. This supports an L2(UAA)⇝L1(UAG) remolding between the emergence of Bilateria and the Ecdysozoa. In some of the Ecdysozoa (24) and some of the Lophotrochozoa (57 of 217) both leucine tRNAs are found between *nad1* and *rrnL* which indicates further independent remolding events (consistent with our results for the Eumalacostraca) or the differential loss of a duplicate generated before the emergence of the lineages. In Ambulacraria gene orders the *trnL1* is found mostly between two different genes, i.e., *trnA* and *trnN*, and *trnL2* is adjacent to *nad1* and *trnG*. Which would support a further L2⇝L1 remolding. The adjacencies observed for the Porifera are *trnL1* adjacent to *nad5* in six cases (as in Chordata) and *trnL1* adjacent to *trnN* in five cases (as in Ambulacraria). The other gene that is adjacent to *trnL1* is *cob* in all these cases. Surprisingly, this adjacency is found also in 25 chordate mitogenomes. The *trnL2* gene is adjacent mostly to either *trnG* (as found for Ambulacraria) and *nad1* (found throughout Deuterostomia). This mixed pattern, the small species sample, and missing outgroup information, does not allow for an obvious interpretation. The observed adjacencies might be convergences.

Concluding, our data and gene order data supports multiple deep metazoan leucine remoldings, i.e., for the Protostomia, Ambulacraria, and Porifera. This is consistent with [12, 13, 16]. Furthermore multiple re-remoldings, in particular within Protostomia, are supported (see also results for Mollusca and Eumalacostraca). If further even deeper remolding events happened, as indicated by the results of `MLRD` and the high similarity of all metazoan leucine tRNAs (see Supplement 9.7), needs further refined analyses including fungal data.

11.4.1 trnL2(uaa)⇝trnL1(uag)

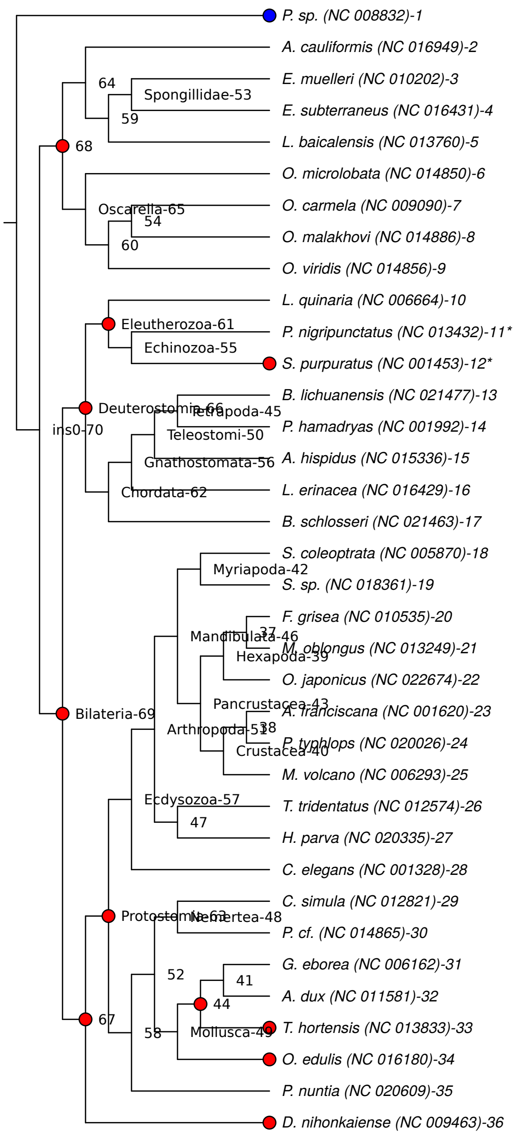

(a)

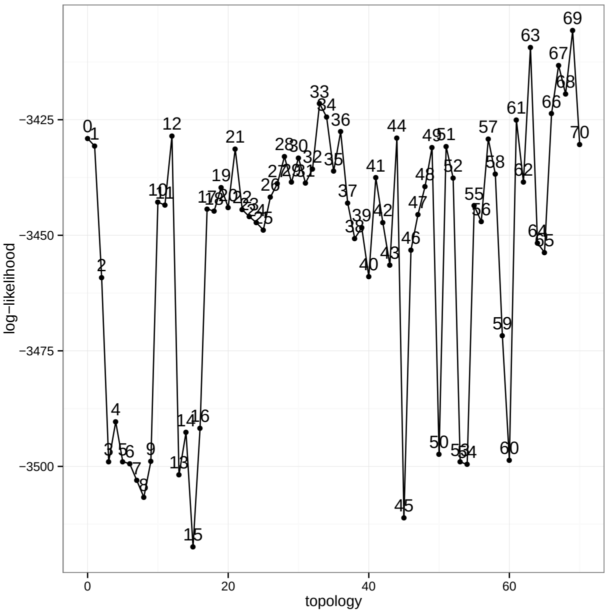

(b)

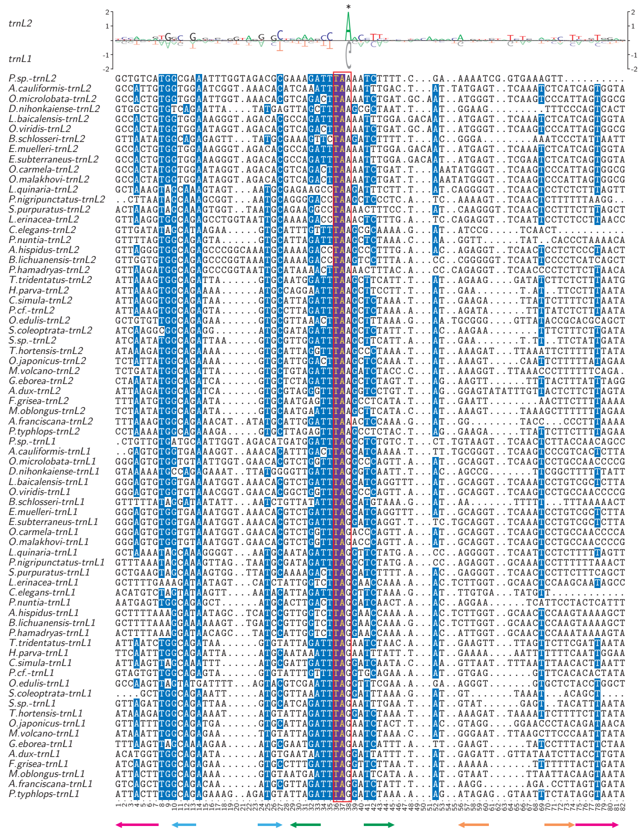

(c)

Figure 25: See Section 13

11.4.2 trnL1(uag)⇝trnL2(uaa)

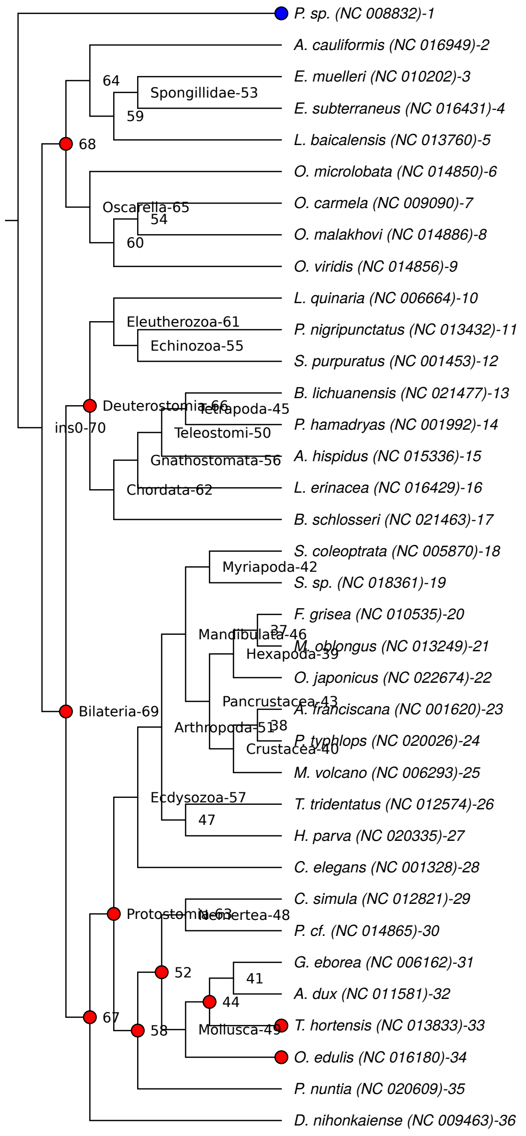

(a)

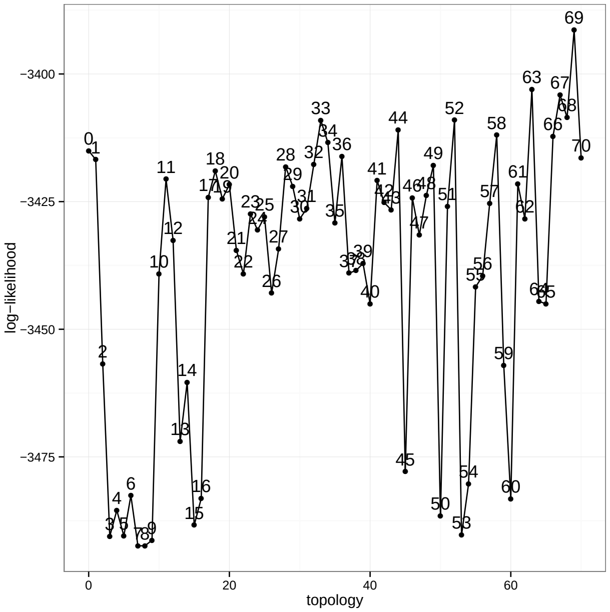

(b)

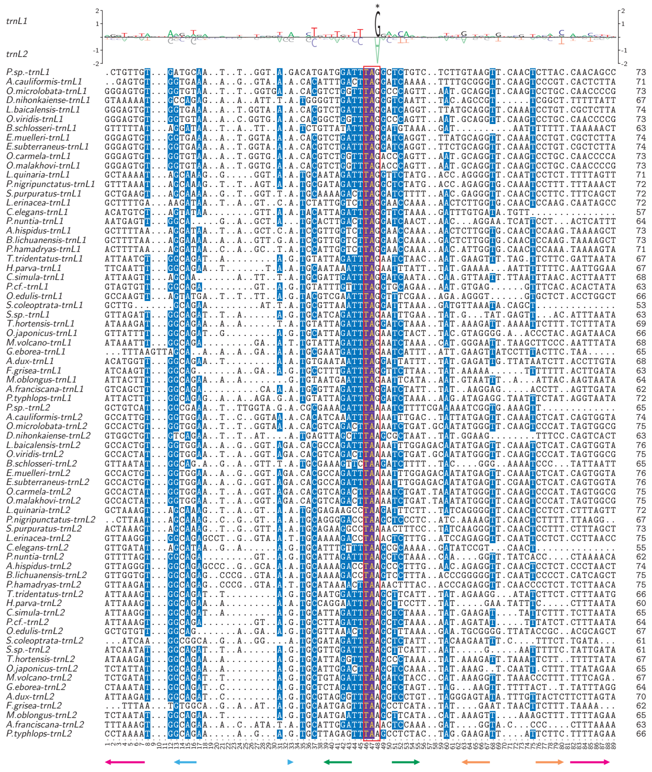

(c)

Figure 26: See Section 13

11.5 Porifera trnL2(uaa)⇝trnL1(uag)

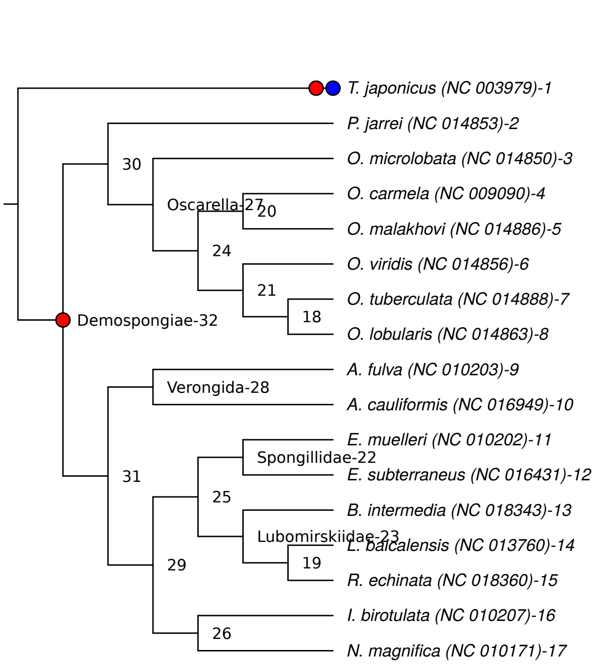

(a)

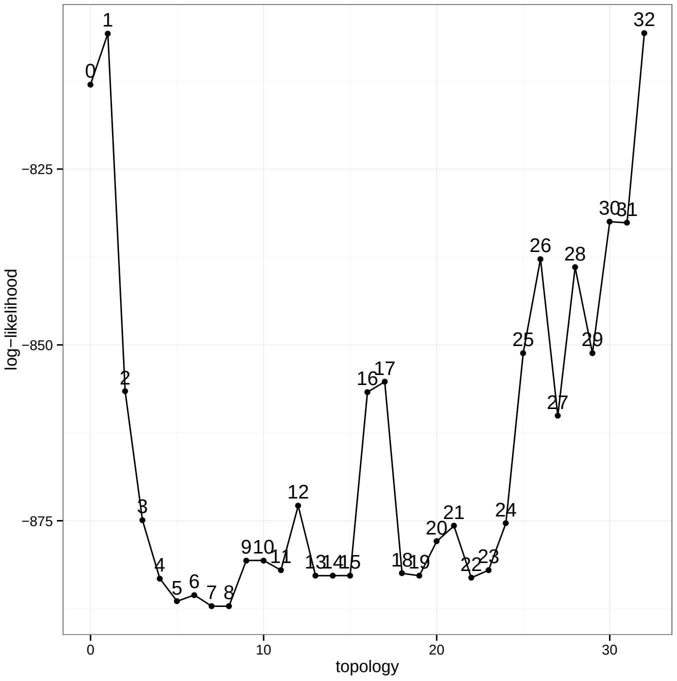

(b)

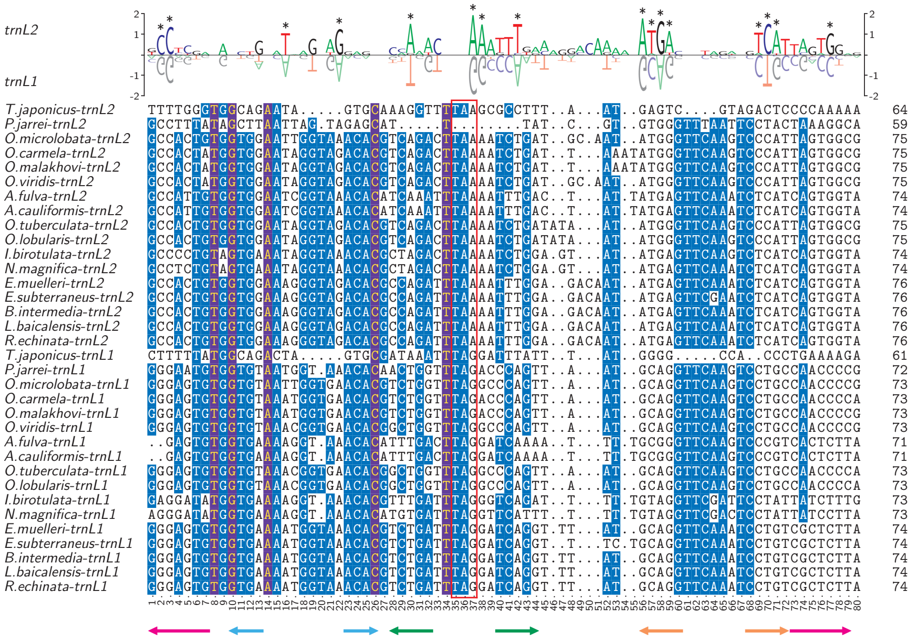

(c)

Figure 27: See Section 13

12 Amphibia trnK(uuu)⇝trnT(ugu)

Eleven K(UUU)⇝T(UGU) remolding candidates are found in the Amphibia (and a further one in Lepidosauria). The alignment of the amphibian *trnK* and *trnT* shows that the tRNAs are highly similar, i.e., 49 columns are well preserved (>50 %) with 15 highly conserved (>80 %), but 13 columns with tRNA specific nucleotides are present. The results of the `MLRD` method do not show increased log-likelihood values. The tRNAs are at extremely well preserved genomic positions within Chordata: in 2424 out of the 2621 chordate mitogenomes the genes *trnK* is between *cox2* and *atp8* and *trnT* is between *cob* and *trnP*. These adjacencies of *trnK* and *trnT* can be traced also in other phyla. In particular it holds that i) in 58 % of the Ambulacraria *trnK* has the same adjacencies and for *trnT* at least one of the adjacencies is preserved, ii) 69 % of the Ecdysozoa preserved the *cox2*-*trnK* and *trnT*-*trnP* adjacencies (and only an additional *trnD* is between *trnK* and *atp8*), and iii) in 33 % of the Porifera all but the *trnT*-*trnP* adjacency are present. This renders it unlikely that there was a K⇝T remolding in Bilateria and particularly not in Chordata.

Nevertheless, the *trnK* and *trnT* are very similar. The mean bitscore of the comparison of the two tRNAs, i.e., the mean of $S(K_i|T_i)$ for all metazoan species i, is 14.16 which is nearly as large as the mean bitscore for the two leucine tRNAs (15.19). That is, *trnK* is on average nearly as similar to *trnT* as the two leucine tRNAs. Hence, judging from the average bitscores there is no more reason to reject the possibility of K⇝T remolding(s) as early in metazoan evolution as the L2⇝L1 remolding(s).

Surprisingly, many other pairs of tRNAs show high mean bitscores, see Supplement 9.7. The largest values are 20.1 for *trnL2* and *trnY* and 22.3 for *trnE* and *trnG*. Eleven tRNA pairs with values larger than those of *trnL2* and *trnL1* are found, i.e., *trnH*-*trnR*, *trnH*-*trnY*, *trnH*-*trnP*, *trnH*-*trnD*, *trnA*-*trnR*, *trnA*-*trnD*, *trnA*-*trnP*, *trnA*-*trnE*, *trnG*-*trnR*, *trnL2*-*trnY*, and *trnE*-*trnG*. These might be pairs that are for some other reason highly similar or ancestrally remolded tRNAs. The minimal mean bitscore value is −9.4 found for *trnF* and *trnS1*.

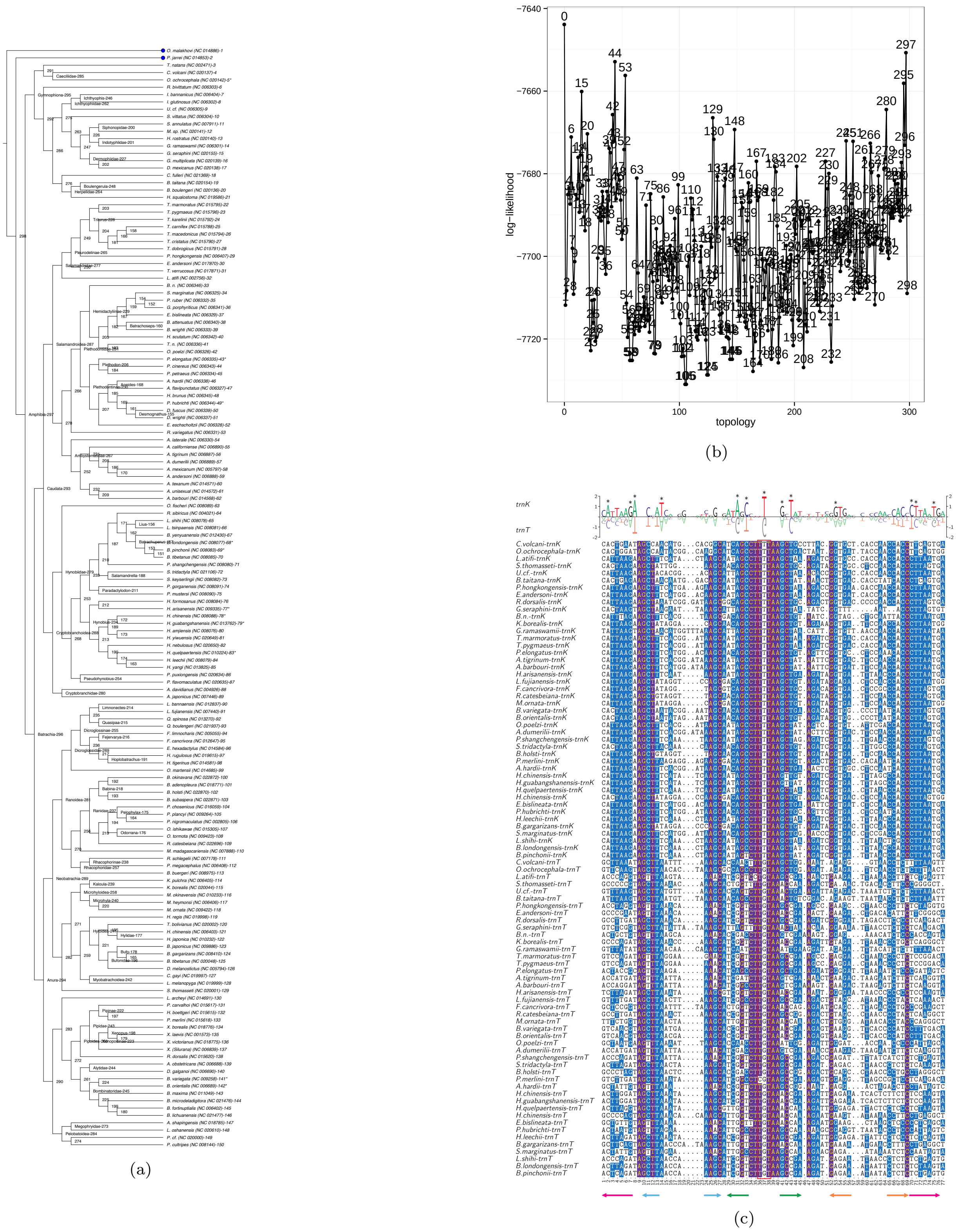

(a) (b) (c)

Figure 28: See Section 13

13 Caption for all figures

(a) Guide tree. Blue nodes mark outgroup species added to the input tree, red nodes mark tree rearrangements which yield a higher log-likelihood score compared to the start tree. Candidates are marked with a star.
(b) Log-Likelihood score calculated with `RAxML` for each tree topology. Numbers refer to the root node of the moved subtree. All leaves are first numbered, followed by a ascending numbering of the internal nodes based on the distance to the root node.
(c) Subfamily logo. Alignment columns with a sequence conservation $> 50\%$ ($> 80\%$) are highlighted in blue (purple). The anticodon is indicated by a red box. Colored arrows mark secondary structure elements, i.e. the stems of the acceptor arm (pink), D arm (light blue), anticodon arm (green), and T arm (orange).

References

[1] Nawrocki, E. P. and Eddy, S. R. (2013) `Infernal` 1.1: 100-fold faster RNA homology searches. *Bioinformatics,* **29**, 2933–2935.

[2] Jühling, F., Pütz, J., Bernt, M., Donath, A., Middendorf, M., Florentz, C., and Stadler, P. F. (2012) Improved systematic tRNA gene annotation allows new insights into the evolution of mitochondrial tRNA structures and into the mechanisms of mitochondrial genome rearrangements. *Nucleic Acids Res,* **40**, 2833–2845.

[3] Havird, J. C. and Santos, S. R. (2014) Performance of single and concatenated sets of mitochondrial genes at inferring metazoan relationships relative to full mitogenome data. *PLoS ONE,* **9**, e84080.

[4] Howe, K., Bateman, A., and Durbin, R. (2002) QuickTree: building huge Neighbour-Joining trees of protein sequences. *Bioinformatics,* **18**, 1546–1547.

[5] Larkin, M. A., Blackshields, G., Brown, N. P., Chenna, R., McGettigan, P. A., McWilliam, H., Valentin, F., Wallace, I. M., Wilm, A., Lopez, R., Thompson, J. D., Gibson, T. J., and Higgins, D. G. (2007) Clustal W and Clustal X version 2.0. *Bioinformatics,* **23**, 2947–2948.

[6] Huerta-Cepas, J., Dopazo, J., and Gabaldon, T. (2010) ETE: A python Environment for Tree Exploration. *BMC Bioinformatics,* **11**, 24.

[7] Beitz, E. (2006) Subfamily logos: visualization of sequence deviations at alignment positions with high information content. *BMC Bioinformatics,* **7**, 313.

[8] Beitz, E. (2000) TEXshade: shading and labeling of multiple sequence alignments using LATEX2$_\varepsilon$. *Bioinformatics,* **16**, 135–139.

[9] Kilpert, F. and Podsiadlowski, L. (2010) The Australian fresh water isopod (Phreatoicidea: Isopoda) allows insights into the early mitogenomic evolution of isopods. *Comp Bioch Phys D,* **5**, 36–44.

[10] Lin, F.-J., Liu, Y., Sha, Z., Tsang, L. M., Chu, K. H., Chan, T.-Y., Liu, R., and Cui, Z. (2012) Evolution and phylogeny of the mud shrimps (Crustacea: Decapoda) revealed from complete mitochondrial genomes. *BMC Genomics,* **13**, 631.

[11] Shi, H., Liu, R., Sha, Z., and Ma, J. (2012) Complete mitochondrial DNA sequence of *Stenopus hispidus* (Crustacea: Decapoda: Stenopodidea) and a novel tRNA gene cluster. *Mar Genomics,* **6**, 7–15.

[12] Rawlings, T. A., Collins, T. M., and Bieler, R. (2003) Changing identities: tRNA duplication and remolding within animal mitochondrial genomes. *P Natl Acad Sci USA,* **100**, 15700–15705.

[13] Higgs, P. G., Jameson, D., Jow, H., and Rattray, M. (2003) The Evolution of tRNA-Leu Genes in Animal Mitochondrial Genomes. *J Mol Evol,* **57**, 435–445.

[14] Segawa, R. D. and Aotsuka, T. (2005) The mitochondrial genome of the Japanese freshwater crab, *Geothelphusa dehaani* (Crustacea: Brachyura): Evidence for its evolution via gene duplication. *Gene,* **355**, 28–39.

[15] Cantatore, P., Gadaleta, M. N., Roberti, M., Saccone, C., and Wilson, A. C. (1987) Duplication and remoulding of tRNA genes during the evolutionary rearrangement of mitochondrial genomes. *Nature,* **329**, 853–855.

[16] Wang, X. and Lavrov, D. V. (2011) Gene recruitment - A common mechanism in the evolution of transfer RNA gene families. *Gene,* **475**, 22–29.

[17] Su, D., Lieberman, A., Lang, B. F., Simonović, M., Söll, D., and Ling, J. (2011) An unusual tRNAThr derived from tRNAHis reassigns in yeast mitochondria the CUN codons to threonine. *Nucleic Acids Res,* **39**, 4866–4874.
